# Aged Gut Microbiota Contributes to Cognitive Impairment and Hippocampal Synapse Loss in Mice

**DOI:** 10.1111/acel.70064

**Published:** 2025-04-12

**Authors:** Mingxiao Li, Jiaoqi Ren, Yiyang Bao, Wenjing Wei, Xuefei Yu, Xiaofang He, Mutalifu gulisima, Lili Sheng, Ningning Zheng, Jianbo Wan, Houguang Zhou, Ling Zhao, Houkai Li

**Affiliations:** ^1^ The Seventh People's Hospital of Shanghai University of Traditional Chinese Medicine China; ^2^ School of Pharmacy Shanghai University of Traditional Chinese Medicine Shanghai China; ^3^ Department of Geriatric Neurology of Huashan Hospital, National Clinical Research Center for Aging and Medicine Fudan University Shanghai China; ^4^ Department of Critical Care Medicine Renji Hospital, School of Medicine, Shanghai Jiao Tong University Shanghai China; ^5^ State Key Laboratory of Quality Research in Chinese Medicine Institute of Chinese Medical Sciences, University of Macau Taipa Macao SAR China; ^6^ Academy of Integrative Medicine Shanghai University of Traditional Chinese Medicine Shanghai China

**Keywords:** aging‐related cognitive decline, *Bifidobacterium pseudolongum*, microglia engulfment, synapse loss

## Abstract

Gut microbiota alteration during the aging process serves as a causative factor for aging‐related cognitive decline, which is characterized by the early hallmark, hippocampal synaptic loss. However, the impact and mechanistic role of gut microbiota in hippocampal synapse loss during aging remains unclear. Here, we observed that the fecal microbiota of naturally aged mice successfully transferred cognitive impairment and hippocampal synapse loss to young recipients. Multi‐omics analysis revealed that aged gut microbiota was characterized with obvious change in *Bifidobacterium pseudolongum* (*B.p*) and metabolite of tryptophan, indoleacetic acid (IAA) in the periphery and brain. These features were also reproduced in young recipients that were transplanted with aged gut microbiota. Fecal *B.p* abundance was reduced in patients with cognitive impairment compared to healthy subjects and showed a positive correlation with cognitive scores. Microbiota transplantation from patients who had fewer *B.p* abundances yielded worse cognitive behavior in mice than those with higher *B.p* abundances. Meanwhile, supplementation of *B.p* was capable of producing IAA and enhancing peripheral and brain IAA bioavailability, as well as improving cognitive behaviors and microglia‐mediated synapse loss in 5 × FAD transgenic mice. IAA produced from *B.p* was shown to prevent microglia engulfment of synapses in an aryl hydrocarbon receptor‐dependent manner. This study reveals that aged gut microbiota ‐induced cognitive decline and microglia‐mediated synapse loss that is, at least partially, due to the deficiency in *B.p* and its metabolite, IAA. It provides a proof‐of‐concept strategy for preventing neurodegenerative diseases by modulating gut probionts and their tryptophan metabolites.

## Graphical abstract

Li et al. revealed that aged gut microbiota induces cognitive decline and microglia‐mediated synapse loss. Fecal *Bifidobacterium pseudolongum* abundance was reduced in patients with cognitive impairment and microbiota transplantation from patients with fewer *B.p* abundance yielded worse cognitive behavior in mice. Supplementation with *B.p* improves aging‐related cognitive decline and prevents microglia engulfment of synapses via activation of IAA/AHR signaling.

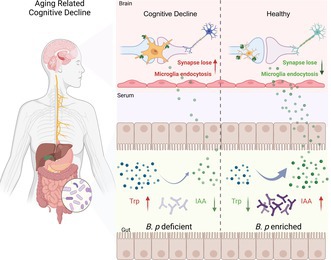

## 1 Introduction

Aging‐associated cognitive decline, as an important risk of developing neurodegenerative diseases, such as Alzheimer's Disease (AD), has an impact on the quality of life, overall well‐being, and carries substantial social and economic burdens (Mattson and Arumugam 2018; Tang et al. 2024; Guo et al. 2022; Aman et al. 2021). Biological hallmarks of aging, such as genomic instability, telomere attrition, epigenetic alterations, inflammation, metabolic dysregulation, dysregulated intercellular communication, cellular senescence, and so forth have been implicated as promotive factors of cognitive impairment (Rajendran and Krishnan 2024). Altered morphology and function of neurons, such as formation of neurofibrillary tangles, neurotrophic failure, neuronal loss, and synapse defects, are thought to be a common aspect of aging that contributes to cognitive decline (Zhang et al. 2024). Among them, synaptic loss is an early and invariant feature of cognitive decline, which is reported to be involved in excessive engulfment of glial cells (Zhong et al. 2023; Lewis 2023). However, the mechanism of aging‐related synapse loss remains incompletely understood.

Gut microbiota changes with age, characterized by reduced diversity, expansion of pathobionts, and alteration of small molecule metabolites (Yatsunenko et al. 2012; Induri et al. 2022). Gut microbiota is an indispensable “metabolic organ” for healthy aging (Ghosh et al. 2022), as well as the pathophysiology of aging, such as cognitive decline (D'Amato et al. 2020). In contrast, rejuvenation of gut microbiota results in the extension of healthspan and lifespan (Ma et al. 2020) and counteracts age‐associated impairments in cognitive behaviors (Boehme et al. 2021). Existing mechanisms by which gut microbiota and metabolites contribute to aging‐related cognitive decline include neuroinflammation, brain injury, and alterations in neurogenesis and neuron plasticity (Sharon et al. 2016; Erny et al. 2015; Borrego‐Ruiz and Borrego 2024). Yet, the mechanistic understanding of the gut‐brain axis, especially the role of gut microbiota in synapse loss, is not clearly elucidated.

In this study, we first performed fecal microbiota transplantation (FMT) from natural aged mice to young recipient mice to assess the impact of aged gut microbiota on cognitive behaviors and neuronal phenotypes. Then, the taxa and function of aged gut microbiota and serum metabolite profile were explored in both aged mice and clinically cognitively impaired patients. Our current results revealed that young recipient mice showed cognitive decline and microglia‐mediated synapse loss due to FMT from aged mice, which was characterized by fewer abundances of *Bifidobacterium pseudolongum* (*B.p*) and serum indoleacetic acid (IAA) content. Consistent results were obtained based on cognitive impairment patients, while supplementation of *B.p* improved cognitive behaviors and microglia‐mediated synapse loss through producing IAA. Our current findings add new evidence for the potential of improving aging‐related cognitive disorders through modulating the gut‐brain axis, especially *B.p* bacterium and IAA.

## 2 Results

### 2.1 Aged Gut Microbiota Induces Cognitive Decline and Hippocampal Synapse Loss in Young Recipients

**FIGURE 1:**
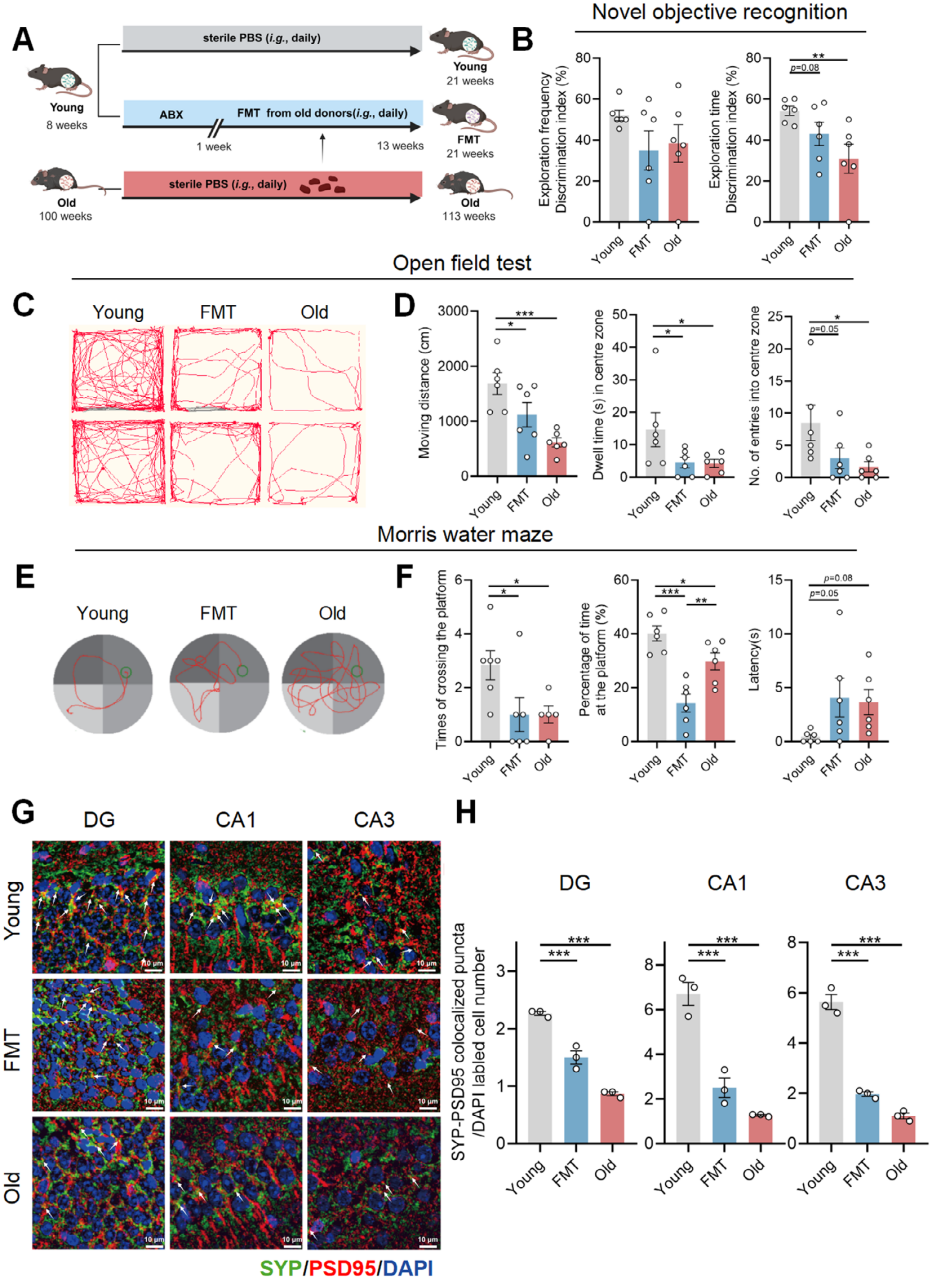
Aged gut microbiota induces cognitive decline and hippocampal synapse loss in young adult recipients. (A) Schematic diagram of transplanting fecal microbiota of elderly donors (100‐week‐old) to young adult recipients (8‐week‐old) for 12 consecutive weeks (*n* = 6/group). (B) Discrimination indices resulting from novel objective recognition test. (C and D) Representative move trajectories, distance (cm), dwell time (s) of staying in the center zone and numbers of entries into the center zone resulting from open field test. (E and F) Representative tracings, times of crossing the platform, percentage of time at the platform and latency (s) to reach the platform resulting from morris water maze test. (G) Representative immunofluorescence staining of SYP (green), PSD95 (red), and DAPI (blue) in the DG, CA1, and CA3 regions of hippocampus (scale bar: 10 μm). (H) Synapse number calculated from immunofluorescence staining. Statistical significance was analyzed using one‐way ANOVA with the method of Benjamini, Krieger, and Yekutieli for multiple‐group comparison (*p < 0.05, **p < 0.01, ***p < 0.005).

Given the well‐established evidence on the alteration of the gut microbiome during aging (Bárcena et al. 2019), we first investigated whether the aged gut microbiota could cause disorder in cognitive behavior in young mice by an FMT experiment. A group of young recipient mice (8‐week‐old) were transplanted gut microbiota from naturally aged mice (100‐week‐old) for a continued 12 weeks following a 1‐week antibiotic cocktail (ABX) treatment. Then, cognitive behaviors were evaluated among groups. The experiment scheme was presented in Figure 1A. Relative preference for the novel object calculated as the discrimination index in the exploration time was significantly reduced in aged donors, while showing a declined trend in young recipients (Figure 1B). Open field test showed a decrease in the locomotor parameters, including moving trajectories, distance, the duration, and times of entering into the center zone, in aged donors and young recipients when compared with young controls (Figure 1C,D). Morris water maze test revealed a significant reduction in the number of entering the platform and the duration of staying in the platform for aged donors and young recipients, while showing a longer latency period to reach the platform (Figure 1E,F).

Nissl staining revealed damaged neurons in the dentate gyrus (DG), cornu ammonis 1 (CA1) and CA3 regions of the hippocampus in old mice and young recipients (Figure S1A). Synapse loss is an early feature in the pathophysiology of neurodegenerative diseases that precedes neuronal death and represents a significant correlate of cognitive decline (Hong et al. 2016); synaptic deficit is considered a sign of the neuronal degenerative process. Synapse number counted by the colocalized puncta of pre‐synaptic (Synaptophysin, SYP) and post‐synaptic markers (Postsynaptic density protein 95, PSD95) was significantly lower in hippocampal DG, CA1, and CA3 regions of old mice and young recipients when compared with young controls (Figure 1G,H). We also assessed the expression of CYP1A1, an AHR target gene (Yokoyama et al. 2024), in the hippocampus of aging mice by using immunofluorescence staining. Compared to the young mice, the expression of CYP1A1 was significantly downregulated in both old and FMT groups, which demonstrated that AHR signaling decreases with aging (Figure S1E). The above results demonstrate that gut microbiota from naturally aged mice is sufficient to induce declined cognitive behaviors and hippocampal synapse loss in young recipient mice.

### 2.2 Aged Gut Microbiota Increases Hippocampal Glia‐Mediated Synapse Elimination

**FIGURE 2:**
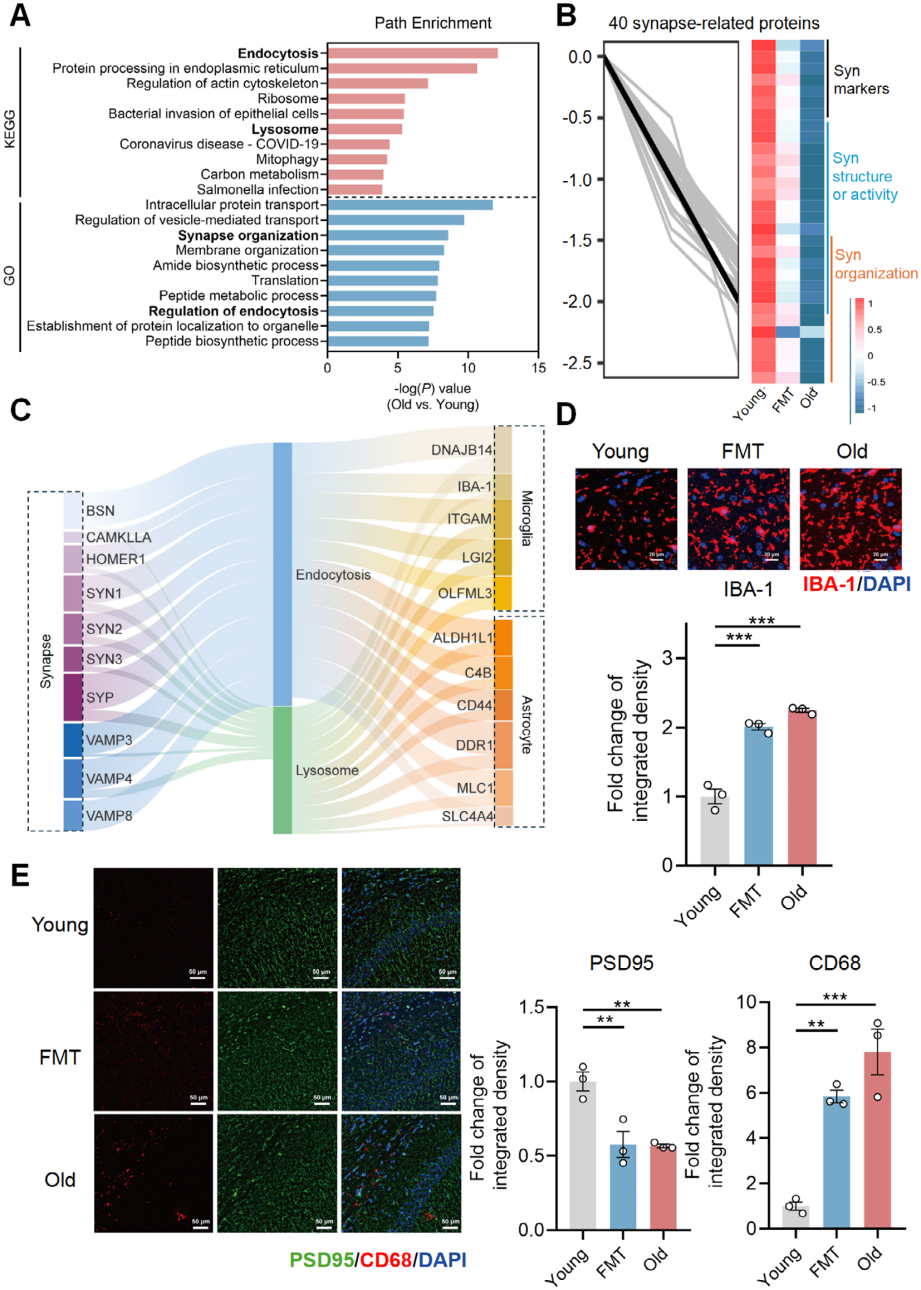
Aged gut microbiota dysregulates hippocampal synapse structure, activity and organization‐related proteome and induces microglia‐mediated synapse loss in young adult recipients. (A) Top 10 enriched functional pathways mapped by differential hippocampal proteins between young and elderly mice resulting from the GO or KEGG database. (B) The changes of hippocampal proteins related to synapse structure, activity and organization among young, FMT and old groups (*n* = 3/group). (C) The Sankey diagram displaying the relationship of synapse and microglia marker proteins with the process of endocytosis or lysosome, and the curved lines across the columns indicate the number of proteins with statistical significance resulting from the correlation analysis. (D) Representative immunofluorescence staining and calculated results of IBA1 (red) and DAPI (blue) in the CA1 region of hippocampus (scale bar: 20 μm). (E) Representative immunofluorescence staining and calculated results of PSD95 (green), CD68 (red) and DAPI (blue) in the CA1 region of hippocampus (scale bar: 50 μm). Statistical significance was analyzed using one‐way ANOVA with the method of Benjamini, Krieger, and Yekutieli for multiple‐group comparison (**p < 0.01, ***p < 0.005).

To testify to the effect of aged gut microbiota on the molecular phenotype of the brain, we analyzed mouse hippocampal proteome (*n* = 3/group). A total of 261 differentially expressed proteins (143 upregulated and 118 downregulated) were identified between the young and old groups and 45 differentially expressed proteins (35 upregulated and 10 downregulated) between the young and FMT groups (Figure S1B and Table S1). Top functional pathways enriched by differentially expressed proteins between young and old groups were mainly involved in synaptic dysfunctions, cellular transport, and catabolism (Figure 2A).

Given that glial engulfment is a key mechanism behind synapse loss (Chung and Barres 2012), the relationship between glia, synapse, and cellular processing proteins was analyzed. Forty proteins related to synapse structure, activity, and organization declined in FMT and old groups compared with young controls, while four proteins related to endocytosis, lysosome, and phagocytosis were increased (Figure 2B and Table S2). We observed a decline in synapse indices and an increase in reactive glial markers associated with higher levels of endocytosis and lysosome proteins (Figure 2C and Table S3). It is further supported by immunofluorescence (IF) data that ionized calcium‐binding adaptor molecule 1 (IBA‐1, reactive microglia marker) and cluster of differentiation 68 (CD68, microglial phagocytic marker) were highly expressed in the hippocampal CA1 region of old mice and young recipients, while postsynaptic density protein 95 (PSD95) was deficiently expressed (Figure 2D,E). Phagocytic and synaptic markers were also validated by qPCR, and the changes were consistent with those detected in proteomic analysis (Figure S2). The above findings indicate a promotive role of aged gut microbiota in glial engulfment of synapse loss.

### 2.3 Aged Gut Microbiota Is Characterized by Deficiency of *B.p* and Low Level of Indoleacetic Acid

**FIGURE 3:**
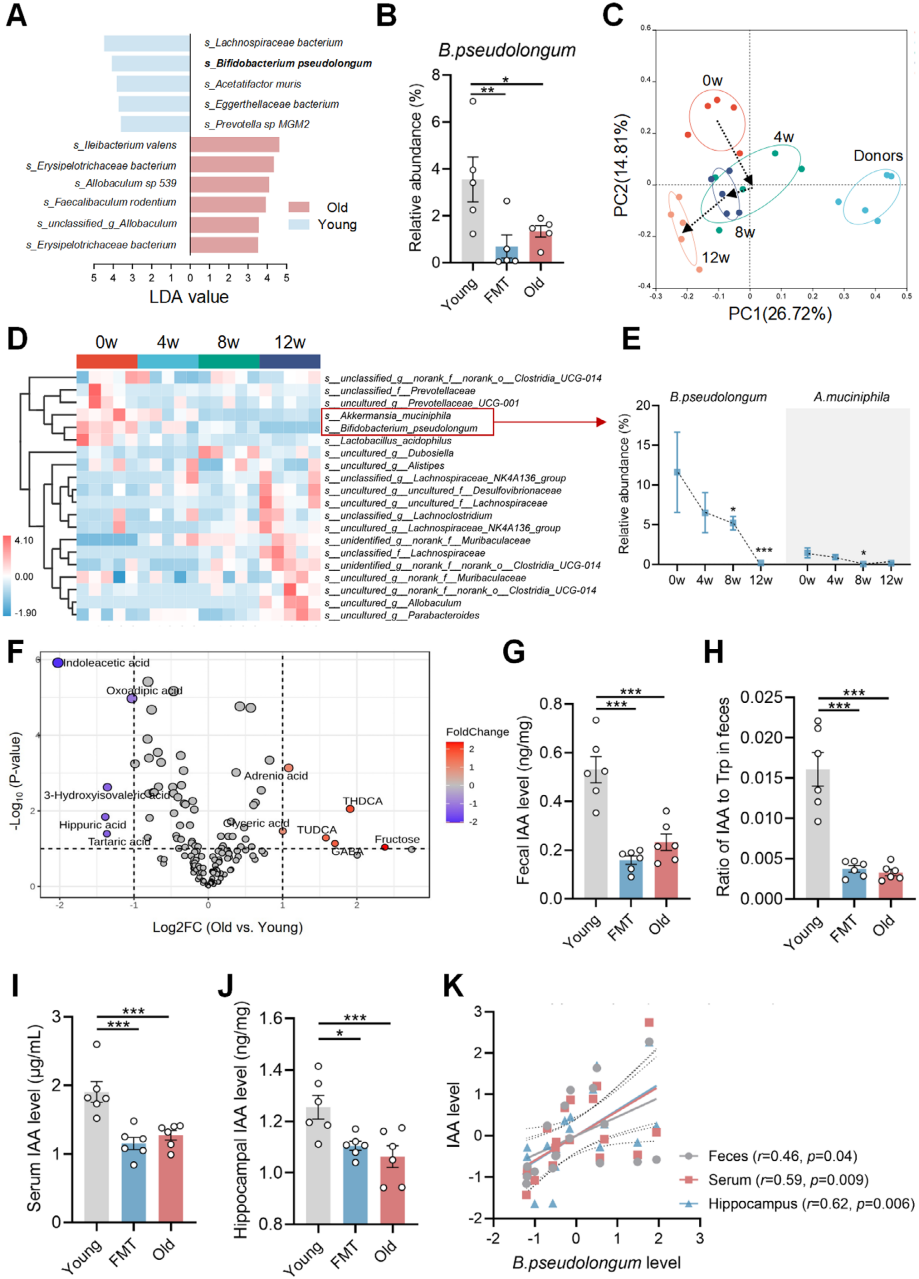
Aged gut microbiota deficient with *B.p* is associated with lower levels of IAA production in the gut and bioavailability in the periphery and brain in young adult recipients. (A) Differential bacterial species between young and old groups resulted from metagenome analysis. (B) Relative abundance of *B.p* among young, FMT and old groups (*n* = 5/group). (C) OTU‐based PCoA plot of gut microbiota β‐diversity resulted from 16S rRNA sequencing analysis (*n* = 5/group). (D) Heatmap of differential fecal bacteria species in young mice receiving 0, 4, 8 and 12‐week of fecal microbiota of elderly donors (*n* = 5/group). (E) Dynamic changes in relative abundances of *B.p* and *Akkermansia muciniphila* in young recipients during the microbiota transplantation period. (F) Volcano plot of differential metabolites between young and old groups resulted from serum metabolomic analysis (*n* = 6/group). (G–J) The IAA levels in feces, serum and hippocampus among young, FMT and old groups (*n* = 6/group). (H) Ratio of IAA to Trp in feces among young, FMT and old groups (*n* = 6/group). (K) Pearson's correlation between the abundance of *B.p* and the levels of fecal, serum and hippocampal IAA. Statistical significance was analyzed using Student *t*‐test for two‐group comparison and one‐way ANOVA with the method of Benjamini, Krieger and Yekutieli for multiple‐group comparison (*p < 0.05, **p < 0.01, ***p < 0.005).

To characterize the structure and function of the aged gut microbiota, we sequenced fecal metagenome and metabolomic sequencing on cecal content of mice. Fecal metagenomic data revealed a differential taxonomic profile between young and old mice, among which the relative abundance of *Bifidobacterium pseudolongum* (*B.p*) was notably reduced in old donors and young recipients (Figure 3A,B and Table S4). Fecal 16S rRNA sequence revealed that the gut microbial pattern of young recipients dynamically altered during the duration of FMT, in which process *B.p* and *Akkermansia muciniphila* were lowered gradually (Figure 3C–E). We then performed absolute quantification of *B.p* in fecal samples from mice that had received FMT for 0, 4, 8, and 12 weeks. The results were consistent with the 16S rRNA gene sequencing data, indicating a gradual decrease of *B.p* during the duration of FMT (Figure S3A,B).

Serum metabolomic analysis showed 50 differential metabolites between young and old groups, mainly mapped into several amino acid metabolism paths (Figure S1D,E and Table S5). Of note, indoleacetic acid (IAA), a byproduct of microbial tryptophan (Trp) metabolism (Roager and Licht 2018), was the most significantly decreased metabolite in old donors and also lowered in FMT mice (Figure 3F,G). The declined ratio of IAA to Trp in feces in old and FMT groups also suggests a lower bacterial IAA‐producing capability (Figure 3H). IAA was also found to be reduced in serum and hippocampus in old donors and young recipients compared with young controls (Figure 3I,J). The abundance of *B.p* had a positive relationship with fecal, serum, and hippocampal IAA concentrations (Figure 3K). Omics data demonstrate that a transferable aged gut microbiota featured by a significant loss of *B.p* is strongly associated with a lower level of IAA.

### 2.4 Microbiota Transplantation From Patients With Lower *B.p* Abundance Yielded Worse Cognitive Behaviors

**FIGURE 4:**
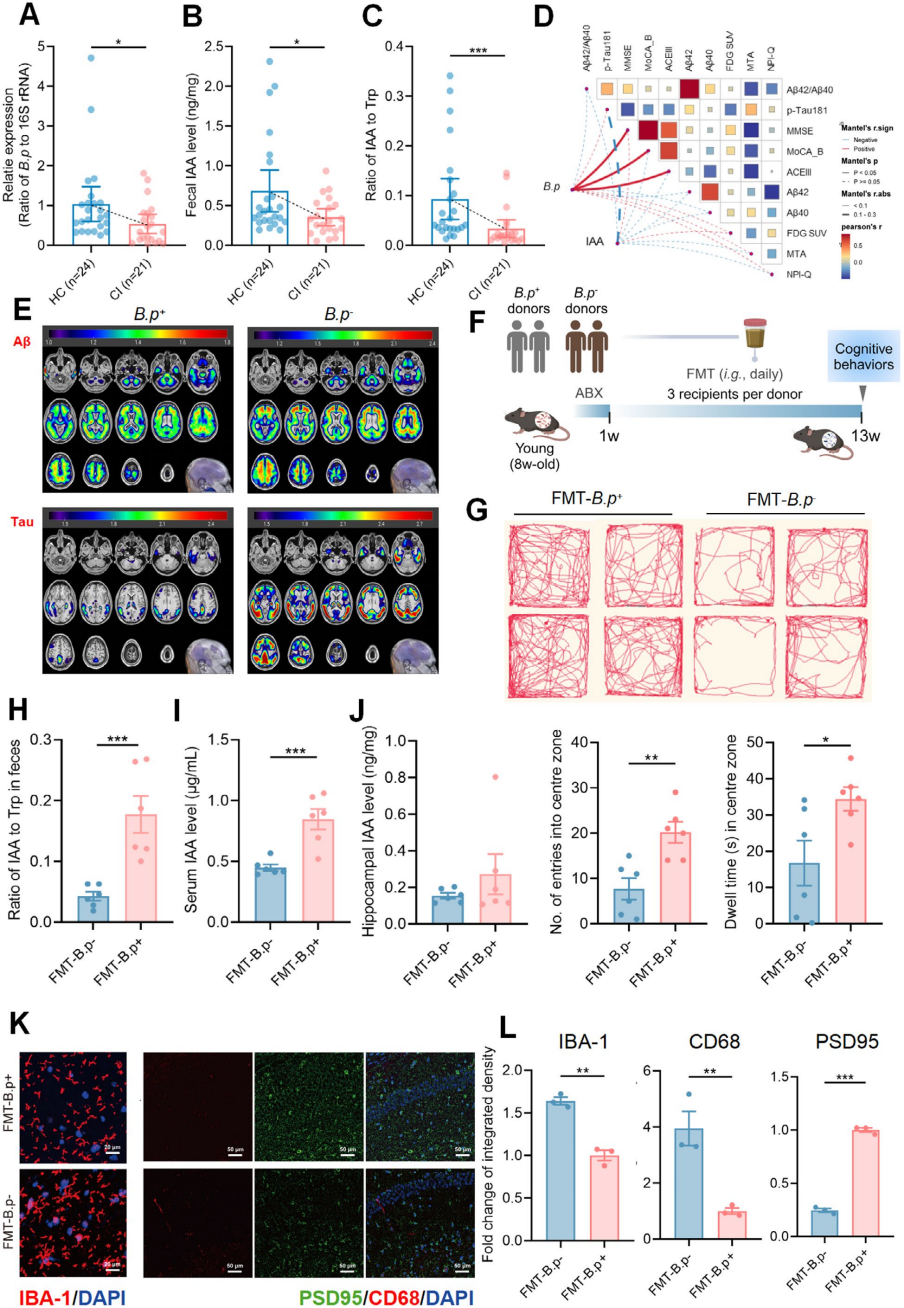
Microbiota transplantation from patients with fewer *B.p* abundance yielded worse cognitive behaviors. (A) Relative abundance of fecal *B.p* in patients with cognitive impairment (CI, *n* = 21) and healthy controls (HC, *n* = 24) resulted from species‐specific PCR analysis. (B, C) The levels of IAA and ratio of IAA to Trp in feces resulted from QTRAP‐based quantification analysis. (D) Pearson's correlation analysis of cognitive indices with fecal *B.p* abundance or fecal IAA level in patients with CI. (E) Representative Aβ‐PET and Tau‐PET images of *B.p* + and *B.p*‐ AD patients. (F) Schematic diagram of transplanting *B.p*‐ or *B.p* + fecal microbiota of CI patients to young adult recipients (8‐week‐old) for 12 consecutive weeks (*n* = 6/group). (G) Representative move trajectories, dwell time (s) of staying in the center zone and numbers of entries into the center zone resulted from open field test. (H–J) Ratio of IAA to Trp in feces and levels of IAA in serum and hippocampus between FMT groups. (K) Representative immunofluorescence staining and calculated results of IBA1 (red) and DAPI (blue) in the CA1 region of hippocampus (scale bar: 20 μm). (L) Representative immunofluorescence staining and calculated results of PSD95 (green), CD68 (red) and DAPI (blue) in the CA1 region of hippocampus (scale bar: 50 μm). Statistical significance was analyzed using Mann–Whitney test for two‐group comparison for clinical data analysis and unpaired Student *t*‐test for two‐group comparison for animal data analysis (*p < 0.05, **p < 0.01, ***p < 0.005).

To further confirm the association of *B.p*‐deficient microbiota and its lowered IAA production level with aging‐related cognitive decline in human beings, we recruited 21 patients with cognitive impairment (CI, 9 MCI patients, 12 ad patients) and 24 healthy controls (HC). The characteristics of recruited patients were shown in Table S6. Species‐specific qPCR analysis revealed a reduction in the relative abundance of *B.p* in the feces of CI patients compared with HC (Figure 4A). Fecal IAA level and the ratio of IAA to Trp were also lower in CI patients (Figure 4B,C). Spearman's correlation analysis revealed that fecal *B.p* level was positively associated with minimum mental state examination (MMSE), Montreal cognitive assessment basic (MoCA‐B) and Addenbrooke's cognitive examination III (ACEIII) scores (Figure 4D), which are widely used to assess cognitive function (Jack Jr et al. 2024). Fecal IAA concentration showed a reverse association with the level of plasma phosphorylated‐tau181 (*p*‐tau181) (Figure 4D), a promising biomarker of amyloid burden (Moscoso et al. 2021). Further, we also found that patients with lower *B.p* abundance showed more severe Aβ and Tau deposition on PET images (Figure 4E).

To further investigate the role of *B.p*‐deficient human gut microbiota in affecting cognitive function, we transplanted *B.p*‐enriched or *B.p*‐deficient fecal microbiota from CI patients into male pseudo‐germ‐free mice (8‐week‐old) for 12 weeks after 1‐week ABX‐induced gut microbiota elimination (2 donors for each group, *n* = 6 mice/group) (Figure 4F). The open field test showed recipients with *B.p*‐deficient microbiota displayed a significant reduction in moving trajectories and the number of entries into the centre zone compared with those receiving *B.p*‐enriched microbiota, while showing a slight increase in the dwell time in the centre zone (Figure 4G). The ratio of IAA to Trp in feces was significantly lowered in *B.p*‐deficient microbiota recipients, serum IAA level was elevated, and hippocampal IAA showed a slight increase (Figure 4H–J). IF staining data showed that *B.p*‐deficient human microbiota transplantation resulted in higher expressions of IBA‐1 and CD68 in the recipient hippocampus while showing a lower level of PSD95 (Figure 4K,L). Clinical and animal results indicate that *B.p*‐deficient human gut microbiota with a low IAA‐producing capability can induce cognitive decline and increase the level of microglia engulfment of synapses.

### 2.5 *B.p* Supplementation Increases IAA Production and Its Bioavailability

**FIGURE 5:**
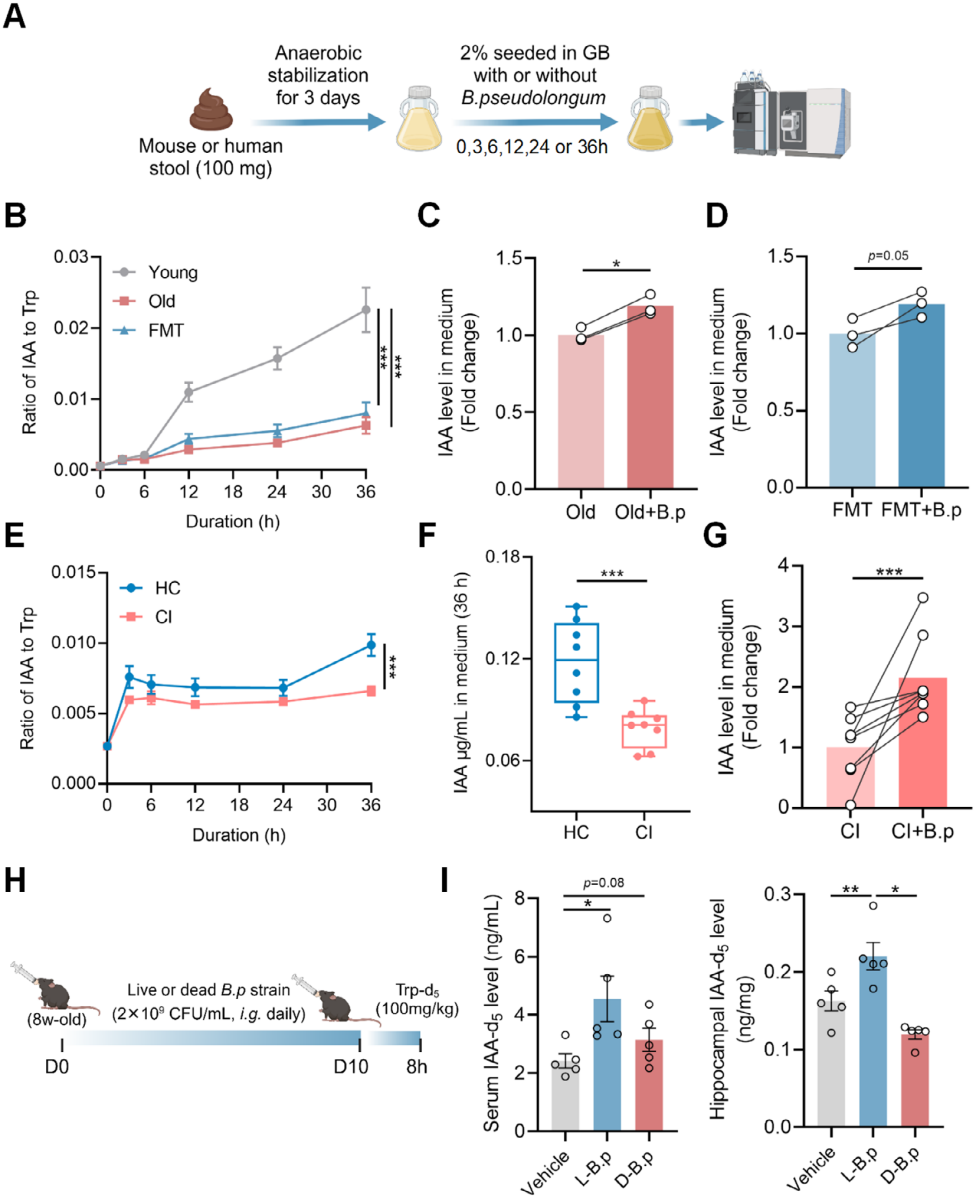
*B.p* increases the level of bacterial IAA production in vitro and elevates the level of bioavailability in the periphery and brain in vivo. (A) Schematic diagram of assessing the IAA‐producing activities of mouse or patient fecal microbiota alone or co‐cultured with the *B.p* strain (2 × 10^9^ CFU/mL) in different time course under an anaerobic condition. (B, E) Ratio of IAA to Trp in mediums of mouse (*n* = 3) or patient (*n* = 8) fecal microbiota cultured under 0, 3, 6, 12, 24 and 36 h. (C, D, G) Fold changes of IAA in mediums of mouse or patient fecal microbiota alone or co‐cultured with the *B.p* strain. (F) The IAA level in the 36 h cultural medium of patient fecal microbiota. (H) Schematic diagram of assessing bacterial IAA‐producing activity in SPF male mice colonized with 10 days of live or dead *B.p* strains (2 × 10^9^ CFU/mL). (I) Serum and hippocampal d_5_‐IAA bioavailability in mice colonized with live or dead *B.p* strains. Statistical significance was analyzed using unpaired Student *t*‐test for two‐group comparison and one‐way ANOVA with the method of Benjamini, Krieger, and Yekutieli for multiple‐group comparison (*p < 0.05, **p < 0.01, ***p < 0.005).

It remains unclear whether *B.p* is capable of producing IAA. We first cultured *B.p* alone and found that *B.p* could metabolize tryptophan directly to IAA, while with tryptophan added to the medium, the IAA‐producing capability was higher (Figure S3C). To examine the effect of IAA‐producing capability for *B.p*, we cultured mouse or human fecal microbiota alone or supplemented with *B.p* (2 × 10^9^ CFU/mL) under anaerobic conditions (Figure 5A). Results showed that the IAA‐producing capability of fecal microbiota isolated from FMT and old mice were lower than that of young mice upon 12, 24, and 36 h of culture (Figure 5B). Co‐culture with *B.p* upregulated the IAA level in the culture medium of fecal microbiota isolated from old and FMT mice (Figure 5C,D). Fecal microbiota of CI patients showed a lower level of IAA‐producing capability after 36 h culture than that of HC subjects, whilst the IAA level was significantly elevated in patients' fecal microbiota co‐cultured with *B.p* (Figure 5E–G). We also examined the impact of *B.p* on peripheral and brain IAA bioavailability in vivo by colonizing live or heat‐killed *B.p* (2 × 10^9^ CFU/mL) followed by oral administration of Trp‐d_5_ (Figure 5H). We found that the live strain but not the dead strain can result in higher levels of IAA‐d_5_ bioavailability in mouse serum and hippocampus (Figure 5I). The above data illustrate that *B.p* is capable of producing IAA and enhance peripheral and brain IAA bioavailability.

### 2.6 *B.p* Supplementation Improves Cognitive Decline and Microglia‐Mediated Synapse Loss

**FIGURE 6:**
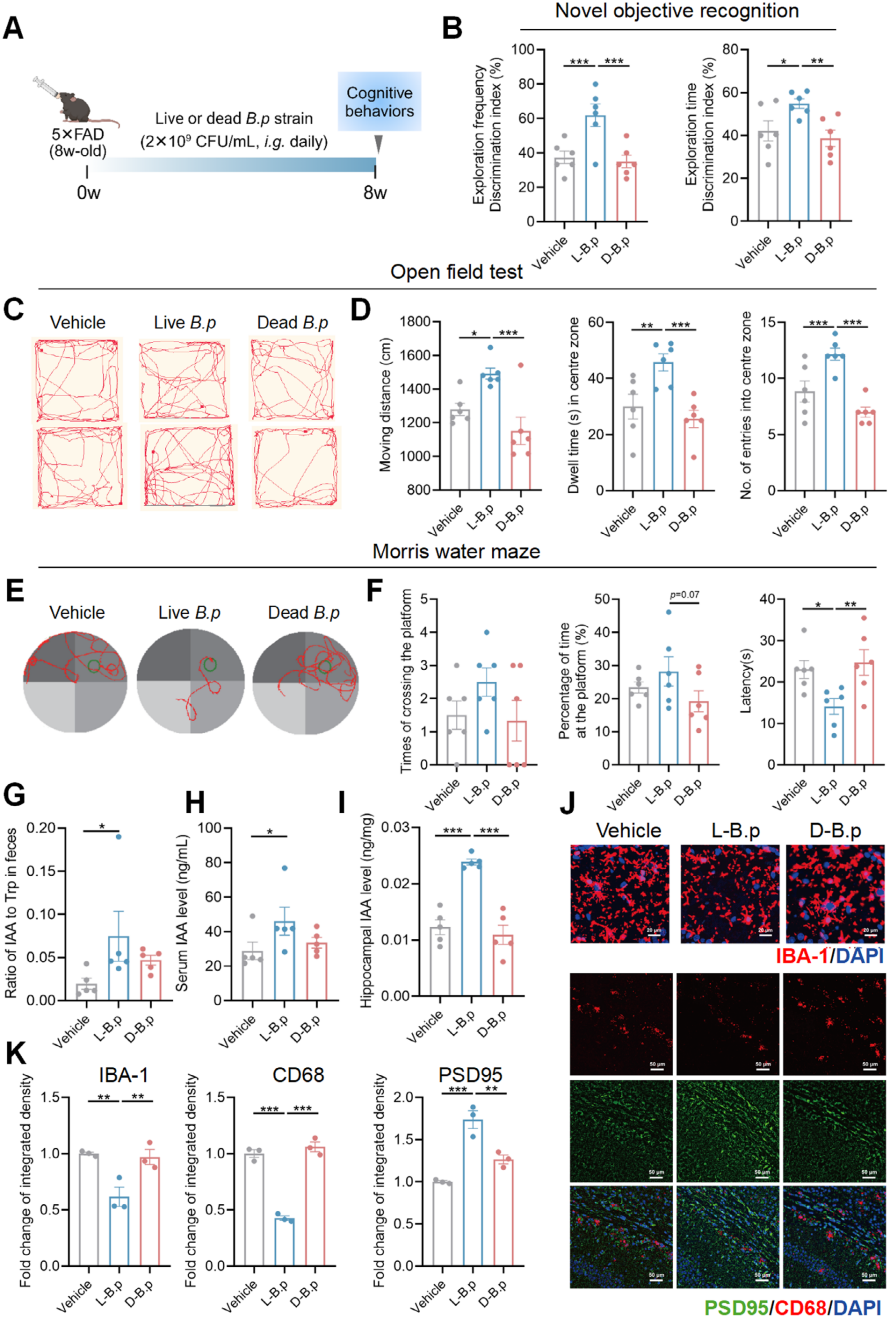
*B.p* improves cognitive decline and microglia‐mediated synapse loss in 5 × FAD transgenic mice, while enhancing peripheral and brain IAA bioavailability levels. (A) Schematic diagram of supplementing live or dead *B.p* strains into male 5 × FAD transgenic mice for 8 consecutive weeks (*n* = 6/group). (B) Discrimination indices resulted from Novel objective recognition test. (C, D) Representative move trajectories, distance (cm), dwell time (s) of staying in the center zone and numbers of entries into the center zone resulting from Open field test. (E, F) Representative tracings, times of crossing the platform, percentage of time at the platform and latency (s) to reach the platform resulting from Morris water maze test. (G–I) Ratio of IAA to Trp in feces and levels of IAA in serum and hippocampus among groups. (J) Representative immunofluorescence staining of IBA1 (red) and DAPI (blue) in the CA1 region of hippocampus (scale bar: 20 μm). (K) Representative immunofluorescence staining of PSD95 (green), CD68 (red) and DAPI (blue) in the CA1 region of hippocampus (scale bar: 50 μm). (L) Calculated results of IBA‐1, PSD95, and CD68. Statistical significance was analyzed using one‐way ANOVA with the method of Benjamini, Krieger, and Yekutieli for multiple‐group comparison (*p < 0.05, **p < 0.01, ***p < 0.005).

To testify if IAA‐producing *B.p* could protect aging‐related brain pathophysiology and cognitive function, we separately colonized live and dead *B.p* strains (2 × 10^9^ CFU/mL) into 5 × FAD transgenic mice (*n* = 6/group) for 8 consecutive weeks in the way of oral gavage (Figure 6A). Behavioral data showed that live *B.p* enhanced the exploration time and frequency, and increased the move distance, the number of entries, and dwell time in the center zone in 5 × FAD mice (Figure 6B–D), while significantly lowering the latency period to reach the target platform (Figure 6E,F). Live *B.p* but not the dead strain resulted in an increased IAA‐producing level in the gut of 5 × FAD mice, also elevated IAA bioavailability in serum and hippocampus (Figure 6G–I). IF staining results showed lower protein expressions of IBA‐1 and CD68 but a higher CYP1A1 (AHR signaling target gene) expression in the hippocampus of 5 × FAD mice treated with live *B.p* than those colonized with dead strains, whereas PSD95 protein expression was increased (Figure 6J–L and Figure S4A). However, the dead *B.p* strain did not affect cognitive behaviors and hippocampal microglia‐synapse markers.

Additionally, we observed that live *B.p* strain had no obvious impact on prolonging the lifespan and heat stress of *Caenorhabditis elegans* but increased the frequency of body oscillation and locomotion (Figure S4B–E), suggesting that *B.p* has a function to increase exercise vitality. The above data suggest a beneficial role of *B.p* against aging‐related cognitive dysfunction and microglia‐mediated synapse loss.

### 2.7 IAA Prevents Microglia Engulfment of Synapses in an Aryl Hydrocarbon Receptor‐Dependent Manner

**FIGURE 7:**
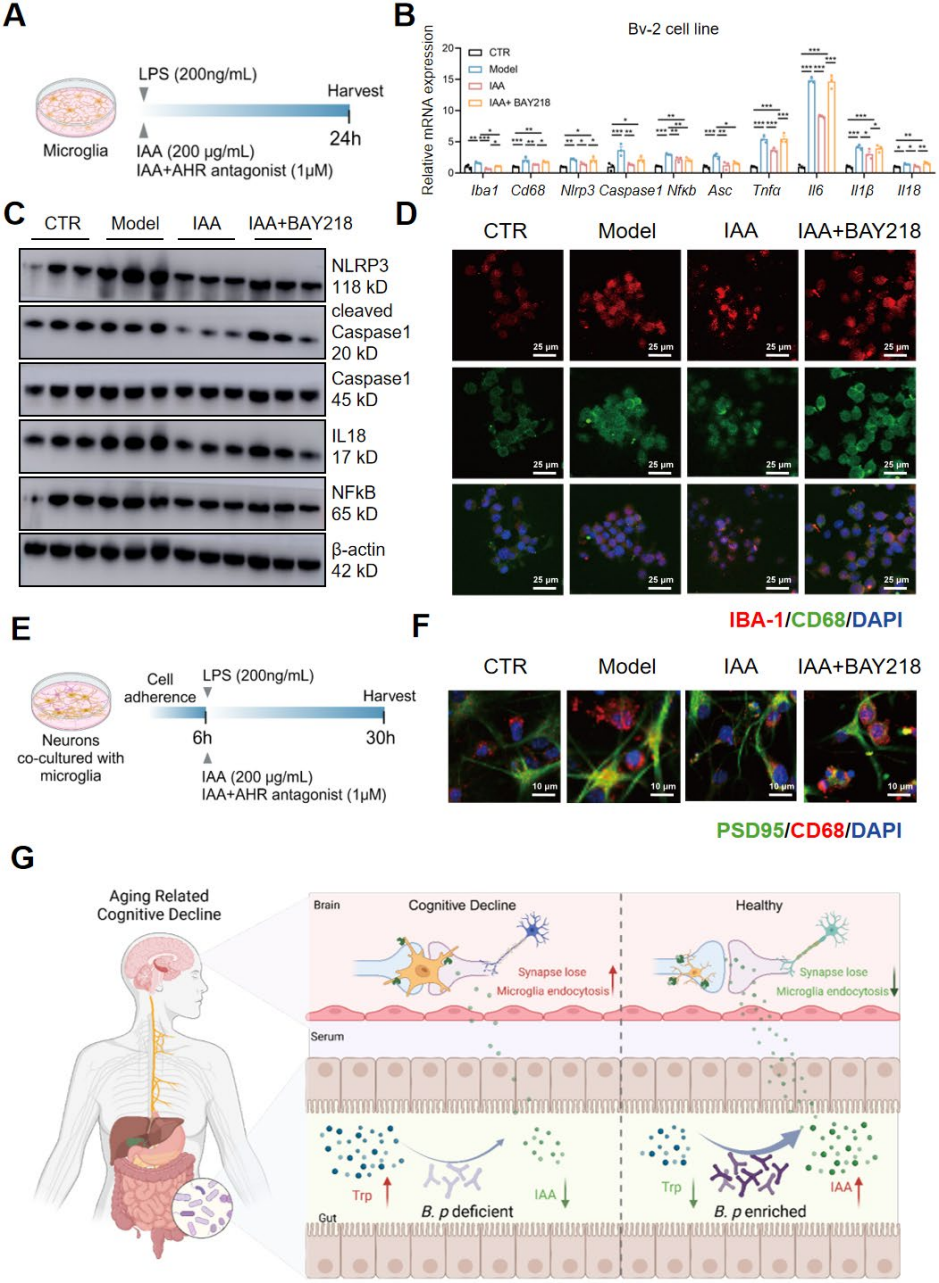
IAA inhibits the induction of microglia activation and microglia‐mediated synapse loss by LPS in an Aryl hydrocarbon receptor(AHR)‐dependent manner. (A) Schematic diagram of examining the effects of IAA (200 μg/mL) alone or combined with the AHR antagonist (BAY218, 1 μM) on LPS‐induced microglia Bv‐2 cell activation (*n* = 3/group). (B) Relative mRNA expression of genes related to microglia activation and inflammation in Bv‐2 microglia (*n* = 3/group). (C) The expressions of proteins associated with inflammation in Bv‐2 microglia (*n* = 3/group). (D) Representative immunofluorescence staining of IBA1 (red), CD68 (green) and DAPI (blue) in the section of the Bv‐2 cell line (scale bar: 25 μm). (E) Schematic diagram of examining the effects of IAA (200 μg/mL) alone or combined with the AHR antagonist (BAY218, 1 μM) on primary neuronal synapse co‐cultured with microglia Bv‐2 cells (*n* = 3/group). (F) Representative immunofluorescence staining of PSD95 (green), CD68 (red) and DAPI (blue) in neuron–microglia co‐cultural sections (scale bar: 10 μm). (G) Schematic overview of the action mechanism by which *B.p* strain supplementation improves aging‐related cognitive decline and microglia‐mediated synapse loss. Statistical significance was analyzed using one‐way ANOVA with the method of Benjamini, Krieger, and Yekutieli for multiple‐group comparison (*p < 0.05, **p < 0.01, ***p < 0.005).

IAA, as a natural ligand of aryl hydrocarbon receptor (AHR), has an inhibitory effect on neuroinflammation (Guo et al. 2023). Yet, the role of IAA in microglia‐mediated synapse loss remains unclear. To clarify the role of IAA in microglia‐mediated synaptic loss, we conducted studies in microglia and neuronal models in vitro. We observed that the induction of *Iba‐1* gene expression in LPS‐treated microglia Bv‐2 cell line was significantly attenuated by 50, 100, and 200 μg/mL of IAA, while LPS‐enhanced *Cd68* gene expression was reversed by 200 μg/mL of IAA only (Figure S5A,B), suggesting 200 μg/mL is an effective dose of IAA against LPS‐induced microglial activation. We further confirmed that 200 μg/mL IAA indeed inhibits LPS‐activated Bv‐2 microglia cells, proinflammatory and engulfing signals, which presented as gene of *Iba‐1*, *Cd68*, *Nlrp3*, *Caspase 1*, *Nfkb*, *Asc*, *Il6*, *Il1β* in IAA group were downregulated (Figure 7B), and the protein of NLRP3, Cleaved‐Caspase1, IL18, and NFκB in IAA group were downregulated compared to model group (Figure 7C and Figure S5C). Immunofluorescence results also showed that IAA downregulated IBA1 and CD68 expressions in microglia (Figure 7D and Figure S5D,E). Nevertheless, reactive microglia and its proinflammatory and engulfing indices showed significant upregulation in Bv‐2 cells treated with the combination of IAA and AHR antagonist BAY218 (1 μM) compared with the IAA group (Figure 7B–D and Figure S5C–E).

To further clarify if IAA attenuates microglia engulfment of synapses, we cocultured primary neurons with isolated microglia following a published protocol (Li et al. 2023) (Figure 7E). IF staining results showed that the number of PSD95 and CD68‐dual positive spots was enhanced by LPS, which was reduced by IAA and showed no difference with the combination of IAA and BAY218 (Figure 7F and Figure S5F). The above cellular experiments suggest that IAA attenuates LPS‐induced microglia engulfment of synapses in an AHR‐dependent manner.

## 3 Discussion

This study reveals that aged gut microbiota induces cognitive decline and microglia‐mediated synapse loss. Fecal *B.p* abundance was reduced in patients with cognitive impairment and microbiota transplantation from patients with fewer *B.p* abundance yielded worse cognitive behavior in mice. Supplementation with *B.p* improves aging‐related cognitive decline and prevents microglia engulfment of synapses via activation of IAA/AHR signaling.

Aging is a complex and multifactorial intertwined biological process. Genomic instability, telomere shortening, epigenetic changes, and other factors interact with each other to drive the aging process (Wu et al. 2023; Liao et al. 2023). Due to the complexity of the mechanisms, strategies to prolong life and treat aging‐related diseases are numerous. The results of several studies highlight the causal relationship between aging and intestinal dysbiosis. Our previous study also confirmed that gut microbiota remodeling reversed aging‐associated inflammation and dysregulation of systemic bile acid homeostasis (Central and peripheral), exerted hepatoprotective effects, and improved glucose sensitivity, hepatosplenomegaly, inflammation, antioxidant capacity, and the intestinal barrier (Ma et al. 2020, 2023, 2024). Aged gut microbiota impairs spatial learning and memory of young recipients by affecting hippocampal synaptic plasticity‐ and neurotransmission‐related proteins (D'Amato et al. 2020). Transmission of AD‐associated gut bacteria dysbiosis induces Tau phosphorylation and cognitive impairment in mouse recipients (Zhang et al. 2023). Conversely, rejuvenation remodeling of gut bacteria in elderly mice partially reversed these deleterious effects (Boehme et al. 2021; Ma et al. 2023; Parker et al. 2022). Beyond inducing impaired cognitive behaviors, we observed that the aged gut microbiota with fewer *B.p* is sufficient to dysregulate hippocampal glia–neuron proteome in young mice, particularly lowering synapse structure/activity and organization‐related proteins but upregulating glia engulfment‐related protein levels.

Gut microbiota shifts with age; in particular, the loss of *Bifidobacterium* species has been linked with mild cognitive impairment or AD in cohort studies (Zhuang et al. 2018; Li et al. 2019; Liu et al. 2019; Zhu et al. 2022). *B.p* and its derived metabolites (e.g., acetate and inosine) have been shown to enhance host antiviral response (Niu et al. 2023), prevent non‐alcoholic fatty liver disease‐related hepatocellular carcinoma (Song et al. 2023), and modulate response to checkpoint inhibitor immunotherapy (Mager et al. 2020). However, *B.p* has not been reported in aging and cognitive‐related diseases. In our case, taxa‐based dynamic analysis of gut microbiota reveals that *B.p* is identified as one of the most abundant bacteria that can be continuously reduced with the progress of aged gut microbiota transfer. By manipulating *B.p* abundances with transplantation of human microbiota or single strain colonization, we proved a beneficial role of *B.p* in maintaining cognitive functions and hippocampal microglia‐synapse phenotypes for the first time. Consolidating our research, *Bifidobacterium*, especially *B.p* have great potential to ameliorate aging‐related symptoms.

Enriched environment training combined with *Bifidobacterium shortum* CCFM1025 significantly improved β‐amyloid‐induced cognitive dysfunction and suppressed neuroinflammation in mice (Zhu et al. 2023). *Bifidobacterium lactis* Probio‐M8 reduces whole‐brain Aβ plaque load and protects against gut dysbiosis in the APP/PS1 mouse model (Cao et al. 2021). *Bifidobacterium animalis* subspecies lactis BPL1 and its lipocholic acid modulate lifespan and improve age/stress‐related behaviors in *C. elegans* (Balaguer et al. 2023). Of interest, *Akkermansia muciniphila*, another species with a decrease in the period of transferring aged gut microbiota, has been documented to prevent cognitive deficits in sleep‐deprived mice, the mechanism of which is also involved in the modulation of microglial engulfment of synapses (Li et al. 2023). Previous and our findings collectively indicate that synapse is an important bacterial target of protecting cognitive functions, highlighting the potential implication of probiont‐related strategies in managing aging‐related brain pathophysiology.

Our metagenomic and metabolomic results revealed microbial products of tryptophan, including IAA, indole‐3‐propinoic acid (IPA) and indolelactic acid (ILA), were significantly decreased with age, in which deficiency of ILA is associated with age‐dependent remodeling of gut microbiota‐host metabolome (Wu et al. 2021) and IPA has a neuroprotective impact in healthy elderly (Kim et al. 2023). IAA is previously known to be produced by *Bacteroides* and *Clostridial* species, while *Bifidobacterium* spp. has been reported to generate ILA (Roager and Licht 2018). In the present study, we uncovered that *B.p* indeed has the capability of converting Trp to IAA and elevating IAA bioavailability levels from the periphery to the brain. Of note, we cannot rule out that the host may mediate the process of *B.p*‐elevated IAA bioavailability in the brain since some endogenous production of IAA in mammalian tissues also occurs (Platten et al. 2019; Weissbach et al. 1959). NLRP3 has been documented to promote neuroinflammation by mediating proinflammatory factors (Yang et al. 2024; Su et al. 2024). IAA has been recently reported to penetrate the blood–brain barrier and alleviate cognitive impairment and AD‐like pathology by suppressing Nlrp3 transcription (Guo et al. 2023). It is partially consistent with our results that IAA dose‐dependently inhibits microglia activation. Beyond that, we observed additional impacts of IAA on weakening microglia engulfment of synapses in an AHR‐dependent manner. Recent studies identified that commensal bacteria, such as *Bacteroides thetaiotaomicron* , *Bacteroides eggerthii* , *Bifidobacterium adolescentis* , and *Clostridium bartlettii* , can metabolize tryptophan to IAA [PMID:30120222]. Other IAA‐producing bacteria, apart from *B.p*, may also be protective against aging‐related cognitive decline, which needs to be further investigated. Besides, there remains the possibility that other molecules apart from IAA derived from *B.p* have protective potential against aging‐related cognitive decline.

In summary, our current study revealed a new mechanistic role of aged gut microbiota in affecting cognitive decline and microglia‐mediated synapse loss, highlighting the potential of *B.p* and its produced IAA in preventing neurodegenerative diseases.

### 3.1 Limitations

The limitations of the current study were summarized as follows. First, the factors that are protective against aging‐related cognitive decline may be a complex multifactorial synergistic crosstalk process; whether other bacteria play roles during the aging process or work in concert with other bacteria to maintain cognitive function still requires further explanation. Secondly, IAA‐generating enzymes of *B.p* were yet to be identified. Thirdly, there remains the possibility that other molecules apart from IAA derived from *B.p* have protective functions against aging‐related cognitive decline. Further investigation is warranted to uncover the exact mechanism of *B.p* in improving aging‐related cognitive decline. Finally, the fecal *B.p* abundance and IAA need to be evaluated in large‐scale cohorts, which is of vital significance to translate current findings to the clinic. It should be noted that the conclusions drawn from our current study are based on laboratory FMT, which cannot be directly extrapolated to the real world. The actual interactions of the gut microbiota between the old and young people warrant further investigation.

## 4 Methods

### 4.1 Animal Experiments

The male C57BL/6J mice were all obtained from Shanghai Laboratory Animal Center (Shanghai, China). For aging mice, they were fed to 100 weeks old since 8 weeks old. The 5 × FAD mice were obtained from Wuhan Youdu Biological Co. LTD. All mice were housed in a constant 12 h dark and 12 h light cycle with a normal chow diet and water. All animal experiments were performed in accordance with the Guidelines for Animal Experiment of Shanghai University of Traditional Chinese Medicine and were approved by the institutional Animal Ethics Committee (PZSHUTCM2303160004, PZSHUTCM2308310003).

### 4.2 Human Samples

Twenty‐one patients with cognitive impairment (CI, 9 MCI patients, 12 ad patients) and 24 healthy controls (HC) were enrolled. The clinical samples were approved by the Ethics committee of Huashan Hospital, Fudan University (HIRB‐2022‐04). Fecal samples from healthy and elderly patients with cognitive impairment were collected from Huashan Hospital with informed consent.

Inclusion criteria: (1) ≥ 60 years old; (2) meet the National Institute on Aging and Alzheimer's Disease Association (NIA‐AA) diagnostic criteria for dementia likely to be caused by AD (2011); (3) MMSE score: illiterate ≤ 17, primary school ≤ 20, secondary school and above ≤ 24; (4) meet the diagnostic criteria of PET‐CT (including Aβ‐PET and Tau‐PET) or cerebrospinal fluid Aβ40‐42 and P‐Tau detection; (5) consistent with the fMRI diagnosis; (6) be able to cooperate with physicians for treatment evaluation as required; (7) participants and their legal representatives understood and signed the informed consent form.

Exclusion criteria: (1) dementia caused by systemic diseases or nervous system diseases, such as hypoxic–ischemic encephalopathy, vascular dementia, various encephalitis, brain trauma, poisoning, hypothyroidism, general paresis dementia, dementia with lewy bodies, frontotemporal dementia, parkinsonian dementia, and so forth; (2) patients diagnosed with major geriatric depression or other serious mental disorders; (3) previous history of psychotropic drug abuse or alcohol abuse that interfered with cognitive function assessment; (4) patients with infection or fever, serious primary diseases of the brain, heart, liver, kidney, lung, blood, and other systems; (5) family history of dementia; (6) patients with metabolic diseases such as underweight or overweight (BMI < 18.5 or BMI ≥ 28), fatty liver, arteriosclerosis, diabetes, hyperthyroidism, and hyperlipidemia; (7) IBD or irritable bowel syndrome with potential gastrointestinal infections, tumors, or polyps; (8) taking drugs for cognitive impairment or mental disorders; (9) taking antihypertensive, hypoglycemic, and lipid‐lowering drugs for the prevention and treatment of chronic diseases; (10) have taken or are taking antibiotics, antidiarrheal drugs, probiotics, any drugs targeting intestinal microecology, or functional foods in the past month; (11) have taken or are taking antacids such as PPI, gastrointestinal motility drugs, glucocorticoids, non‐steroidal anti‐inflammatory drugs, immunosuppressants, or chemotherapy drugs in the past 3 months; (12) have obvious abnormal dietary structure (e.g., vegetarians) and bad living habits (e.g., frequent drinking, smoking, insomnia, staying up late, constipation, circadian reversal).

### 4.3 Fecal Microbiota Transplantation

For recipients, mice were first treated with an antibiotics cocktail, consisting of 50 mg/kg vancomycin, 100 mg/kg neomycin, 100 mg/kg metronidazole, and 1 mg/kg amphotericin by oral gavage, together with ampicillin provided in drinking water (1 g/L) for 7 days. To prepare the FMT samples, 100 mg fresh feces were collected from old donor mice and put into clean tubes. The samples were resuspended in 1 mL sterile PBS, homogenized, and centrifuged for 1 min at 500 *g* to eliminate the precipitate. Subsequently, high‐speed centrifugation at 15,000 *g* for 5 min was performed, the supernatant was discarded, and the precipitate retained. An additional 1 mL of aseptic PBS was added to the precipitate and administered orally to recipient mice immediately (200 μL per mouse, daily). Young control mice and old donor mice were gavaged with sterile PBS daily at the same time.

### 4.4 *B.p* Cultivation and Treatment

*Bifidobacterium pseudolongum* (*B.p*) ( *Bifidobacterium pseudolongum* subsp. *pseudolongum* Mitsuoka, ATCC 25526) was grown at 37°C in modified PYG medium at Anaerobic Workstation (DefendorAMW1000). *B.p* was centrifuged, enriched, resuspended in phosphate buffer solution (PBS) and kept in −80°C with 30% glycerin.

### 4.5 Open Field Test

The open field test was performed in a 50 cm × 50 cm × 50 cm open field. The mice were moved to the experiment room for 2 h before starting to adapt to the environment. For the official test, the mice were placed in the field with their faces towards the wall and were allowed to explore the field freely for 5 min. The time and number of entries into the open central area were recorded. The number of entries into the open central area and the time spent in the central area divided by the total time were analyzed.

### 4.6 Novel Object Recognition

Two familiar objects were placed in the center of the box. Mice were allowed to explore for 10 min. After 4 h, one of the objects was replaced by a new object (novel object) and the mice were allowed to explore for another 10 min. The time spent exploring the familiar object and the novel object was recorded and analyzed. The recognition index of mice was calculated by: Time novel/(Time novel + Time familiar) × 100%.

### 4.7 Morris Water Maze

For the Morris water maze test, the experiment was performed in a water tank with 1.2 m depth. The water temperature was maintained at approximately 20°C. Before training, the platform was positioned 1 cm above the water surface in order to be visible to the mice. Throughout the training period, the platform was placed 1 cm below the water surface. Each mouse was placed at different positions around the border of the maze to find the platform. If the mouse reached the platform in 60 s, it was allowed to stay for 15 s. If not, it would be guided to the platform and stay for 15 s. The training was performed 4 times a day for 6 days. Then, 24 h after the last training, the mice were allowed to search freely for 60 s, and the time spent looking for the platform was recorded.

### 4.8 Proteomics

The 4D‐SmartDIA quantitative proteome was performed on mouse hippocampus. Samples were removed from −80°C, weighed into a liquid nitrogen pre‐cooled mortar, and fully ground to powder with liquid nitrogen. Each group of samples was added to the powder at 4 times the volume of lysis buffer (8 M urea, 1% protease inhibitor) and lysed by ultrasonication. The samples were centrifuged at 4°C, 13,400 *g* for 10 min to remove the cellular debris, and the supernatant was transferred to a new centrifuge tube, and the protein concentration was determined by utilizing the BCA kit. Peptides were solubilized with liquid chromatography mobile phase A and separated using an EASY‐nLC 1200 ultra‐high performance liquid chromatography system. The peptide fragments were separated by the ultra‐high‐performance liquid chromatography (UHPLC) system and then injected into an NSI ion source for ionization and then into the Orbitrap Exploris 480 mass spectrometry for analysis. The ion source voltage was set to 2300 V, the FAIMS compensation voltage (CV) was set to −45 V, and both the peptide parent ion and its secondary fragments were detected and analyzed using a high‐resolution Orbitrap. The primary MS scan range was set to 350–1400 *m/z* and the scan resolution was set to 60,000; the secondary MS scan range was set to a fixed starting point of 120 *m/z* and the secondary scan resolution was set to 15,000. The data acquisition mode used a data‐independent scanning (DIA) program, where peptide ions following multiple consecutive *m/z* windows after the primary scan enter the HCD collision cell using a 27% fragmentation energy for fragmentation and sequentially for secondary mass spectrometry analysis. To improve the effective utilization of the mass spectrum, the automatic gain control (AGC) was set to 1E6 and the maximum injection time was set to 22 ms.

### 4.9 Metagenomics

After completing the genomic DNA extraction, the extracted genomic DNA is detected by 1% agarose gel electrophoresis. The junction is ligated using the kit NEXTFLEX Rapid DNA‐Seq Kit, and the self‐associated fragments are removed by screening with magnetic beads. The library templates are enriched by PCR amplification, and the magnetic beads are recovered from the PCR products to obtain the final libraries. One end of the library molecule is complementary to the primer bases, and the template information is immobilized on the chip after a round of amplification. The other end of the immobilized molecule is randomly complementary to another primer nearby and is also immobilized, forming a “bridge” PCR amplification produces DNA clusters, and the DNA amplicon is linearized into a single strand. Modified DNA polymerase and dNTP with four fluorescent markers are added to synthesize one base per cycle. The surface of the reaction plate is scanned with a laser to read the type of nucleotide that was polymerized in the first reaction for each template sequence. Chemical cleavage of the “fluorescent group” and the “termination group” restores the 3′ end adhesion, and the polymerization of the second nucleotide continues. The fluorescence signals collected in each round are counted to determine the sequence of the template DNA fragment. (Kit: Illumina NovaSeq Reagent Kits).

### 4.10 Metabolomic Analysis

Metabolic profiling was performed by Metabo‐Profile Biotechnology (Shanghai) Co. Ltd. Briefly, after derivatization, the samples were transferred to a 96‐well plate with 10 mL of internal standards in each well. An ultraperformance liquid chromatography coupled to tandem mass spectrometry (UPLC–MS/MS) system (ACQUITYUPLC‐Xevo TQ‐S, Waters Corp., Milford, MA, USA) was used to quantitate all targeted metabolites. The raw data files generated by UPLC–MS/MS were processed using Mass Lynx software (v4.1, Waters, Milford, MA, USA) to perform peak integration, calibration, and quantitation.

QTRAP 6500^+^ LC–MS/MS targeted quantification of tryptophan and IAA in hippocampus, cecum contents, and serum was analyzed.

Hippocampus: 10 mg of hippocampus was added to 100 μL of 50% methanol–water and magnetic beads, shaken for 60 s, 3 times, centrifuged at 13,400 *g* for 5 min. Then, 100 μL of supernatant was pipetted into another tube, and 300 μL of methanol was added to precipitate proteins, vortexed and mixed well, and then left to stand for 5 min, and then pipetted all the supernatant into a new tube. After nitrogen blowing and drying, 40 μL of 50% methanol–water was reconstituted, vortexed and mixed, centrifuged at 13,400 *g* for 5 min and bottled.

Cecal contents: 50 mg of cecum content was accurately weighed, and 500 μL of 50% methanol–water and magnetic beads were added to 50 mg of cecum content, which was shaken for 60 s, 3 times, and centrifuged at 13,400 *g* for 5 min. Then, 200 μL of supernatant was aspirated to a separate EP tube, and 600 μL of methanol was added to precipitate proteins, and then vortexed to mix well and allowed to stand for 5 min; then the entire supernatant was aspirated to a new tube. After blow‐drying with nitrogen, 40 μL of 50% methanol–water was reconstituted, vortexed and mixed, and centrifuged at 13,400 *g* for 5 min before bottling.

Blood: 50 μL of blood was aspirated, 150 μL of methanol was added to precipitate protein, centrifuged at 13,400 *g* for 5 min, and the supernatant was aspirated and bottled.

### 4.11 Quantification of *B.p* in Fecal Sample

Fecal samples from humans or mice were immediately collected in a clean tube and frozen at −80°C until DNA extraction. 100 mg of fecal sample was weighed for DNA extraction. Fecal DNA was extracted and normalized using the fecal extraction kit from TianGen (TianGen, DP328, China). Subsequently, gene expression of *B. pseudolongum* in the fecal DNA was quantified using qRT–PCR. The forward primer 5′‐CCCTTTTTCCGGGTCCTGT‐3′ and reverse primer 5′‐ATCCGAACTGAGACCGGTT‐3′ were used for Real‐time PCR (qPCR). The absolute copy number of the *B*.*p* of each sample was then calculated.

### 4.12 Ex Vivo Microbiome Assay

One hundred mg of fecal samples preserved from human beings (*n* = 16, including 8 healthy controls and 8 CI patients) and young, old, FMT group mice (*n* = 3) were weighed. The samples were crushed for 1 min and briefly centrifuged to remove the bottom residue. The supernatant was centrifuged at 13,400 *g* for 3 min. The bacterial precipitate at the bottom was cultured using 5 mL of GB culture and cultured in an anaerobic chamber. The microbiota was cultured for 72 h before use. The culture system was adjusted to match the optical density (OD) value of the pre‐cultured *B.p*. Both were then added to a new GB culture at a ratio of 1:100. Samples were collected at 0, 3, 6, 12, 24, and 36 h for subsequent testing QTRAP 6500^+^ LC–MS/MS targeted quantification of tryptophan and IAA.

### 4.13 Quantification of Indole‐d_5_ Tryptophan and IAA

Live and dead *B.p* bacteria (pasteurized by heating at 70°C for 30 min) were gavaged and colonized in C57BL/6J mice for 10 days, and then indole‐d_5_ tryptophan solution (100 mg/kg) was administered to each mouse by gavage, and samples of serum, hippocampus, and cecum content were collected after 8 h. The samples were prepared in the same way as above.

### 4.14 Cell Culture and Treatment

Primary neuron cells were bought from Procell (CL0697). The cells were plated on L‐lysine‐coated coverslips at a density of 2.4 × 10^4^ cells and cultured for 7 days. At day 7, microglia were plated onto primary neurons in a 1:3 microglia‐to‐neuron ratio for 6 h. The LPS (100 ng/mL), IAA (50, 100, 200 μg/mL) and AHR antagonist (BAY218) were added for 24 h before immunostaining. Images were detected and captured by two‐photon confocal microscope (Nikon, A1). To generate 3D images of microglia and neuron, images were recorded as vertical z stacks and capture by two‐photon confocal microscope (Nikon, A1).

### 4.15 Statistical Analysis

All statistical tests were described in figure legends and were performed using Prism v8.0 (GraphPad Software, La Jolla, CA, USA). All values in the text and figures were expressed as mean ± SEM. Statistical significance was analyzed using one‐way ANOVA with the method of Benjamini, Krieger, and Yekutieli for multiple‐group comparison (*p < 0.05, **p < 0.01, ***p < 0.005). Mann–Whitney test was used for two‐group comparison for clinical data analysis and unpaired Student *t*‐test for two‐group comparison for animal data analysis (*p < 0.05, **p < 0.01, ***p < 0.005).

## Author Contributions

**Mingxiao Li:** writing‐original draft preparation, methodology, investigation, performing experiments, visualization. **Jiaoqi Ren:** clinical sample and patient information collection. **Yiyang Bao:** performing experiments, collection and analysis of data. **Wenjing Wei:** resources, methodology, analyzed data. **Xuefei Yu:** photographing and analysis of immunofluorescence. **Xiaofang He:** data analysis, visualization. **Mutalifu gulisima:** data analysis, visualization. **Lili Sheng:** study design, data analysis. **Ningning Zheng:** investigation and data analysis. **Jianbo Wan:** revised the manuscript. **Houguang Zhou:** investigation, project administration, pathological diagnosis. **Ling Zhao:** study design, data analysis, and manuscript writing. **Houkai Li:** project administration, organization of the studies, supervised the project, correction of manuscript, and revised the manuscript. Houkai Li was the lead contact of the study. All data were generated in‐house, and no paper mill was used. All authors agree to be accountable for all aspects of the research to ensure its integrity and accuracy.

## Conflicts of Interest

The authors declare no conflicts of interest.

## Supporting information


Appendix S1.

## Data Availability

Raw data of metagenomics and 16S rRNA sequencing have been deposited in the National Center for Biotechnology Information under accession code PRJNA1132729, PRJNA1132759. The mass spectrometry proteomics data have been deposited to the ProteomeXchange Consortium via the PRIDE partner repository with the dataset identifier PXD053778. Quantitative data that support the findings of this study are available within this article and its Supporting Information. Other data or materials that support the findings of this study are readily available from the corresponding author upon reasonable request.
