# Supplementary material for: Aged Gut Microbiota Contributes to Cognitive Impairment and Hippocampal Synapse Loss in Mice
Source: Aging Cell. 2025 Apr 12;24(7):e70064. doi: 10.1111/acel.70064 (PMC12266779; doi:10.1111/acel.70064)
Supplement: Supplementary file 1 — Appendix S1. [file ACEL-24-e70064-s001.docx]

**Aged gut microbiota contributes to cognitive impairment and hippocampal synapse loss in mice**

**Table of Contents:**

1. **Supplementary Materials and Methods**
   1. Immunoﬂuorescence
   2. 16S rRNA sequencing
   3. Nissl staining
   4. Real-time Quantitative PCR (RT-qPCR)
   5. PET imaging

1.6 *C.elegans* strains and culture conditions

1. **Supplementary Figures and Tables**
   1. Supplementary Fig.S1 Aged gut microbiota impairs hippocampal neuronal morphology and dysregulates hippocampal proteome and fecal metabolome profiles in young adult recipients
   2. Supplementary Fig.S2 Validation of the key proteins identified in the proteomics analysis
   3. Supplementary Fig.S3 *B.p* could metabolize tryptophan directly to produce IAA.
   4. Supplementary Fig.S4 The impacts of live or dead *B.p* on the CYP1a1 of mice and lifespan of *C. elegans*
   5. Supplementary Fig.S5 The effects of IAA on microglia activation and microglia-mediated synapse loss
   6. Supplementary Table.S1 Differentially expressed proteins in the hippocampus among young, FMT and old groups resulted from proteomic analysis
   7. Supplementary Table.S2 The changes of hippocampal proteins related to glial activation and engulfment of synapse elimination among young, FMT and old groups
   8. Supplementary Table.S3 The relationship between synapse/glia markers and cell engulfment-related proteins
   9. Supplementary Table.S4 Differential bacterial species between young, FMT and old groups resulted from metagenome sequencing analysis
   10. Supplementary Table.S5 Differential metabolites between young and old groups resulted from serum metabolomic analysis
   11. Supplementary Table.S6 Clinical characteristics of the subjects
   12. Supplementary Table.S7 The primers used in the current study
   13. Supplementary Table.S8 The antibodies used in the current study

**Supplementary Materials and Methods**

**Immunoﬂuorescence**

For brain histology, brain samples were fixed in 4% paraformaldehyde, and embedded in paraffin. 3 μm coronary sections were prepared. Slides were prepared by baking at 60°C for 1-2 h; xylene I, II, and III for 10 min each; then gradient alcohol: 100% for 5 min, twice; 95% for 5 min, once; 70% for 5 min, once; and washed with water twice, each time for 5 min. The AR buffer was heated and boiled, the slides were placed in the buffer, boiled, and then heated at medium/low heat for 15-20 min, and then cooled naturally at room temperature. The slides were washed with distilled water for 3 min, treated with 3% H_2_O_2_ water for 10-15 min, and washed twice with 1×TBST for 3 min each time. Water-blocking circles were drawn with an immunohistochemical pen, drops of sealing solution were added, and the slides were sealed in a wet box at room temperature for 10 min. The primary antibody was added dropwise(IBA-1, 1:6000, ab178846, abcam; SYP, 1:1200, ab32127, abcam; PSD95, 1:500, 20665-1-AP, Thermo Fisher; CD68, 1:1200, CST97778, Cell Signaling Technology), and the slides were washed with 1×TBST for 3 times, each time for 3 min. The slides were incubated with HRP-labeled secondary antibody for 10 min, 1×TBST for 3 min and 1×TBST for 5 min each. TSA working concentration solution 100-150 μL was added dropwise on the slide, incubated at room temperature for 10 min, washed 2 times with 1×TBST for 3 min each time, then the AR buffer was heated in microwave and boiled, put into the slide and boiled, then turned to medium/low heat, heated for 15 min, then cooled naturally at room temperature, and washed with distilled water for 3 min. After that, the slides were washed with distilled water for 3 min and 1×TBST for 3 min each, and then repeated once. The slices were directly sealed with DAPI-containing anti-quenching sealer, incubated in a wet box at room temperature for 3-5 min, washed with 1×TBST for 5 min, washed with distilled water for 3 min, sealed with a drop of fluorescent anti-quenching sealer, and the coverslips were fixed with nail polish. Finally, images were detected and acquired using a 3DHISTECH Pannoramic MIDI fluorescence scanner. We initially adjusted each channel individually to the same parameters and subsequently saved them in PNG format. Then, utilizing the Image J software, the fluorescence intensity were calculated based on the mean fluorescence and area. Following normalization against the control group, the fold change of density were obtained and subsequently plotted.

**16S rRNA sequencing**

Microbial genomic DNA was isolated from fecal samples using the PF Mag-Bind Stool DNA Kit (Omega Bio-tek, Georgia, USA) following the manufacturer's guidelines. The quality of the DNA and its concentration were assessed using 1.0% agarose gel electrophoresis and a NanoDrop® ND-2000 spectrophotometer (Thermo Scientific Inc., USA), which was subsequently stored at -80℃ until required for further use. The V3-V4 hypervariable region of the bacterial 16S rRNA gene was amplified using the primer pairs 338F (5'-ACTCCTACGGGAGGCAGCAG-3') and 806R (5'-GGACTACHVGGGTWTCTAAT-3') utilizing an ABI GeneAmp® 9700 PCR thermocycler (ABI, CA, USA) (Ma et al., 2023). The resultant tags were grouped into operational taxonomic units (OTUs) with 97% similarity using Research Software (version 11.0.667).

**Nissl staining**

Conventional dewaxing (xylene I, xylene II for 15 min each, then gradient alcohol dehydration: 100% I, 100% II, 95%, 90%, 80%, 70%, 50% for 5 min each). Distilled water 3 times, each 5 min and then placed in 60°C with 1% toluidine blue staining for 40 min. After washing the dye with distilled water, it was dehydrated in 70%, 80% and 95% and 100% ethanol respectively, and then transparent with xylene. Finally, the film was sealed with neutral gum.

**Real-time Quantitative PCR (RT-qPCR)**

The total RNA from animal tissues or cells was isolated using EZB reagent kit. Equal amounts of RNA were reverse-transcibed into cDNA using qRT-PCR mix from TianGen. The qPCR were performed in 20 μL volumes with the following thermal cycling program: 95°C for 3 min, 40 cycles of 95°C for 3 s and 60°C for 30 s. Relative mRNA levels were determined and normalization to 18S. The primers used are shown in Table S7.

**PET imaging**

The ^18^F-florbetapir and ^18^F-MK6240 PET scans were performed by using a PET/CT system (Biograph mCT Flow PET/CT, Siemens, Erlangen, Germany) at the PET center of Huashan hospital to evaluate the Aβ and Tau burden. Cerebral amyloid PET scans were carried out 50 min after the intravenous injection of 7.4 MBq/kg (0.2 mCi/kg) florbetapir and lasted for 20 min. PET images were reconstructed by means of filtered back projection (FBP) algorithm with corrections for decay, normalization, dead time, photon attenuation, scatter and random coincidences. PET images were then coregistered to the individual structural MRI and spatially normalized in the Montreal Neurological Institute (MNI) template. Standard uptake value ratios (SUVRs) in the cortical regions of interest (ROIs) consisting of frontal gyrus, lateral parietal gyrus, lateral temporal gyrus, medial temporal gyrus, posterior cingulate gyrus, and precuneus were calculated by using cerebellar crus as a reference. Global SUVR values were calculated by weighted averaging of these ROIs.

***C.elegans* strains and culture conditions**

The *C. elegans* strains N2 was obtained from the Caenorhabditis Genetics Center (CGC, University of Minnesota, Minneapolis, MN, USA). *C. elegans* were cultured on NGM medium with the *E. coli* OP50 at 20°C. L4 stage nematodes were synchronized and incubated onto NGM plates containing live and dead *B.p* and OP50. During the experiment, the nematodes treated with different strains were transferred to a new plate every two days to avoid the interference of progeny. The status of the nematodes was observed every day, the number of survival and death was recorded, and the growth status of the nematodes was observed. The death criterion of the nematodes was that the body did not bend or the head did not swing after being touched, and the body was stiff. The number of dead nematodes was recorded and the growth curve was made. On the 7 th day of intervention with different strains, the number of swallows and body bends of single nematodes were recorded under a microscope within 30 s.

**Supplementary Figures**

**
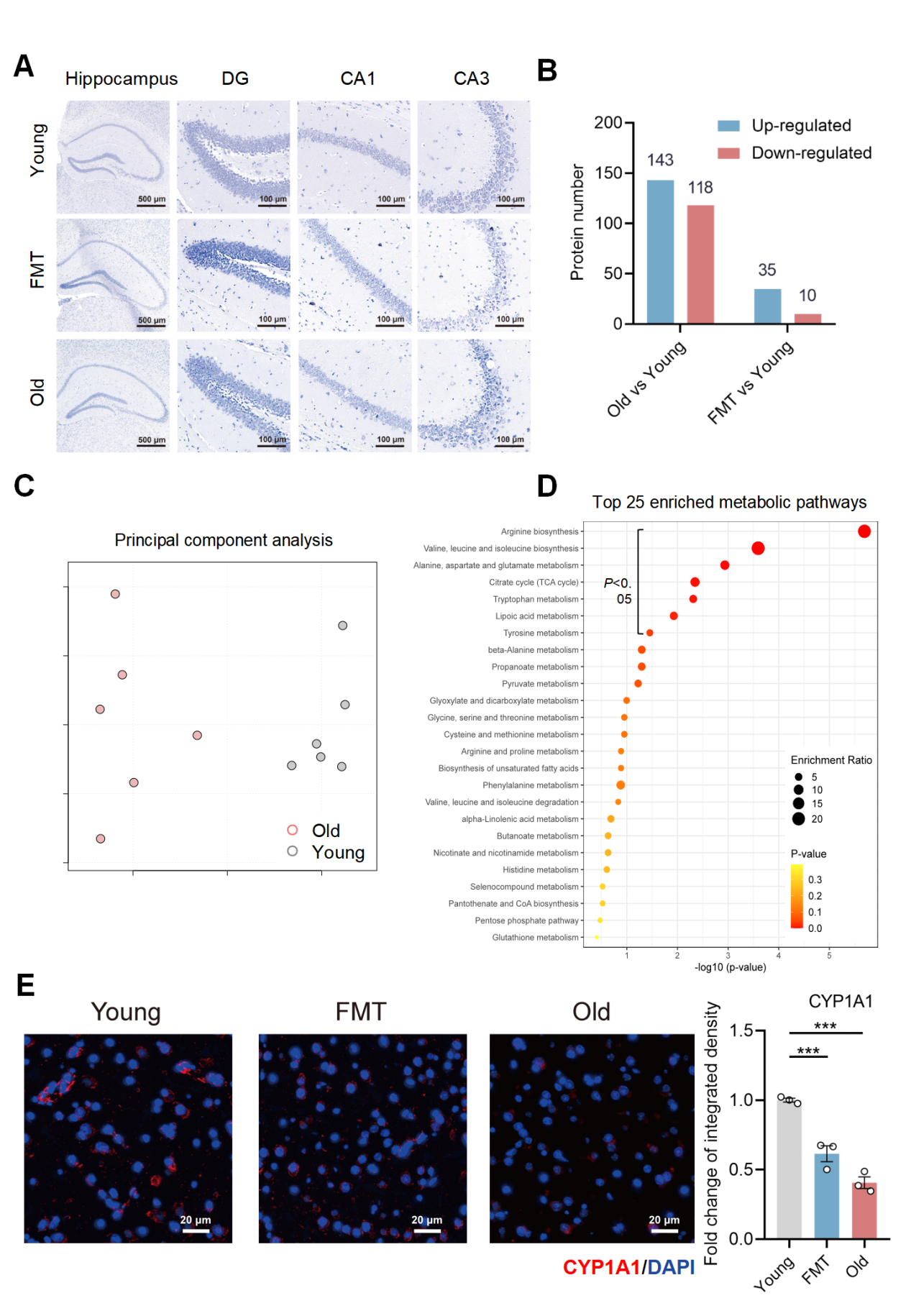
**

**Fig.S1 Aged gut microbiota impairs hippocampal neuronal morphology and dysregulates hippocampal proteome and serum metabolome profiles in young adult recipients**

(**A**) Nissl staining of tissual sections for hippocampal DG, CA1 and CA3 regions;

(**B**) The number of differential proteins between young and old groups or between young and FMT groups (n=3/group);

(**C**) Principal component analysis of serum metabolome profiles between young and old groups (n=6/ group);

(**D**) Bubble chart of Top 25 metabolic pathways of differential fecal metabolites resulted from KEGG-based enrichment analysis on MetaboAnalyst 6.0 (<https://www.metaboanalyst.ca/>);

(**E**) Representative immunofluorescence staining of CYP1A1 (red) and DAPI (blue) in the hippocampus of aging mice (scale bar: 20 μm).Statistical significance was analyzed using one-way ANOVA with the method of Benjamini, Krieger and Yekutieli for multiple-group comparison (***p<0.001).


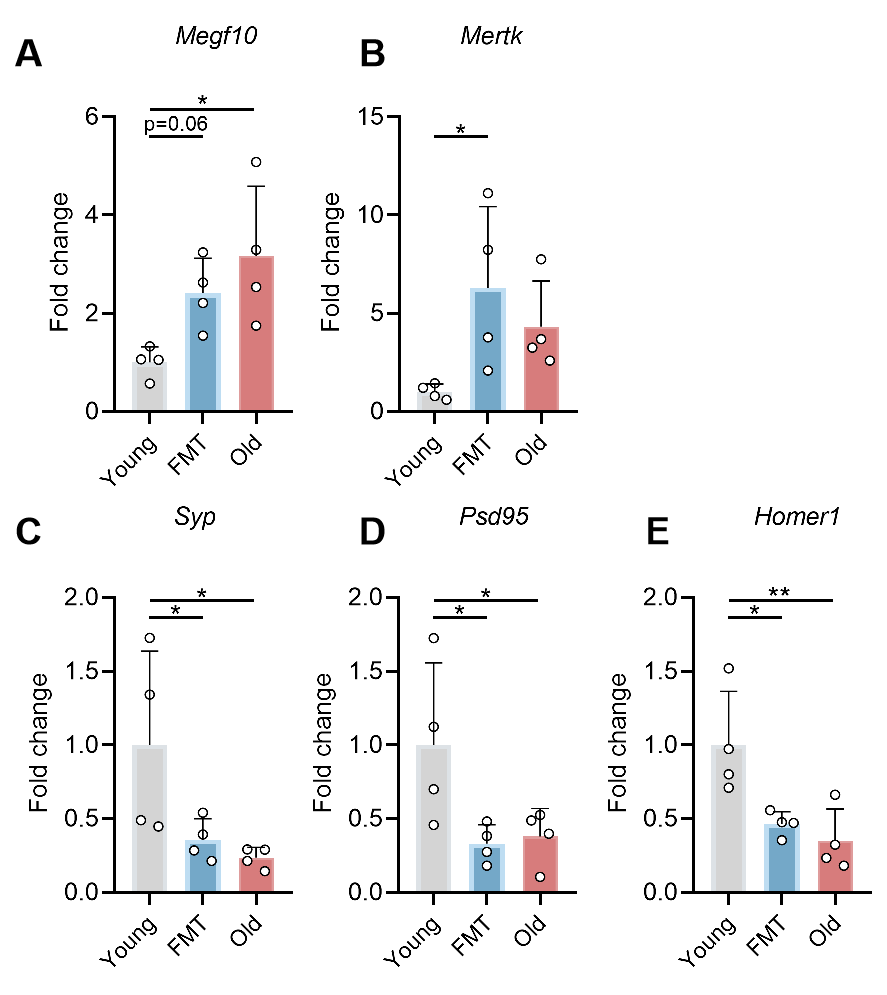


**Fig.S2 Validation of the key proteins identified in the proteomics analysis**

1. E) Relative mRNA expression of *Megf10, Mertk, Syp, Psd95, Homer1* in hippocampus of mice (n=4/group). Statistical significance was analyzed using one-way ANOVA with the method of Benjamini, Krieger and Yekutieli for multiple-group comparison (*p<0.05, ***p<0.001).


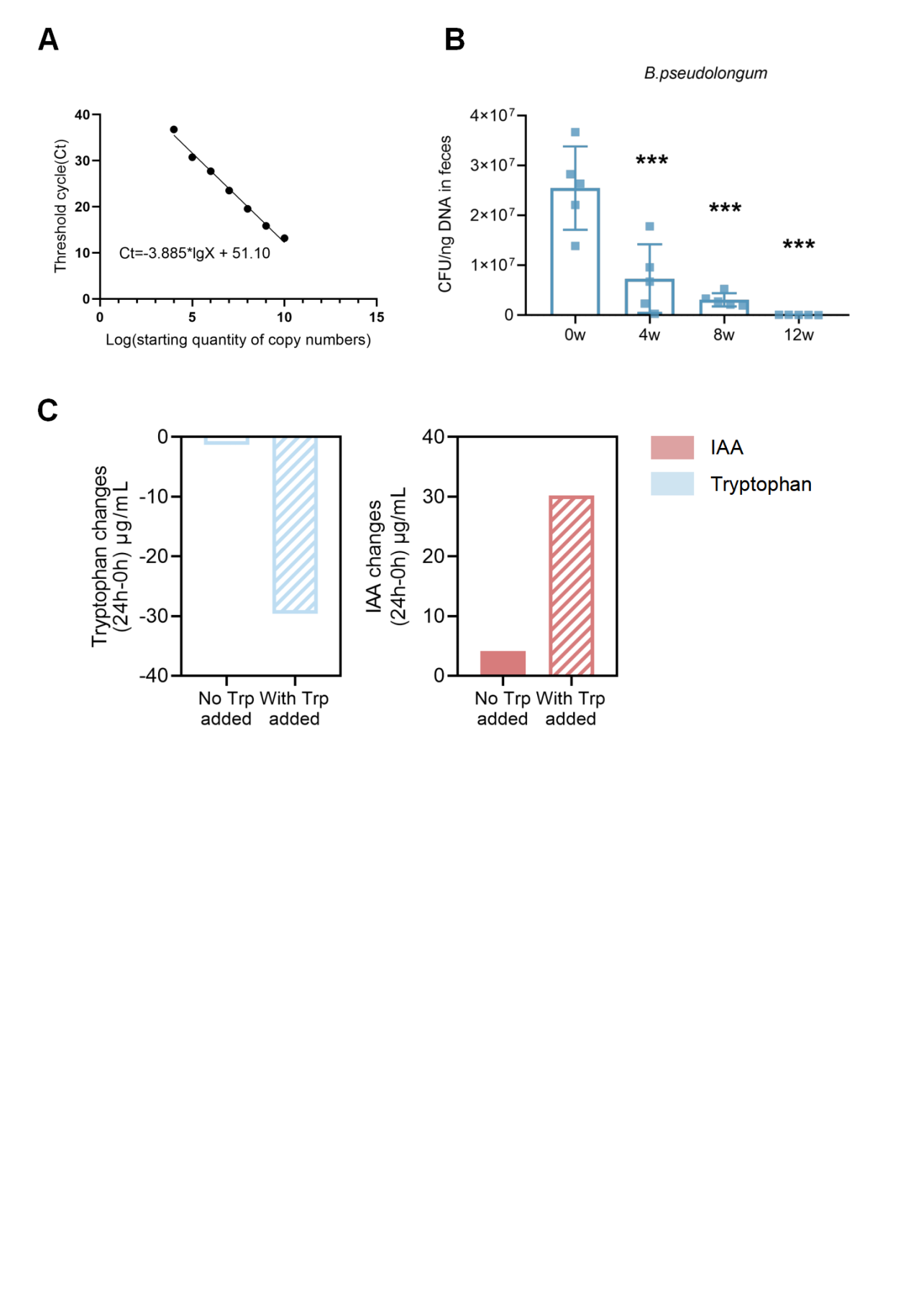


**Fig.S3 *B.p* could metabolize tryptophan directly to produce IAA.**

(**A**) Standard curve of *B.p* and Ct;

(**B**) Absolute quantification of *B.p* in feces of mice after FMT 0, 4, 8 and 12 weeks (n=5/group).

(**C**) The tryptophan and IAA level in the 24 hour cultural medium with or without tryptophan added (n=1/group). Statistical significance was analyzed using one-way ANOVA with the method of Benjamini, Krieger and Yekutieli for multiple-group comparison (***p<0.001).


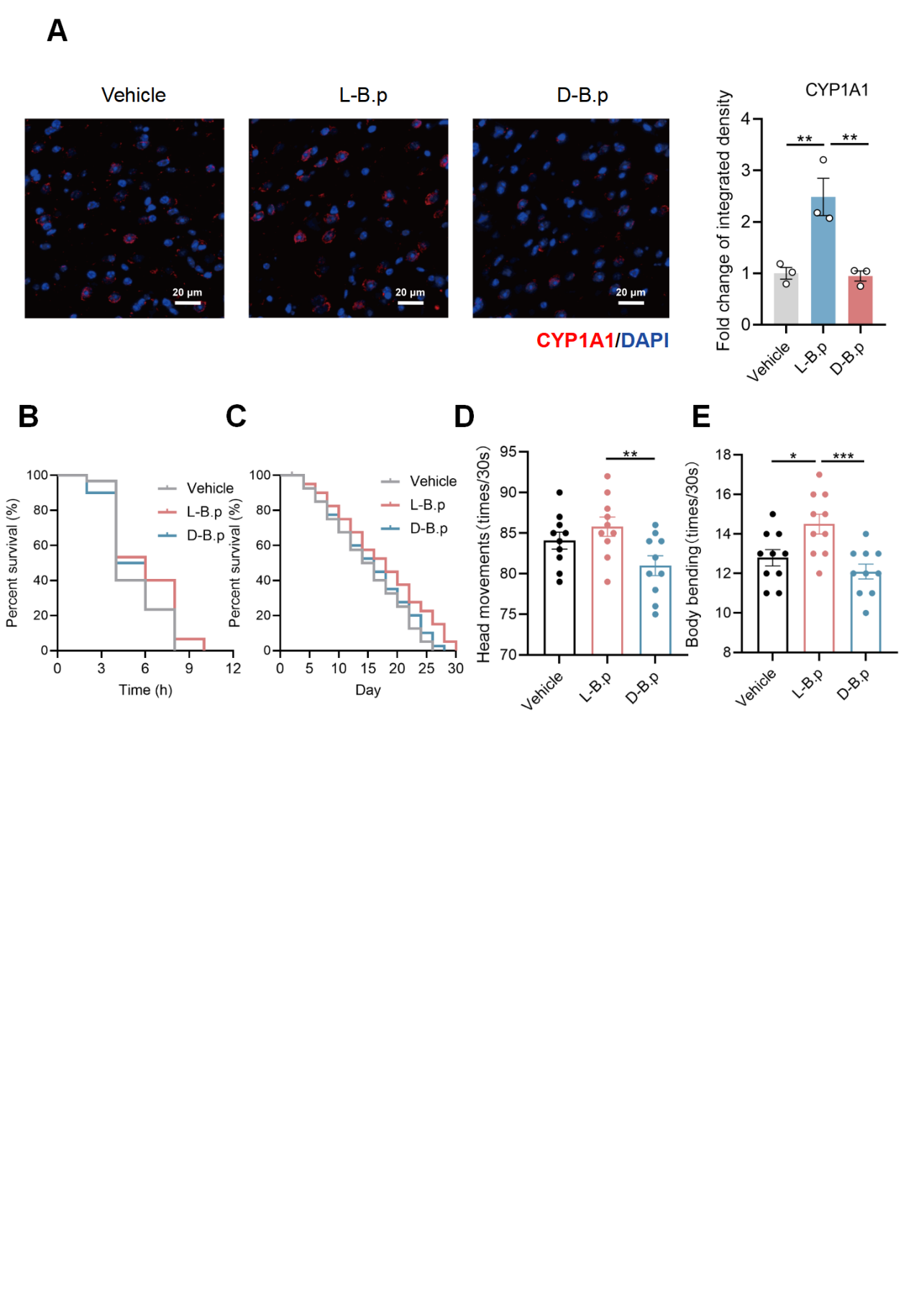


**Fig.S4 The impacts of live or dead *Bifidobacterium pseudolongum* on the CYP1A1 of mice and lifespan of *C. elegans***

(**A**) Representative immunofluorescence staining of CYP1A1 (red) and DAPI (blue) in the hippocampus of 5×FAD mice treated with live or dead *B.p* (scale bar: 20 μm).

(**B, C**)The lifespan of *C. elegans* cultured with *B.p* strains under a conventional or stressful condition (n=10/group);

(**D, E**) Effects of live or dead *B.p* strains on the head movements and body bending of *C. elegans* (n=10/group).


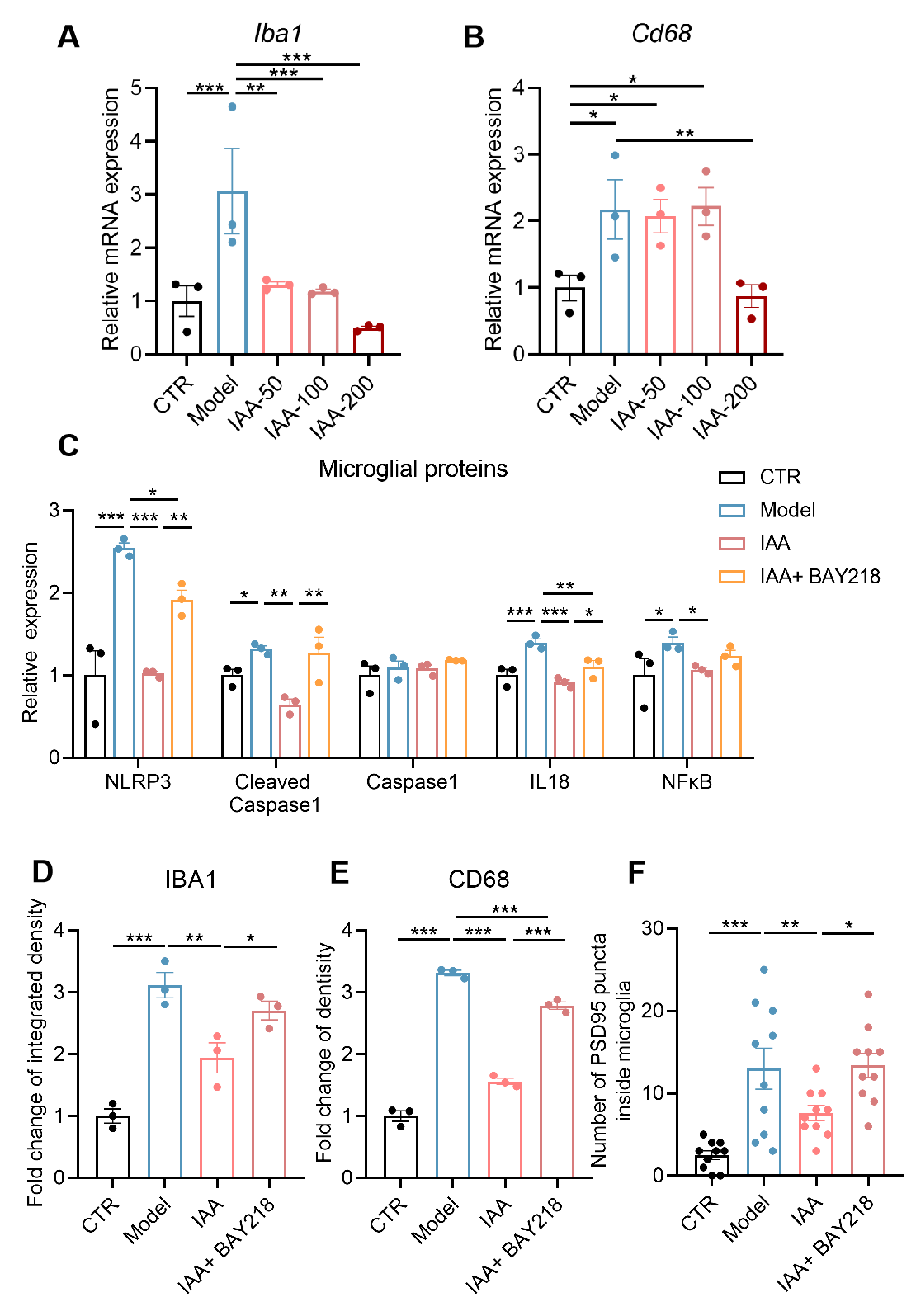


**Fig.S5 The effects of IAA on microglia activation and microglia-mediated synapse loss**

(**A, B**) The effects of IAA on mRNA expression of *Iba-1* and *Cd68* in microglia Bv-2 cells;

(**C**) The expressions of proteins associated with inflammation in Bv-2 microglia;

(**D, E**) Calculated results of IBA-1 and CD68 in the section of Bv-2 cell line;

(**F**) Number of PSD96 puncta inside microglia in neuron-microglia co-cultural sections.

n=3/group, Statistical significance was analyzed using one-way ANOVA with the method of Benjamini, Krieger and Yekutieli for multiple-group comparison (*p<0.05, **p<0.01, ***p<0.001).

| **Table S1 Differential hippocampal proteins between young, FMT and old groups resulted from proteomics sequencing analysis** | | | | | | | | | |
| --- | --- | --- | --- | --- | --- | --- | --- | --- | --- |
| **Protein accession** | **Protein description** | | **Gene name** | | | | **Old/Young Ratio** | | **Old/Young P value** |
| P58242 | Acid sphingomyelinase-like phosphodiesterase 3b OS=Mus musculus OX=10090 GN=Smpdl3b PE=1 SV=1 | | | Smpdl3b | | | 1.5793 | | 0.0001 |
| P22366 | Myeloid differentiation primary response protein MyD88 OS=Mus musculus OX=10090 GN=Myd88 PE=1 SV=3 | | | Myd88 | | | 1.5777 | | 0.0004 |
| Q80YX1 | Tenascin OS=Mus musculus OX=10090 GN=Tnc PE=1 SV=1 | | | Tnc | | | 0.6142 | | 0.0006 |
| Q9ESM3 | Hyaluronan and proteoglycan link protein 2 OS=Mus musculus OX=10090 GN=Hapln2 PE=2 SV=1 | | | Hapln2 | | | 3.4788 | | 0.0006 |
| Q499E5 | Storkhead-box protein 2 OS=Mus musculus OX=10090 GN=Stox2 PE=2 SV=2 | | | Stox2 | | | 0.6830 | | 0.0007 |
| E9Q6B2 | Coiled-coil domain-containing protein 85C OS=Mus musculus OX=10090 GN=Ccdc85c PE=1 SV=1 | | | Ccdc85c | | | 0.7199 | | 0.0010 |
| P0C7M9 | C-type lectin domain family 2 member L OS=Mus musculus OX=10090 GN=Clec2l PE=1 SV=1 | | | Clec2l | | | 1.2527 | | 0.0011 |
| O35409 | Glutamate carboxypeptidase 2 OS=Mus musculus OX=10090 GN=Folh1 PE=1 SV=2 | | | Folh1 | | | 1.5484 | | 0.0019 |
| Q9Z0M5 | Lysosomal acid lipase/cholesteryl ester hydrolase OS=Mus musculus OX=10090 GN=Lipa PE=1 SV=2 | | | Lipa | | | 1.2675 | | 0.0020 |
| Q8BG94 | COMM domain-containing protein 7 OS=Mus musculus OX=10090 GN=Commd7 PE=1 SV=1 | | | Commd7 | | | 1.5241 | | 0.0020 |
| Q69ZS6 | Synaptic vesicle glycoprotein 2C OS=Mus musculus OX=10090 GN=Sv2c PE=1 SV=2 | | | Sv2c | | | 1.2654 | | 0.0021 |
| Q03146 | Epithelial discoidin domain-containing receptor 1 OS=Mus musculus OX=10090 GN=Ddr1 PE=2 SV=2 | | | Ddr1 | | | 1.2841 | | 0.0022 |
| Q99K28 | ADP-ribosylation factor GTPase-activating protein 2 OS=Mus musculus OX=10090 GN=Arfgap2 PE=1 SV=1 | | | Arfgap2 | | | 0.8052 | | 0.0023 |
| Q8BGI5 | Peroxisome assembly protein 26 OS=Mus musculus OX=10090 GN=Pex26 PE=1 SV=2 | | | Pex26 | | | 1.2919 | | 0.0024 |
| P62071 | Ras-related protein R-Ras2 OS=Mus musculus OX=10090 GN=Rras2 PE=1 SV=1 | | | Rras2 | | | 0.6224 | | 0.0024 |
| Q9DAM7 | Transmembrane protein 263 OS=Mus musculus OX=10090 GN=Tmem263 PE=1 SV=1 | | | Tmem263 | | | 0.7745 | | 0.0024 |
| P08556 | GTPase NRas OS=Mus musculus OX=10090 GN=Nras PE=1 SV=1 | | | Nras | | | 0.7383 | | 0.0024 |
| P01029 | Complement C4-B OS=Mus musculus OX=10090 GN=C4b PE=1 SV=3 | | | C4b | | | 2.9234 | | 0.0024 |
| P03987 | Ig gamma-3 chain C region OS=Mus musculus OX=10090 PE=1 SV=2 | | | -- | | | 0.1679 | | 0.0026 |
| Q9R118 | Serine protease HTRA1 OS=Mus musculus OX=10090 GN=Htra1 PE=1 SV=2 | | | Htra1 | | | 2.2239 | | 0.0028 |
| Q0VG49 | Uncharacterized protein C15orf61 homolog OS=Mus musculus OX=10090 PE=2 SV=2 | | | -- | | | 1.3336 | | 0.0028 |
| Q9QX11 | Cytohesin-1 OS=Mus musculus OX=10090 GN=Cyth1 PE=1 SV=2 | | | Cyth1 | | | 0.7596 | | 0.0029 |
| Q8BMK4 | Cytoskeleton-associated protein 4 OS=Mus musculus OX=10090 GN=Ckap4 PE=1 SV=2 | | | Ckap4 | | | 0.7813 | | 0.0029 |
| Q3UVY5 | Pecanex-like protein 4 OS=Mus musculus OX=10090 GN=Pcnx4 PE=2 SV=2 | | | Pcnx4 | | | 1.3878 | | 0.0032 |
| Q8BLN5 | Lanosterol synthase OS=Mus musculus OX=10090 GN=Lss PE=1 SV=2 | | | Lss | | | 1.5052 | | 0.0035 |
| Q66JV4 | RNA-binding protein 12B-B OS=Mus musculus OX=10090 GN=Rbm12b2 PE=2 SV=2 | | | Rbm12b2 | | | 0.7786 | | 0.0036 |
| Q61107 | Guanylate-binding protein 4 OS=Mus musculus OX=10090 GN=Gbp4 PE=1 SV=1 | | | Gbp4 | | | 3.6195 | | 0.0039 |
| Q80T85 | DDB1- and CUL4-associated factor 5 OS=Mus musculus OX=10090 GN=Dcaf5 PE=1 SV=2 | | | Dcaf5 | | | 0.7792 | | 0.0041 |
| Q9QZC7 | Pleckstrin homology domain-containing family B member 2 OS=Mus musculus OX=10090 GN=Plekhb2 PE=1 SV=1 | | | Plekhb2 | | | 1.5463 | | 0.0041 |
| Q8CHY6 | Transcriptional repressor p66 alpha OS=Mus musculus OX=10090 GN=Gatad2a PE=1 SV=2 | | | Gatad2a | | | 1.3214 | | 0.0042 |
| Q61410 | cGMP-dependent protein kinase 2 OS=Mus musculus OX=10090 GN=Prkg2 PE=1 SV=1 | | | Prkg2 | | | 0.6885 | | 0.0043 |
| Q8VCS6 | Mediator of RNA polymerase II transcription subunit 9 OS=Mus musculus OX=10090 GN=Med9 PE=1 SV=1 | | | Med9 | | | 0.8196 | | 0.0044 |
| Q9WTZ1 | RING-box protein 2 OS=Mus musculus OX=10090 GN=Rnf7 PE=1 SV=1 | | | Rnf7 | | | 0.7981 | | 0.0045 |
| Q8VCQ3 | Nuclear receptor-binding factor 2 OS=Mus musculus OX=10090 GN=Nrbf2 PE=1 SV=1 | | | Nrbf2 | | | 0.8304 | | 0.0046 |
| P55264 | Adenosine kinase OS=Mus musculus OX=10090 GN=Adk PE=1 SV=2 | | | Adk | | | 1.3370 | | 0.0046 |
| Q61140 | Breast cancer anti-estrogen resistance protein 1 OS=Mus musculus OX=10090 GN=Bcar1 PE=1 SV=2 | | | Bcar1 | | | 1.2111 | | 0.0048 |
| Q6P9J5 | KN motif and ankyrin repeat domain-containing protein 4 OS=Mus musculus OX=10090 GN=Kank4 PE=1 SV=1 | | | Kank4 | | | 1.4247 | | 0.0049 |
| Q9JLQ0 | CD2-associated protein OS=Mus musculus OX=10090 GN=Cd2ap PE=1 SV=3 | | | Cd2ap | | | 1.3568 | | 0.0055 |
| Q8BU03 | Periodic tryptophan protein 2 homolog OS=Mus musculus OX=10090 GN=Pwp2 PE=1 SV=1 | | | Pwp2 | | | 0.7528 | | 0.0055 |
| Q9D513 | Meiosis-specific with OB domain-containing protein OS=Mus musculus OX=10090 GN=Meiob PE=1 SV=3 | | | Meiob | | | 1.3158 | | 0.0059 |
| P35980 | 60S ribosomal protein L18 OS=Mus musculus OX=10090 GN=Rpl18 PE=1 SV=3 | | | Rpl18 | | | 1.2436 | | 0.0059 |
| P14231 | Sodium/potassium-transporting ATPase subunit beta-2 OS=Mus musculus OX=10090 GN=Atp1b2 PE=1 SV=2 | | | Atp1b2 | | | 1.2431 | | 0.0059 |
| Q9D2D7 | Zinc finger protein 687 OS=Mus musculus OX=10090 GN=Znf687 PE=1 SV=1 | | | Znf687 | | | 1.8592 | | 0.0060 |
| Q8BY89 | Choline transporter-like protein 2 OS=Mus musculus OX=10090 GN=Slc44a2 PE=1 SV=2 | | | Slc44a2 | | | 1.2345 | | 0.0060 |
| Q61699 | Heat shock protein 105 kDa OS=Mus musculus OX=10090 GN=Hsph1 PE=1 SV=2 | | | Hsph1 | | | 0.8175 | | 0.0062 |
| P56212 | cAMP-regulated phosphoprotein 19 OS=Mus musculus OX=10090 GN=Arpp19 PE=1 SV=2 | | | Arpp19 | | | 0.6952 | | 0.0063 |
| Q8BP22 | CBY1-interacting BAR domain-containing protein 1 OS=Mus musculus OX=10090 GN=Cibar1 PE=1 SV=2 | | | Cibar1 | | | 0.7773 | | 0.0064 |
| Q64345 | Interferon-induced protein with tetratricopeptide repeats 3 OS=Mus musculus OX=10090 GN=Ifit3 PE=1 SV=1 | | | Ifit3 | | | 2.2891 | | 0.0064 |
| P16406 | Glutamyl aminopeptidase OS=Mus musculus OX=10090 GN=Enpep PE=1 SV=1 | | | Enpep | | | 0.7170 | | 0.0066 |
| Q9DC23 | DnaJ homolog subfamily C member 10 OS=Mus musculus OX=10090 GN=Dnajc10 PE=1 SV=2 | | | Dnajc10 | | | 1.2048 | | 0.0066 |
| Q921I9 | Exosome complex component RRP41 OS=Mus musculus OX=10090 GN=Exosc4 PE=1 SV=3 | | | Exosc4 | | | 1.2057 | | 0.0069 |
| Q60766 | Immunity-related GTPase family M protein 1 OS=Mus musculus OX=10090 GN=Irgm1 PE=1 SV=1 | | | Irgm1 | | | 1.2495 | | 0.0069 |
| Q3TB82 | Pleckstrin homology domain-containing family F member 1 OS=Mus musculus OX=10090 GN=Plekhf1 PE=2 SV=1 | | | Plekhf1 | | | 1.9619 | | 0.0071 |
| Q9CWU9 | Nucleoporin Nup37 OS=Mus musculus OX=10090 GN=Nup37 PE=1 SV=2 | | | Nup37 | | | 1.2623 | | 0.0072 |
| Q9EQK5 | Major vault protein OS=Mus musculus OX=10090 GN=Mvp PE=1 SV=4 | | | Mvp | | | 1.2786 | | 0.0074 |
| Q8K1S1 | Leucine-rich repeat LGI family member 4 OS=Mus musculus OX=10090 GN=Lgi4 PE=1 SV=1 | | | Lgi4 | | | 1.2544 | | 0.0076 |
| Q9CQG1 | Putative glutathione-specific gamma-glutamylcyclotransferase 2 OS=Mus musculus OX=10090 GN=Chac2 PE=1 SV=1 | | | Chac2 | | | 1.6737 | | 0.0076 |
| P62257 | Ubiquitin-conjugating enzyme E2 H OS=Mus musculus OX=10090 GN=Ube2h PE=1 SV=1 | | | Ube2h | | | 0.8263 | | 0.0078 |
| Q9JIK5 | Nucleolar RNA helicase 2 OS=Mus musculus OX=10090 GN=Ddx21 PE=1 SV=3 | | | Ddx21 | | | 1.2174 | | 0.0081 |
| Q8BTV1 | Tumor suppressor candidate 3 OS=Mus musculus OX=10090 GN=Tusc3 PE=1 SV=1 | | | Tusc3 | | | 0.7944 | | 0.0081 |
| Q9Z1Q5 | Chloride intracellular channel protein 1 OS=Mus musculus OX=10090 GN=Clic1 PE=1 SV=3 | | | Clic1 | | | 0.7135 | | 0.0088 |
| P79457 | Histone demethylase UTY OS=Mus musculus OX=10090 GN=Uty PE=1 SV=2 | | | Uty | | | 1.2648 | | 0.0088 |
| Q921S7 | 39S ribosomal protein L37, mitochondrial OS=Mus musculus OX=10090 GN=Mrpl37 PE=1 SV=1 | | | Mrpl37 | | | 1.2031 | | 0.0089 |
| Q8BU27 | Protein phosphatase 1M OS=Mus musculus OX=10090 GN=Ppm1m PE=2 SV=3 | | | Ppm1m | | | 1.3633 | | 0.0091 |
| P19157 | Glutathione S-transferase P 1 OS=Mus musculus OX=10090 GN=Gstp1 PE=1 SV=2 | | | Gstp1 | | | 1.4539 | | 0.0094 |
| Q99LH2 | Phosphatidylserine synthase 1 OS=Mus musculus OX=10090 GN=Ptdss1 PE=1 SV=1 | | | Ptdss1 | | | 0.7188 | | 0.0096 |
| Q80VM7 | Ankyrin repeat domain-containing protein 24 OS=Mus musculus OX=10090 GN=Ankrd24 PE=2 SV=4 | | | Ankrd24 | | | 1.3649 | | 0.0096 |
| P98086 | Complement C1q subcomponent subunit A OS=Mus musculus OX=10090 GN=C1qa PE=1 SV=2 | | | C1qa | | | 1.5004 | | 0.0097 |
| P28063 | Proteasome subunit beta type-8 OS=Mus musculus OX=10090 GN=Psmb8 PE=1 SV=2 | | | Psmb8 | | | 1.2580 | | 0.0098 |
| Q9WUA6 | RAC-gamma serine/threonine-protein kinase OS=Mus musculus OX=10090 GN=Akt3 PE=1 SV=1 | | | Akt3 | | | 0.7654 | | 0.0099 |
| P27659 | 60S ribosomal protein L3 OS=Mus musculus OX=10090 GN=Rpl3 PE=1 SV=3 | | | Rpl3 | | | 0.6880 | | 0.0099 |
| Q8BSQ9 | Protein polybromo-1 OS=Mus musculus OX=10090 GN=Pbrm1 PE=1 SV=4 | | | Pbrm1 | | | 0.7910 | | 0.0103 |
| Q9CXT7 | Transmembrane protein 192 OS=Mus musculus OX=10090 GN=Tmem192 PE=1 SV=1 | | | Tmem192 | | | 1.2676 | | 0.0103 |
| Q6GQT6 | Sterol regulatory element-binding protein cleavage-activating protein OS=Mus musculus OX=10090 GN=Scap PE=1 SV=1 | | | Scap | | | 1.2329 | | 0.0104 |
| O35114 | Lysosome membrane protein 2 OS=Mus musculus OX=10090 GN=Scarb2 PE=1 SV=3 | | | Scarb2 | | | 1.2313 | | 0.0104 |
| O70400 | PDZ and LIM domain protein 1 OS=Mus musculus OX=10090 GN=Pdlim1 PE=1 SV=4 | | | Pdlim1 | | | 1.3466 | | 0.0114 |
| Q3UZP4 | Small VCP/p97-interacting protein OS=Mus musculus OX=10090 GN=Svip PE=3 SV=1 | | | Svip | | | 1.2658 | | 0.0115 |
| D3Z7H4 | Germ cell-specific gene 1-like protein OS=Mus musculus OX=10090 GN=Gsg1l PE=1 SV=2 | | | Gsg1l | | | 1.4965 | | 0.0117 |
| Q9QYE9 | Pleckstrin homology domain-containing family B member 1 OS=Mus musculus OX=10090 GN=Plekhb1 PE=1 SV=1 | | | Plekhb1 | | | 1.9001 | | 0.0118 |
| Q99J85 | Neuronal pentraxin receptor OS=Mus musculus OX=10090 GN=Nptxr PE=1 SV=1 | | | Nptxr | | | 0.7440 | | 0.0119 |
| Q8BVZ5 | Interleukin-33 OS=Mus musculus OX=10090 GN=Il33 PE=1 SV=1 | | | Il33 | | | 1.6224 | | 0.0121 |
| Q8CFA2 | Aminomethyltransferase, mitochondrial OS=Mus musculus OX=10090 GN=Amt PE=1 SV=1 | | | Amt | | | 1.5755 | | 0.0124 |
| Q9JKC8 | AP-3 complex subunit mu-1 OS=Mus musculus OX=10090 GN=Ap3m1 PE=1 SV=1 | | | Ap3m1 | | | 0.8107 | | 0.0126 |
| Q9QUP5 | Hyaluronan and proteoglycan link protein 1 OS=Mus musculus OX=10090 GN=Hapln1 PE=1 SV=1 | | | Hapln1 | | | 1.4228 | | 0.0127 |
| Q60991 | Cytochrome P450 7B1 OS=Mus musculus OX=10090 GN=Cyp7b1 PE=1 SV=2 | | | Cyp7b1 | | | 0.7943 | | 0.0127 |
| P51910 | Apolipoprotein D OS=Mus musculus OX=10090 GN=Apod PE=1 SV=1 | | | Apod | | | 2.2109 | | 0.0128 |
| Q9Z1M0 | P2X purinoceptor 7 OS=Mus musculus OX=10090 GN=P2rx7 PE=1 SV=2 | | | P2rx7 | | | 1.2630 | | 0.0129 |
| O88531 | Palmitoyl-protein thioesterase 1 OS=Mus musculus OX=10090 GN=Ppt1 PE=1 SV=2 | | | Ppt1 | | | 1.5099 | | 0.0131 |
| Q9DB50 | AP-1 complex subunit sigma-2 OS=Mus musculus OX=10090 GN=Ap1s2 PE=1 SV=1 | | | Ap1s2 | | | 0.7523 | | 0.0133 |
| Q9CZS1 | Aldehyde dehydrogenase X, mitochondrial OS=Mus musculus OX=10090 GN=Aldh1b1 PE=1 SV=1 | | | Aldh1b1 | | | 0.8323 | | 0.0134 |
| Q9DA04 | Transmembrane protein 89 OS=Mus musculus OX=10090 GN=Tmem89 PE=2 SV=2 | | | Tmem89 | | | 1.2699 | | 0.0135 |
| P97449 | Aminopeptidase N OS=Mus musculus OX=10090 GN=Anpep PE=1 SV=4 | | | Anpep | | | 0.7447 | | 0.0135 |
| Q8VCW8 | Medium-chain acyl-CoA ligase ACSF2, mitochondrial OS=Mus musculus OX=10090 GN=Acsf2 PE=1 SV=1 | | | Acsf2 | | | 1.2277 | | 0.0136 |
| Q99L43 | Phosphatidate cytidylyltransferase 2 OS=Mus musculus OX=10090 GN=Cds2 PE=1 SV=1 | | | Cds2 | | | 1.2263 | | 0.0138 |
| Q99KK1 | Receptor expression-enhancing protein 3 OS=Mus musculus OX=10090 GN=Reep3 PE=1 SV=1 | | | Reep3 | | | 0.7769 | | 0.0140 |
| Q8K353 | Cysteine-rich and transmembrane domain-containing protein 1 OS=Mus musculus OX=10090 GN=Cystm1 PE=1 SV=1 | | | Cystm1 | | | 1.3783 | | 0.0148 |
| Q78TU8 | Actin-associated protein FAM107A OS=Mus musculus OX=10090 GN=Fam107a PE=1 SV=1 | | | Fam107a | | | 1.2406 | | 0.0150 |
| Q62188 | Dihydropyrimidinase-related protein 3 OS=Mus musculus OX=10090 GN=Dpysl3 PE=1 SV=1 | | | Dpysl3 | | | 0.7498 | | 0.0153 |
| Q64435 | UDP-glucuronosyltransferase 1-6 OS=Mus musculus OX=10090 GN=Ugt1a6 PE=1 SV=1 | | | Ugt1a6 | | | 1.4468 | | 0.0156 |
| Q9D8X1 | Copper homeostasis protein cutC homolog OS=Mus musculus OX=10090 GN=Cutc PE=1 SV=1 | | | Cutc | | | 1.2261 | | 0.0157 |
| P21661 | Neuroendocrine convertase 2 OS=Mus musculus OX=10090 GN=Pcsk2 PE=1 SV=1 | | | Pcsk2 | | | 1.3260 | | 0.0157 |
| P52196 | Thiosulfate sulfurtransferase OS=Mus musculus OX=10090 GN=Tst PE=1 SV=3 | | | Tst | | | 1.2224 | | 0.0159 |
| Q9D0U6 | Repressor of RNA polymerase III transcription MAF1 homolog OS=Mus musculus OX=10090 GN=Maf1 PE=2 SV=1 | | | Maf1 | | | 1.2665 | | 0.0161 |
| Q80V91 | Probable E3 ubiquitin-protein ligase DTX3 OS=Mus musculus OX=10090 GN=Dtx3 PE=1 SV=2 | | | Dtx3 | | | 1.2046 | | 0.0162 |
| Q3TYD4 | Arylsulfatase G OS=Mus musculus OX=10090 GN=Arsg PE=1 SV=1 | | | Arsg | | | 1.4886 | | 0.0169 |
| P62862 | 40S ribosomal protein S30 OS=Mus musculus OX=10090 GN=Fau PE=1 SV=1 | | | Fau | | | 0.6989 | | 0.0172 |
| Q6DIB5 | Multiple epidermal growth factor-like domains protein 10 OS=Mus musculus OX=10090 GN=Megf10 PE=1 SV=1 | | | Megf10 | | | 1.3279 | | 0.0182 |
| P49962 | Signal recognition particle 9 kDa protein OS=Mus musculus OX=10090 GN=Srp9 PE=1 SV=2 | | | Srp9 | | | 0.8042 | | 0.0182 |
| Q8BGB8 | Ubiquinone biosynthesis protein COQ4 homolog, mitochondrial OS=Mus musculus OX=10090 GN=Coq4 PE=1 SV=1 | | | Coq4 | | | 1.2588 | | 0.0189 |
| Q9DCU2 | Plasmolipin OS=Mus musculus OX=10090 GN=Pllp PE=1 SV=1 | | | Pllp | | | 1.6128 | | 0.0192 |
| Q3THK3 | General transcription factor IIF subunit 1 OS=Mus musculus OX=10090 GN=Gtf2f1 PE=1 SV=2 | | | Gtf2f1 | | | 0.7426 | | 0.0194 |
| P47962 | 60S ribosomal protein L5 OS=Mus musculus OX=10090 GN=Rpl5 PE=1 SV=3 | | | Rpl5 | | | 0.8258 | | 0.0195 |
| Q9D880 | Mitochondrial import inner membrane translocase subunit TIM50 OS=Mus musculus OX=10090 GN=Timm50 PE=1 SV=1 | | | Timm50 | | | 0.7631 | | 0.0195 |
| O08528 | Hexokinase-2 OS=Mus musculus OX=10090 GN=Hk2 PE=1 SV=1 | | | Hk2 | | | 1.4315 | | 0.0197 |
| P62823 | Ras-related protein Rab-3C OS=Mus musculus OX=10090 GN=Rab3c PE=1 SV=1 | | | Rab3c | | | 0.7214 | | 0.0198 |
| P14602 | Heat shock protein beta-1 OS=Mus musculus OX=10090 GN=Hspb1 PE=1 SV=3 | | | Hspb1 | | | 0.6176 | | 0.0198 |
| Q9WV03 | Protein FAM50A OS=Mus musculus OX=10090 GN=Fam50a PE=1 SV=1 | | | Fam50a | | | 1.8106 | | 0.0198 |
| Q8BYI8 | Protein FAM234B OS=Mus musculus OX=10090 GN=Fam234b PE=1 SV=1 | | | Fam234b | | | 1.2127 | | 0.0201 |
| Q8BHT6 | Beta-1,3-glucosyltransferase OS=Mus musculus OX=10090 GN=B3glct PE=1 SV=3 | | | B3glct | | | 0.6836 | | 0.0203 |
| Q80UW8 | DNA-directed RNA polymerases I, II, and III subunit RPABC1 OS=Mus musculus OX=10090 GN=Polr2e PE=1 SV=1 | | | Polr2e | | | 1.3395 | | 0.0204 |
| Q8VDZ4 | Palmitoyltransferase ZDHHC5 OS=Mus musculus OX=10090 GN=Zdhhc5 PE=1 SV=1 | | | Zdhhc5 | | | 0.6890 | | 0.0205 |
| Q8QZR5 | Alanine aminotransferase 1 OS=Mus musculus OX=10090 GN=Gpt PE=1 SV=3 | | | Gpt | | | 1.2099 | | 0.0206 |
| P61161 | Actin-related protein 2 OS=Mus musculus OX=10090 GN=Actr2 PE=1 SV=1 | | | Actr2 | | | 0.8161 | | 0.0207 |
| Q9D4F2 | Polyisoprenoid diphosphate/phosphate phosphohydrolase PLPP6 OS=Mus musculus OX=10090 GN=Plpp6 PE=1 SV=1 | | | Plpp6 | | | 0.7992 | | 0.0208 |
| Q9DAA6 | Exosome complex component CSL4 OS=Mus musculus OX=10090 GN=Exosc1 PE=1 SV=1 | | | Exosc1 | | | 1.2769 | | 0.0208 |
| Q8BJL0 | SWI/SNF-related matrix-associated actin-dependent regulator of chromatin subfamily A-like protein 1 OS=Mus musculus OX=10090 GN=Smarcal1 PE=1 SV=1 | | | Smarcal1 | | | 1.2940 | | 0.0211 |
| Q9CRD2 | ER membrane protein complex subunit 2 OS=Mus musculus OX=10090 GN=Emc2 PE=1 SV=1 | | | Emc2 | | | 0.8075 | | 0.0212 |
| Q60771 | Claudin-11 OS=Mus musculus OX=10090 GN=Cldn11 PE=1 SV=1 | | | Cldn11 | | | 1.6926 | | 0.0216 |
| P62245 | 40S ribosomal protein S15a OS=Mus musculus OX=10090 GN=Rps15a PE=1 SV=2 | | | Rps15a | | | 0.7974 | | 0.0216 |
| Q9CQU0 | Thioredoxin domain-containing protein 12 OS=Mus musculus OX=10090 GN=Txndc12 PE=1 SV=1 | | | Txndc12 | | | 0.7757 | | 0.0220 |
| P70689 | Gap junction beta-6 protein OS=Mus musculus OX=10090 GN=Gjb6 PE=1 SV=1 | | | Gjb6 | | | 1.3512 | | 0.0220 |
| P01942 | Hemoglobin subunit alpha OS=Mus musculus OX=10090 GN=Hba PE=1 SV=2 | | | Hba | | | 0.6310 | | 0.0224 |
| Q9WV86 | Katanin p60 ATPase-containing subunit A1 OS=Mus musculus OX=10090 GN=Katna1 PE=1 SV=1 | | | Katna1 | | | 1.2963 | | 0.0227 |
| Q9QXK7 | Cleavage and polyadenylation specificity factor subunit 3 OS=Mus musculus OX=10090 GN=Cpsf3 PE=1 SV=2 | | | Cpsf3 | | | 0.7413 | | 0.0229 |
| Q5U4E0 | LHFPL tetraspan subfamily member 4 protein OS=Mus musculus OX=10090 GN=Lhfpl4 PE=1 SV=1 | | | Lhfpl4 | | | 1.3763 | | 0.0229 |
| P35505 | Fumarylacetoacetase OS=Mus musculus OX=10090 GN=Fah PE=1 SV=2 | | | Fah | | | 1.2961 | | 0.0230 |
| Q99M28 | RNA-binding protein with serine-rich domain 1 OS=Mus musculus OX=10090 GN=Rnps1 PE=1 SV=1 | | | Rnps1 | | | 0.7230 | | 0.0231 |
| O35658 | Complement component 1 Q subcomponent-binding protein, mitochondrial OS=Mus musculus OX=10090 GN=C1qbp PE=1 SV=1 | | | C1qbp | | | 1.2094 | | 0.0232 |
| P02469 | Laminin subunit beta-1 OS=Mus musculus OX=10090 GN=Lamb1 PE=1 SV=3 | | | Lamb1 | | | 0.6916 | | 0.0241 |
| P42208 | Septin-2 OS=Mus musculus OX=10090 GN=Septin2 PE=1 SV=2 | | | Septin2 | | | 0.7493 | | 0.0245 |
| Q9Z2I8 | Succinate--CoA ligase [GDP-forming] subunit beta, mitochondrial OS=Mus musculus OX=10090 GN=Suclg2 PE=1 SV=3 | | | Suclg2 | | | 1.2155 | | 0.0246 |
| Q62059 | Versican core protein OS=Mus musculus OX=10090 GN=Vcan PE=1 SV=2 | | | Vcan | | | 1.2864 | | 0.0247 |
| P84099 | 60S ribosomal protein L19 OS=Mus musculus OX=10090 GN=Rpl19 PE=1 SV=1 | | | Rpl19 | | | 0.7032 | | 0.0251 |
| Q9WV76 | AP-4 complex subunit beta-1 OS=Mus musculus OX=10090 GN=Ap4b1 PE=1 SV=2 | | | Ap4b1 | | | 0.8003 | | 0.0252 |
| Q99ME2 | WD repeat-containing protein 6 OS=Mus musculus OX=10090 GN=Wdr6 PE=1 SV=1 | | | Wdr6 | | | 0.7949 | | 0.0254 |
| P98195 | Probable phospholipid-transporting ATPase IIB OS=Mus musculus OX=10090 GN=Atp9b PE=1 SV=4 | | | Atp9b | | | 0.7566 | | 0.0256 |
| Q7M6Z0 | Reticulon-4 receptor-like 2 OS=Mus musculus OX=10090 GN=Rtn4rl2 PE=1 SV=1 | | | Rtn4rl2 | | | 0.8212 | | 0.0259 |
| Q8CB44 | GRAM domain-containing protein 4 OS=Mus musculus OX=10090 GN=Gramd4 PE=1 SV=1 | | | Gramd4 | | | 0.8128 | | 0.0260 |
| Q6P4S8 | Integrator complex subunit 1 OS=Mus musculus OX=10090 GN=Ints1 PE=1 SV=2 | | | Ints1 | | | 0.7925 | | 0.0262 |
| Q9D8E6 | 60S ribosomal protein L4 OS=Mus musculus OX=10090 GN=Rpl4 PE=1 SV=3 | | | Rpl4 | | | 0.8004 | | 0.0266 |
| P62254 | Ubiquitin-conjugating enzyme E2 G1 OS=Mus musculus OX=10090 GN=Ube2g1 PE=1 SV=3 | | | Ube2g1 | | | 1.9247 | | 0.0267 |
| Q8R4V2 | Dual specificity protein phosphatase 15 OS=Mus musculus OX=10090 GN=Dusp15 PE=1 SV=3 | | | Dusp15 | | | 1.3205 | | 0.0268 |
| O08585 | Clathrin light chain A OS=Mus musculus OX=10090 GN=Clta PE=1 SV=2 | | | Clta | | | 0.6818 | | 0.0272 |
| Q8BHD8 | Protein-L-isoaspartate O-methyltransferase domain-containing protein 2 OS=Mus musculus OX=10090 GN=Pcmtd2 PE=2 SV=1 | | | Pcmtd2 | | | 1.4435 | | 0.0273 |
| Q9WU22 | Tyrosine-protein phosphatase non-receptor type 4 OS=Mus musculus OX=10090 GN=Ptpn4 PE=1 SV=2 | | | Ptpn4 | | | 1.4427 | | 0.0273 |
| P60041 | Somatostatin OS=Mus musculus OX=10090 GN=Sst PE=1 SV=1 | | | Sst | | | 0.4249 | | 0.0275 |
| Q3UN16 | Probable G-protein coupled receptor 162 OS=Mus musculus OX=10090 GN=Gpr162 PE=1 SV=2 | | | Gpr162 | | | 0.8144 | | 0.0277 |
| Q64310 | Surfeit locus protein 4 OS=Mus musculus OX=10090 GN=Surf4 PE=1 SV=1 | | | Surf4 | | | 0.8008 | | 0.0279 |
| Q8VCM3 | Zinc finger FYVE domain-containing protein 21 OS=Mus musculus OX=10090 GN=Zfyve21 PE=1 SV=2 | | | Zfyve21 | | | 1.4433 | | 0.0283 |
| Q9WTU0 | Lysine-specific demethylase PHF2 OS=Mus musculus OX=10090 GN=Phf2 PE=1 SV=2 | | | Phf2 | | | 0.7799 | | 0.0286 |
| Q91XI1 | tRNA-dihydrouridine(47) synthase [NAD(P)(+)]-like OS=Mus musculus OX=10090 GN=Dus3l PE=1 SV=1 | | | Dus3l | | | 0.6878 | | 0.0287 |
| P14106 | Complement C1q subcomponent subunit B OS=Mus musculus OX=10090 GN=C1qb PE=1 SV=2 | | | C1qb | | | 1.3638 | | 0.0287 |
| Q9WTX5 | S-phase kinase-associated protein 1 OS=Mus musculus OX=10090 GN=Skp1 PE=1 SV=3 | | | Skp1 | | | 1.2674 | | 0.0290 |
| Q64337 | Sequestosome-1 OS=Mus musculus OX=10090 GN=Sqstm1 PE=1 SV=1 | | | Sqstm1 | | | 1.3136 | | 0.0295 |
| Q6PD19 | Armadillo-like helical domain-containing protein 3 OS=Mus musculus OX=10090 GN=Armh3 PE=1 SV=2 | | | Armh3 | | | 1.5616 | | 0.0296 |
| O35943 | Frataxin, mitochondrial OS=Mus musculus OX=10090 GN=Fxn PE=1 SV=1 | | | Fxn | | | 0.7129 | | 0.0298 |
| Q80TN7 | Neuron navigator 3 OS=Mus musculus OX=10090 GN=Nav3 PE=1 SV=2 | | | Nav3 | | | 0.8294 | | 0.0298 |
| Q91WP6 | Serine protease inhibitor A3N OS=Mus musculus OX=10090 GN=Serpina3n PE=1 SV=1 | | | Serpina3n | | | 1.5146 | | 0.0299 |
| P98078 | Disabled homolog 2 OS=Mus musculus OX=10090 GN=Dab2 PE=1 SV=2 | | | Dab2 | | | 0.5705 | | 0.0300 |
| Q6P1D5 | Seizure 6-like protein OS=Mus musculus OX=10090 GN=Sez6l PE=1 SV=1 | | | Sez6l | | | 0.8119 | | 0.0303 |
| Q9EQX4 | Allograft inflammatory factor 1-like OS=Mus musculus OX=10090 GN=Aif1l PE=1 SV=1 | | | Aif1l | | | 1.3016 | | 0.0304 |
| Q8VCN9 | Tubulin-specific chaperone C OS=Mus musculus OX=10090 GN=Tbcc PE=1 SV=1 | | | Tbcc | | | 0.6043 | | 0.0308 |
| Q9R0Q6 | Actin-related protein 2/3 complex subunit 1A OS=Mus musculus OX=10090 GN=Arpc1a PE=1 SV=1 | | | Arpc1a | | | 0.7842 | | 0.0309 |
| Q9QX47 | Protein SON OS=Mus musculus OX=10090 GN=Son PE=1 SV=2 | | | Son | | | 0.8152 | | 0.0310 |
| P04370 | Myelin basic protein OS=Mus musculus OX=10090 GN=Mbp PE=1 SV=2 | | | Mbp | | | 1.8692 | | 0.0310 |
| P07759 | Serine protease inhibitor A3K OS=Mus musculus OX=10090 GN=Serpina3k PE=1 SV=2 | | | Serpina3k | | | 0.5175 | | 0.0315 |
| O88492 | Perilipin-4 OS=Mus musculus OX=10090 GN=Plin4 PE=1 SV=2 | | | Plin4 | | | 1.6028 | | 0.0320 |
| Q80Z24 | Neuronal growth regulator 1 OS=Mus musculus OX=10090 GN=Negr1 PE=1 SV=1 | | | Negr1 | | | 0.7369 | | 0.0322 |
| Q8BVY0 | Ribosomal L1 domain-containing protein 1 OS=Mus musculus OX=10090 GN=Rsl1d1 PE=1 SV=1 | | | Rsl1d1 | | | 0.7930 | | 0.0323 |
| Q8BG92 | Clavesin-2 OS=Mus musculus OX=10090 GN=Clvs2 PE=1 SV=1 | | | Clvs2 | | | 0.7891 | | 0.0325 |
| O54962 | Barrier-to-autointegration factor OS=Mus musculus OX=10090 GN=Banf1 PE=1 SV=1 | | | Banf1 | | | 1.5133 | | 0.0331 |
| Q9EPL9 | Peroxisomal acyl-coenzyme A oxidase 3 OS=Mus musculus OX=10090 GN=Acox3 PE=1 SV=2 | | | Acox3 | | | 1.2274 | | 0.0336 |
| Q8BZ60 | Stonin-2 OS=Mus musculus OX=10090 GN=Ston2 PE=1 SV=1 | | | Ston2 | | | 0.7810 | | 0.0338 |
| P70403 | Protein CASP OS=Mus musculus OX=10090 GN=Cux1 PE=1 SV=2 | | | Cux1 | | | 1.3105 | | 0.0339 |
| Q9JJ59 | ABC-type oligopeptide transporter ABCB9 OS=Mus musculus OX=10090 GN=Abcb9 PE=2 SV=1 | | | Abcb9 | | | 1.2277 | | 0.0340 |
| P02798 | Metallothionein-2 OS=Mus musculus OX=10090 GN=Mt2 PE=1 SV=2 | | | Mt2 | | | 3.2992 | | 0.0345 |
| Q8BTE0 | Succinate dehydrogenase assembly factor 4, mitochondrial OS=Mus musculus OX=10090 GN=Sdhaf4 PE=3 SV=2 | | | Sdhaf4 | | | 1.2004 | | 0.0346 |
| O35450 | FK506-binding protein-like OS=Mus musculus OX=10090 GN=Fkbpl PE=2 SV=1 | | | Fkbpl | | | 1.2394 | | 0.0351 |
| O55186 | CD59A glycoprotein OS=Mus musculus OX=10090 GN=Cd59a PE=2 SV=1 | | | Cd59a | | | 1.3634 | | 0.0356 |
| Q9WU79 | Proline dehydrogenase 1, mitochondrial OS=Mus musculus OX=10090 GN=Prodh PE=1 SV=2 | | | Prodh | | | 1.2129 | | 0.0361 |
| Q91X96 | Guanine nucleotide exchange factor MSS4 OS=Mus musculus OX=10090 GN=Rabif PE=1 SV=1 | | | Rabif | | | 0.7402 | | 0.0361 |
| P83882 | 60S ribosomal protein L36a OS=Mus musculus OX=10090 GN=Rpl36a PE=1 SV=2 | | | Rpl36a | | | 0.7029 | | 0.0364 |
| Q61070 | Etoposide-induced protein 2.4 OS=Mus musculus OX=10090 GN=Ei24 PE=1 SV=3 | | | Ei24 | | | 1.2730 | | 0.0364 |
| Q80XU3 | Nuclear ubiquitous casein and cyclin-dependent kinase substrate 1 OS=Mus musculus OX=10090 GN=Nucks1 PE=1 SV=1 | | | Nucks1 | | | 1.2578 | | 0.0366 |
| Q9D6H2 | Intraflagellar transport protein 25 homolog OS=Mus musculus OX=10090 GN=Hspb11 PE=1 SV=2 | | | Hspb11 | | | 0.6725 | | 0.0368 |
| P35290 | Ras-related protein Rab-24 OS=Mus musculus OX=10090 GN=Rab24 PE=1 SV=2 | | | Rab24 | | | 1.3176 | | 0.0369 |
| P43276 | Histone H1.5 OS=Mus musculus OX=10090 GN=H1-5 PE=1 SV=2 | | | H1-5 | | | 0.4162 | | 0.0370 |
| Q9QXL1 | Kinesin-like protein KIF21B OS=Mus musculus OX=10090 GN=Kif21b PE=1 SV=2 | | | Kif21b | | | 0.7806 | | 0.0376 |
| Q8C407 | Protein YIPF4 OS=Mus musculus OX=10090 GN=Yipf4 PE=1 SV=1 | | | Yipf4 | | | 1.2214 | | 0.0377 |
| P47963 | 60S ribosomal protein L13 OS=Mus musculus OX=10090 GN=Rpl13 PE=1 SV=3 | | | Rpl13 | | | 0.7474 | | 0.0377 |
| Q8C180 | Fibroblast growth factor receptor substrate 2 OS=Mus musculus OX=10090 GN=Frs2 PE=1 SV=3 | | | Frs2 | | | 0.8067 | | 0.0377 |
| Q9WTT4 | V-type proton ATPase subunit G 2 OS=Mus musculus OX=10090 GN=Atp6v1g2 PE=1 SV=1 | | | Atp6v1g2 | | | 0.7619 | | 0.0382 |
| Q9R020 | Zinc finger Ran-binding domain-containing protein 2 OS=Mus musculus OX=10090 GN=Zranb2 PE=1 SV=2 | | | Zranb2 | | | 1.3081 | | 0.0384 |
| Q0HA38 | Tetratricopeptide repeat protein 21B OS=Mus musculus OX=10090 GN=Ttc21b PE=1 SV=1 | | | Ttc21b | | | 1.2978 | | 0.0386 |
| P97478 | 5-demethoxyubiquinone hydroxylase, mitochondrial OS=Mus musculus OX=10090 GN=Coq7 PE=1 SV=3 | | | Coq7 | | | 0.8100 | | 0.0388 |
| P53996 | CCHC-type zinc finger nucleic acid binding protein OS=Mus musculus OX=10090 GN=Cnbp PE=1 SV=2 | | | Cnbp | | | 2.1121 | | 0.0388 |
| P00405 | Cytochrome c oxidase subunit 2 OS=Mus musculus OX=10090 GN=Mtco2 PE=1 SV=1 | | | Mtco2 | | | 1.6914 | | 0.0388 |
| Q80X85 | 28S ribosomal protein S7, mitochondrial OS=Mus musculus OX=10090 GN=Mrps7 PE=1 SV=1 | | | Mrps7 | | | 1.2831 | | 0.0393 |
| Q8R3V5 | Endophilin-B2 OS=Mus musculus OX=10090 GN=Sh3glb2 PE=1 SV=2 | | | Sh3glb2 | | | 0.7896 | | 0.0393 |
| P84089 | Enhancer of rudimentary homolog OS=Mus musculus OX=10090 GN=Erh PE=1 SV=1 | | | Erh | | | 0.6263 | | 0.0394 |
| Q3V1U8 | ELMO domain-containing protein 1 OS=Mus musculus OX=10090 GN=Elmod1 PE=1 SV=2 | | | Elmod1 | | | 0.7782 | | 0.0398 |
| Q8BNI4 | Derlin-2 OS=Mus musculus OX=10090 GN=Derl2 PE=1 SV=2 | | | Derl2 | | | 1.2833 | | 0.0400 |
| Q8R3L2 | Transcription factor 25 OS=Mus musculus OX=10090 GN=Tcf25 PE=1 SV=2 | | | Tcf25 | | | 0.6293 | | 0.0400 |
| P19536 | Cytochrome c oxidase subunit 5B, mitochondrial OS=Mus musculus OX=10090 GN=Cox5b PE=1 SV=1 | | | Cox5b | | | 1.2102 | | 0.0404 |
| P36993 | Protein phosphatase 1B OS=Mus musculus OX=10090 GN=Ppm1b PE=1 SV=1 | | | Ppm1b | | | 1.4823 | | 0.0405 |
| Q9D6K7 | Tetratricopeptide repeat protein 33 OS=Mus musculus OX=10090 GN=Ttc33 PE=1 SV=1 | | | Ttc33 | | | 1.2183 | | 0.0407 |
| P63037 | DnaJ homolog subfamily A member 1 OS=Mus musculus OX=10090 GN=Dnaja1 PE=1 SV=1 | | | Dnaja1 | | | 0.8251 | | 0.0409 |
| A2AB59 | Rho GTPase-activating protein 27 OS=Mus musculus OX=10090 GN=Arhgap27 PE=1 SV=1 | | | Arhgap27 | | | 1.2455 | | 0.0410 |
| Q7TPD2 | Protein FAM185A OS=Mus musculus OX=10090 GN=Fam185a PE=2 SV=1 | | | Fam185a | | | 1.2869 | | 0.0411 |
| Q3UHE1 | Membrane-associated phosphatidylinositol transfer protein 3 OS=Mus musculus OX=10090 GN=Pitpnm3 PE=1 SV=2 | | | Pitpnm3 | | | 1.3741 | | 0.0411 |
| P56376 | Acylphosphatase-1 OS=Mus musculus OX=10090 GN=Acyp1 PE=1 SV=2 | | | Acyp1 | | | 1.2859 | | 0.0415 |
| Q0VGB7 | Serine/threonine-protein phosphatase 4 regulatory subunit 2 OS=Mus musculus OX=10090 GN=Ppp4r2 PE=1 SV=1 | | | Ppp4r2 | | | 1.2354 | | 0.0416 |
| Q6DFW5 | Phospholipid-transporting ATPase IF OS=Mus musculus OX=10090 GN=Atp11b PE=1 SV=1 | | | Atp11b | | | 0.8310 | | 0.0420 |
| Q69ZN7 | Myoferlin OS=Mus musculus OX=10090 GN=Myof PE=1 SV=2 | | | Myof | | | 0.4819 | | 0.0421 |
| P63046 | Sulfotransferase 4A1 OS=Mus musculus OX=10090 GN=Sult4a1 PE=1 SV=1 | | | Sult4a1 | | | 1.2006 | | 0.0422 |
| A4FUP9 | Glycosyltransferase 1 domain-containing protein 1 OS=Mus musculus OX=10090 GN=Glt1d1 PE=2 SV=2 | | | Glt1d1 | | | 0.8216 | | 0.0422 |
| Q9D772 | Protein FAM219A OS=Mus musculus OX=10090 GN=Fam219a PE=1 SV=2 | | | Fam219a | | | 1.3160 | | 0.0423 |
| Q8BGB7 | Enolase-phosphatase E1 OS=Mus musculus OX=10090 GN=Enoph1 PE=1 SV=1 | | | Enoph1 | | | 0.7402 | | 0.0425 |
| O70572 | Sphingomyelin phosphodiesterase 2 OS=Mus musculus OX=10090 GN=Smpd2 PE=1 SV=1 | | | Smpd2 | | | 0.7191 | | 0.0429 |
| Q00897 | Alpha-1-antitrypsin 1-4 OS=Mus musculus OX=10090 GN=Serpina1d PE=1 SV=1 | | | Serpina1d | | | 0.5732 | | 0.0434 |
| Q8BML1 | [F-actin]-monooxygenase MICAL2 OS=Mus musculus OX=10090 GN=Mical2 PE=1 SV=2 | | | Mical2 | | | 0.7759 | | 0.0435 |
| Q9ERI2 | Ras-related protein Rab-27A OS=Mus musculus OX=10090 GN=Rab27a PE=1 SV=1 | | | Rab27a | | | 0.6985 | | 0.0440 |
| O89086 | RNA-binding protein 3 OS=Mus musculus OX=10090 GN=Rbm3 PE=1 SV=1 | | | Rbm3 | | | 1.3474 | | 0.0442 |
| Q9ERE8 | Talin rod domain-containing protein 1 OS=Mus musculus OX=10090 GN=Tlnrd1 PE=1 SV=1 | | | Tlnrd1 | | | 1.6256 | | 0.0443 |
| O54825 | Bystin OS=Mus musculus OX=10090 GN=Bysl PE=1 SV=3 | | | Bysl | | | 0.7675 | | 0.0444 |
| Q8BWU8 | Ethanolamine-phosphate phospho-lyase OS=Mus musculus OX=10090 GN=Etnppl PE=2 SV=1 | | | Etnppl | | | 1.6368 | | 0.0445 |
| Q6PDJ1 | VWFA and cache domain-containing protein 1 OS=Mus musculus OX=10090 GN=Cachd1 PE=2 SV=1 | | | Cachd1 | | | 1.3062 | | 0.0445 |
| Q99LI7 | Cleavage stimulation factor subunit 3 OS=Mus musculus OX=10090 GN=Cstf3 PE=1 SV=1 | | | Cstf3 | | | 1.6114 | | 0.0451 |
| Q9QYB1 | Chloride intracellular channel protein 4 OS=Mus musculus OX=10090 GN=Clic4 PE=1 SV=3 | | | Clic4 | | | 1.3667 | | 0.0451 |
| P61092 | E3 ubiquitin-protein ligase SIAH1A OS=Mus musculus OX=10090 GN=Siah1a PE=1 SV=1 | | | Siah1a | | | 2.2088 | | 0.0452 |
| Q80XU8 | Leucine-rich repeat and fibronectin type-III domain-containing protein 4 OS=Mus musculus OX=10090 GN=Lrfn4 PE=1 SV=1 | | | Lrfn4 | | | 0.7556 | | 0.0453 |
| Q63959 | Potassium voltage-gated channel subfamily C member 3 OS=Mus musculus OX=10090 GN=Kcnc3 PE=1 SV=3 | | | Kcnc3 | | | 1.2099 | | 0.0454 |
| Q8BWH0 | Sodium-coupled neutral amino acid transporter 7 OS=Mus musculus OX=10090 GN=Slc38a7 PE=1 SV=1 | | | Slc38a7 | | | 0.8002 | | 0.0455 |
| Q9JHE3 | Neutral ceramidase OS=Mus musculus OX=10090 GN=Asah2 PE=1 SV=1 | | | Asah2 | | | 1.2737 | | 0.0455 |
| Q91WK0 | Leucine-rich repeat flightless-interacting protein 2 OS=Mus musculus OX=10090 GN=Lrrfip2 PE=1 SV=1 | | | Lrrfip2 | | | 2.6684 | | 0.0457 |
| Q80UP5 | Ankyrin repeat domain-containing protein 13A OS=Mus musculus OX=10090 GN=Ankrd13a PE=1 SV=2 | | | Ankrd13a | | | 1.6122 | | 0.0457 |
| P10833 | Ras-related protein R-Ras OS=Mus musculus OX=10090 GN=Rras PE=1 SV=1 | | | Rras | | | 0.8273 | | 0.0460 |
| P51881 | ADP/ATP translocase 2 OS=Mus musculus OX=10090 GN=Slc25a5 PE=1 SV=3 | | | Slc25a5 | | | 0.8211 | | 0.0463 |
| Q80XP9 | Serine/threonine-protein kinase WNK3 OS=Mus musculus OX=10090 GN=Wnk3 PE=1 SV=3 | | | Wnk3 | | | 0.6307 | | 0.0465 |
| A2A9Q0 | Fibronectin type III domain-containing protein 10 OS=Mus musculus OX=10090 GN=Fndc10 PE=2 SV=1 | | | Fndc10 | | | 1.3481 | | 0.0467 |
| O09174 | Alpha-methylacyl-CoA racemase OS=Mus musculus OX=10090 GN=Amacr PE=1 SV=4 | | | Amacr | | | 1.2817 | | 0.0468 |
| Q8CHH9 | Septin-8 OS=Mus musculus OX=10090 GN=Septin8 PE=1 SV=4 | | | Septin8 | | | 0.8082 | | 0.0473 |
| Q99J23 | GH3 domain-containing protein OS=Mus musculus OX=10090 GN=Ghdc PE=2 SV=2 | | | Ghdc | | | 0.7813 | | 0.0476 |
| P99027 | 60S acidic ribosomal protein P2 OS=Mus musculus OX=10090 GN=Rplp2 PE=1 SV=3 | | | Rplp2 | | | 0.7943 | | 0.0483 |
| P97461 | 40S ribosomal protein S5 OS=Mus musculus OX=10090 GN=Rps5 PE=1 SV=3 | | | Rps5 | | | 0.6479 | | 0.0483 |
| P29699 | Alpha-2-HS-glycoprotein OS=Mus musculus OX=10090 GN=Ahsg PE=1 SV=1 | | | Ahsg | | | 0.6758 | | 0.0485 |
| Q9JHU2 | Palmdelphin OS=Mus musculus OX=10090 GN=Palmd PE=1 SV=1 | | | Palmd | | | 0.8097 | | 0.0492 |
| Q9D819 | Inorganic pyrophosphatase OS=Mus musculus OX=10090 GN=Ppa1 PE=1 SV=1 | | | Ppa1 | | | 0.8074 | | 0.0494 |
| Q99JY9 | Actin-related protein 3 OS=Mus musculus OX=10090 GN=Actr3 PE=1 SV=3 | | | Actr3 | | | 0.8087 | | 0.0497 |
| Q8CFR0 | Complement C1q-like protein 2 OS=Mus musculus OX=10090 GN=C1ql2 PE=1 SV=1 | | | C1ql2 | | | 1.2836 | | 0.0498 |
| Q8CH77 | Neuron navigator 1 OS=Mus musculus OX=10090 GN=Nav1 PE=1 SV=2 | | | Nav1 | | | 0.8197 | | 0.0499 |
| **Protein accession** | **Protein description** | **Gene name** | | | **FMT/Young Ratio** | | | **FMT/Young P value** | |
| Q0VG49 | Uncharacterized protein C15orf61 homolog OS=Mus musculus OX=10090 PE=2 SV=2 | | | -- | | 1.2861 | | 0.0029 | |
| Q07243 | Metal regulatory transcription factor 1 OS=Mus musculus OX=10090 GN=Mtf1 PE=1 SV=2 | | | Mtf1 | | 1.7487 | | 0.0042 | |
| Q3TGW2 | Endonuclease/exonuclease/phosphatase family domain-containing protein 1 OS=Mus musculus OX=10090 GN=Eepd1 PE=1 SV=1 | | | Eepd1 | | 0.7347 | | 0.0078 | |
| P56394 | Cytochrome c oxidase copper chaperone OS=Mus musculus OX=10090 GN=Cox17 PE=1 SV=2 | | | Cox17 | | 0.8141 | | 0.0088 | |
| Q6GQT6 | Sterol regulatory element-binding protein cleavage-activating protein OS=Mus musculus OX=10090 GN=Scap PE=1 SV=1 | | | Scap | | 1.2343 | | 0.0096 | |
| Q62422 | Osteoclast-stimulating factor 1 OS=Mus musculus OX=10090 GN=Ostf1 PE=1 SV=2 | | | Ostf1 | | 0.5296 | | 0.0107 | |
| Q9CXG3 | Peptidyl-prolyl cis-trans isomerase-like 4 OS=Mus musculus OX=10090 GN=Ppil4 PE=1 SV=2 | | | Ppil4 | | 1.2023 | | 0.0112 | |
| Q9DBY5 | Chromobox protein homolog 6 OS=Mus musculus OX=10090 GN=Cbx6 PE=1 SV=2 | | | Cbx6 | | 1.3255 | | 0.0114 | |
| P09926 | Surfeit locus protein 2 OS=Mus musculus OX=10090 GN=Surf2 PE=1 SV=1 | | | Surf2 | | 1.3035 | | 0.0127 | |
| Q91WG2 | Rab GTPase-binding effector protein 2 OS=Mus musculus OX=10090 GN=Rabep2 PE=1 SV=3 | | | Rabep2 | | 1.2015 | | 0.0135 | |
| Q80VM7 | Ankyrin repeat domain-containing protein 24 OS=Mus musculus OX=10090 GN=Ankrd24 PE=2 SV=4 | | | Ankrd24 | | 1.2251 | | 0.0144 | |
| Q7M753 | Pantothenate kinase 2, mitochondrial OS=Mus musculus OX=10090 GN=Pank2 PE=1 SV=1 | | | Pank2 | | 1.3168 | | 0.0152 | |
| Q9CYP7 | Sestrin-3 OS=Mus musculus OX=10090 GN=Sesn3 PE=1 SV=1 | | | Sesn3 | | 1.2617 | | 0.0159 | |
| Q60841 | Reelin OS=Mus musculus OX=10090 GN=Reln PE=1 SV=3 | | | Reln | | 1.2013 | | 0.0160 | |
| P70365 | Nuclear receptor coactivator 1 OS=Mus musculus OX=10090 GN=Ncoa1 PE=1 SV=2 | | | Ncoa1 | | 1.2016 | | 0.0190 | |
| P97868 | E3 ubiquitin-protein ligase RBBP6 OS=Mus musculus OX=10090 GN=Rbbp6 PE=1 SV=5 | | | Rbbp6 | | 1.6735 | | 0.0245 | |
| P45700 | Mannosyl-oligosaccharide 1,2-alpha-mannosidase IA OS=Mus musculus OX=10090 GN=Man1a1 PE=1 SV=1 | | | Man1a1 | | 1.5856 | | 0.0248 | |
| P22366 | Myeloid differentiation primary response protein MyD88 OS=Mus musculus OX=10090 GN=Myd88 PE=1 SV=3 | | | Myd88 | | 0.7540 | | 0.0256 | |
| P63011 | Ras-related protein Rab-3A OS=Mus musculus OX=10090 GN=Rab3a PE=1 SV=1 | | | Rab3a | | 0.6412 | | 0.0257 | |
| Q69ZQ1 | Myogenesis-regulating glycosidase OS=Mus musculus OX=10090 GN=Myorg PE=1 SV=2 | | | Myorg | | 1.2167 | | 0.0260 | |
| Q9CWU9 | Nucleoporin Nup37 OS=Mus musculus OX=10090 GN=Nup37 PE=1 SV=2 | | | Nup37 | | 1.2248 | | 0.0274 | |
| Q9JK92 | Heat shock protein beta-8 OS=Mus musculus OX=10090 GN=Hspb8 PE=1 SV=1 | | | Hspb8 | | 1.2107 | | 0.0285 | |
| Q9CQE7 | Endoplasmic reticulum-Golgi intermediate compartment protein 3 OS=Mus musculus OX=10090 GN=Ergic3 PE=1 SV=1 | | | Ergic3 | | 1.2187 | | 0.0288 | |
| P52624 | Uridine phosphorylase 1 OS=Mus musculus OX=10090 GN=Upp1 PE=1 SV=2 | | | Upp1 | | 1.2343 | | 0.0293 | |
| Q9JJV2 | Profilin-2 OS=Mus musculus OX=10090 GN=Pfn2 PE=1 SV=3 | | | Pfn2 | | 1.2875 | | 0.0296 | |
| Q9D2E2 | Target of EGR1 protein 1 OS=Mus musculus OX=10090 GN=Toe1 PE=1 SV=1 | | | Toe1 | | 1.5653 | | 0.0316 | |
| Q8BU27 | Protein phosphatase 1M OS=Mus musculus OX=10090 GN=Ppm1m PE=2 SV=3 | | | Ppm1m | | 1.3502 | | 0.0322 | |
| O88796 | Ribonuclease P protein subunit p30 OS=Mus musculus OX=10090 GN=Rpp30 PE=1 SV=1 | | | Rpp30 | | 1.4524 | | 0.0333 | |
| Q9ESP1 | Stromal cell-derived factor 2-like protein 1 OS=Mus musculus OX=10090 GN=Sdf2l1 PE=1 SV=2 | | | Sdf2l1 | | 1.2435 | | 0.0341 | |
| Q8K4Q8 | Collectin-12 OS=Mus musculus OX=10090 GN=Colec12 PE=1 SV=1 | | | Colec12 | | 1.2414 | | 0.0342 | |
| Q61624 | Zinc finger protein 148 OS=Mus musculus OX=10090 GN=Znf148 PE=1 SV=2 | | | Znf148 | | 1.2215 | | 0.0350 | |
| Q04899 | Cyclin-dependent kinase 18 OS=Mus musculus OX=10090 GN=Cdk18 PE=1 SV=1 | | | Cdk18 | | 1.2801 | | 0.0361 | |
| Q60963 | Platelet-activating factor acetylhydrolase OS=Mus musculus OX=10090 GN=Pla2g7 PE=1 SV=2 | | | Pla2g7 | | 1.2832 | | 0.0364 | |
| Q3TMX7 | Sulfhydryl oxidase 2 OS=Mus musculus OX=10090 GN=Qsox2 PE=1 SV=1 | | | Qsox2 | | 1.7146 | | 0.0372 | |
| Q5SVQ0 | Histone acetyltransferase KAT7 OS=Mus musculus OX=10090 GN=Kat7 PE=1 SV=1 | | | Kat7 | | 1.2305 | | 0.0373 | |
| Q99LJ6 | Glutathione peroxidase 7 OS=Mus musculus OX=10090 GN=Gpx7 PE=1 SV=1 | | | Gpx7 | | 0.6979 | | 0.0384 | |
| Q8BH79 | Anoctamin-10 OS=Mus musculus OX=10090 GN=Ano10 PE=1 SV=1 | | | Ano10 | | 1.2328 | | 0.0390 | |
| Q8VDT9 | 39S ribosomal protein L50, mitochondrial OS=Mus musculus OX=10090 GN=Mrpl50 PE=1 SV=2 | | | Mrpl50 | | 0.7190 | | 0.0394 | |
| Q80XL6 | Acyl-CoA dehydrogenase family member 11 OS=Mus musculus OX=10090 GN=Acad11 PE=1 SV=2 | | | Acad11 | | 1.2293 | | 0.0423 | |
| Q9WV91 | Prostaglandin F2 receptor negative regulator OS=Mus musculus OX=10090 GN=Ptgfrn PE=1 SV=2 | | | Ptgfrn | | 1.2011 | | 0.0428 | |
| Q9D1C2 | Protein chibby homolog 1 OS=Mus musculus OX=10090 GN=Cby1 PE=1 SV=1 | | | Cby1 | | 1.2143 | | 0.0429 | |
| Q8BTY8 | Sec1 family domain-containing protein 2 OS=Mus musculus OX=10090 GN=Scfd2 PE=1 SV=1 | | | Scfd2 | | 1.2182 | | 0.0449 | |
| P63213 | Guanine nucleotide-binding protein G(I)/G(S)/G(O) subunit gamma-2 OS=Mus musculus OX=10090 GN=Gng2 PE=1 SV=2 | | | Gng2 | | 0.8296 | | 0.0468 | |
| Q8BK30 | NADH dehydrogenase [ubiquinone] flavoprotein 3, mitochondrial OS=Mus musculus OX=10090 GN=Ndufv3 PE=1 SV=1 | | | Ndufv3 | | 0.8163 | | 0.0468 | |
| P60041 | Somatostatin OS=Mus musculus OX=10090 GN=Sst PE=1 SV=1 | | | Sst | | 0.5442 | | 0.0484 | |

| **Table S2 The changes of hippocampal proteins related to synapse structure, activity and organization among young, FMT and old group** | | | | | | | |
| --- | --- | --- | --- | --- | --- | --- | --- |
| **gene** | **Normalized intensity**  **Young** | **Normalized intensity**  **FMT** | | | **Normalized intensity-Old** | | **Description** |
| CamkIIa | 1.1092 | 0.9651 | | | 0.9258 | | post-synapse |
| Bsn | 1.0111 | 0.9995 | | | 0.9893 | | pre-synapse |
| Syn1 | 1.0279 | 1.0169 | | | 0.9552 | | pre-synapse |
| Syn2 | 1.0390 | 1.0320 | | | 0.9289 | | pre-synapse |
| Syn3 | 1.0069 | 0.9991 | | | 0.9940 | | pre-synapse |
| Syp | 1.0342 | 1.0108 | | | 0.9550 | | pre-synapse |
| Vamp3 | 1.0516 | 0.9988 | | | 0.9495 | | pre-synapse |
| Vamp4 | 1.0086 | 1.0011 | | | 0.9903 | | pre-synapse |
| Vamp8 | 1.0566 | 0.9983 | | | 0.9451 | | pre-synapse |
| Homer1 | 1.0598 | 0.9959 | | | 0.9442 | | regulation of synapse structure or activity;post-synapse |
| Abhd17c | 1.0533 | 1.0554 | | | 0.8914 | | regulation of synapse structure or activity |
| Actr2 | 1.1060 | 0.9914 | | | 0.9026 | | regulation of synapse structure or activity |
| Adgrl1 | 1.0227 | 1.0290 | | | 0.9483 | | regulation of synapse structure or activity |
| Cask | 1.0482 | 1.0104 | | | 0.9415 | | regulation of synapse structure or activity |
| Hspa8 | 1.0240 | 1.0418 | | | 0.9342 | | regulation of synapse structure or activity |
| Mdga1 | 1.0665 | 1.0304 | | | 0.9031 | | regulation of synapse structure or activity |
| Nectin1 | 1.0522 | 1.0108 | | | 0.9369 | | regulation of synapse structure or activity |
| Negr1 | 1.1184 | 1.0574 | | | 0.8242 | | regulation of synapse structure or activity |
| Nrcam | 1.0326 | 1.0121 | | | 0.9554 | | regulation of synapse structure or activity |
| Ogt | 1.0427 | 1.0002 | | | 0.9571 | | regulation of synapse structure or activity |
| Ptk2 | 1.0531 | 1.0019 | | | 0.9450 | | regulation of synapse structure or activity |
| Vcp | 1.0234 | 1.0103 | | | 0.9663 | | regulation of synapse structure or activity |
| Cfl1 | 1.0504 | 0.9855 | | | 0.9641 | | regulation of synapse structure or activity;synapse organization |
| Actr3 | 1.1051 | 1.0012 | | | 0.8937 | | regulation of synapse structure or activity;synapse organization |
| Itgb1 | 1.0640 | 1.0204 | | | 0.9156 | | regulation of synapse structure or activity;synapse organization |
| Lrfn4 | 1.1496 | 0.9819 | | | 0.8686 | | regulation of synapse structure or activity;synapse organization |
| Abi2 | 1.0451 | 0.9990 | | | 0.9558 | | regulation of synapse structure or activity;synapse organization |
| Myh10 | 1.0289 | 0.9959 | | | 0.9751 | | regulation of synapse structure or activity;synapse organization |
| Ntrk3 | 1.0660 | 1.0404 | | | 0.8935 | | regulation of synapse structure or activity;synapse organization |
| Pick1 | 1.0596 | 0.9879 | | | 0.9525 | | regulation of synapse structure or activity;synapse organization |
| Ptprs | 1.0231 | 1.0055 | | | 0.9714 | | regulation of synapse structure or activity;synapse organization |
| Cabp1 | 1.0259 | 1.0385 | | | 0.9356 | | synapse organization |
| Fgf13 | 1.0528 | 1.0190 | | | 0.9282 | | synapse organization |
| Insr | 1.0628 | 0.9569 | | | 0.9803 | | synapse organization |
| Nbea | 1.0440 | 1.0050 | | | 0.9510 | | synapse organization |
| Nptn | 1.0379 | 1.0046 | | | 0.9576 | | synapse organization |
| Nrxn3 | 1.0552 | 1.0038 | | | 0.9410 | | synapse organization |
| Plxna4 | 1.0282 | 1.0118 | | | 0.9600 | | synapse organization |
| Sez6l | 1.0253 | 1.0229 | | | 0.9518 | | synapse organization |
| Tnc | 1.1198 | 1.1924 | | | 0.6878 | | synapse organization |
| **gene** | **Normalized intensity**  **Young** | | **Normalized intensity**  **FMT** | **Normalized intensity-Old** | | **Description** | |
| Cacng2 | 0.9419 | | 0.9912 | 1.0669 | | Endocytosis;Lysosome | |
| Cd2ap | 0.8698 | | 0.9501 | 1.1801 | | Endocytosis | |
| Itgam | 0.9118 | | 0.9969 | 1.0912 | | Endocytosis | |
| Sort1 | 0.9379 | | 0.9852 | 1.0769 | | Endocytosis;Lysosome | |

| **Table S3 The correlation coefficient of synapse and microglia marker proteins with the process of endocytosis or lysosome with statistical significance** | | | | | | | | | | |  |
| --- | --- | --- | --- | --- | --- | --- | --- | --- | --- | --- | --- |
| **Endocytosis and lysosome marker** | | **Synapse marker** | | **Speaeman's ρ rho** | | **pvalue** | | **relation** | | **Description** |  |
| Abcb9 | | vamp4 | | -0.9993 | | 0.0238 | | negtive | | Lysosome |  |
| Actr2 | | homer1 | | 0.9999 | | 0.0079 | | positive | | Endocytosis |  |
| Actr2 | | bsn | | 0.9993 | | 0.0235 | | positive | | Endocytosis |  |
| Actr2 | | vamp8 | | 0.9989 | | 0.0294 | | positive | | Endocytosis |  |
| Actr2 | | syn3 | | 0.9989 | | 0.0301 | | positive | | Endocytosis |  |
| Actr2 | | vamp3 | | 0.9986 | | 0.0342 | | positive | | Endocytosis |  |
| Actr3 | | vamp3 | | 0.9996 | | 0.0186 | | positive | | Endocytosis |  |
| Actr3 | | vamp8 | | 0.9993 | | 0.0234 | | positive | | Endocytosis |  |
| Actr3 | | bsn | | 0.9989 | | 0.0293 | | positive | | Endocytosis |  |
| Actr3 | | homer1 | | 0.9975 | | 0.0449 | | positive | | Endocytosis |  |
| Ahsg | | vamp4 | | 0.9985 | | 0.0345 | | positive | | Endocytosis |  |
| Ahsg | | vamp3 | | 0.9979 | | 0.0415 | | positive | | Endocytosis |  |
| Ahsg | | vamp8 | | 0.9974 | | 0.0462 | | positive | | Endocytosis |  |
| Amph | | syn1 | | 0.9992 | | 0.0262 | | positive | | Endocytosis |  |
| Amph | | syn2 | | 0.9991 | | 0.0273 | | positive | | Endocytosis |  |
| Ankrd13a | | syn1 | | -0.9972 | | 0.0473 | | negtive | | Endocytosis |  |
| Ankrd13a | | syp | | -0.9970 | | 0.0492 | | negtive | | Endocytosis |  |
| Ap1b1 | | syn2 | | 0.9998 | | 0.0133 | | positive | | Lysosome |  |
| Ap1b1 | | syn1 | | 0.9980 | | 0.0402 | | positive | | Lysosome |  |
| Ap1s2 | | vamp4 | | 1.0000 | | 0.0052 | | positive | | Lysosome |  |
| Ap2b1 | | vamp4 | | 0.9992 | | 0.0256 | | positive | | Endocytosis |  |
| Ap3m1 | | vamp4 | | 0.9997 | | 0.0151 | | positive | | Endocytosis |  |
| Ap4b1 | | syn3 | | 0.9998 | | 0.0116 | | positive | | Lysosome |  |
| Ap4b1 | | homer1 | | 0.9970 | | 0.0496 | | positive | | Lysosome |  |
| Appl2 | | vamp3 | | -0.9985 | | 0.0351 | | negtive | | Endocytosis |  |
| Appl2 | | vamp8 | | -0.9980 | | 0.0399 | | negtive | | Endocytosis |  |
| Appl2 | | vamp4 | | -0.9979 | | 0.0408 | | negtive | | Endocytosis |  |
| Appl2 | | bsn | | -0.9974 | | 0.0458 | | negtive | | Endocytosis |  |
| Arf5 | | camkIIa | | 0.9980 | | 0.0398 | | positive | | Endocytosis |  |
| Arfgap2 | | syp | | 0.9991 | | 0.0270 | | positive | | Endocytosis |  |
| Arhgap27 | | vamp3 | | -0.9999 | | 0.0086 | | negtive | | Endocytosis |  |
| Arhgap27 | | vamp8 | | -0.9998 | | 0.0134 | | negtive | | Endocytosis |  |
| Arhgap27 | | bsn | | -0.9995 | | 0.0193 | | negtive | | Endocytosis |  |
| Arhgap27 | | homer1 | | -0.9985 | | 0.0349 | | negtive | | Endocytosis |  |
| Arrb2 | | syn3 | | 0.9992 | | 0.0250 | | positive | | Endocytosis |  |
| Arsg | | syn2 | | -0.9997 | | 0.0164 | | negtive | | Lysosome |  |
| Arsg | | syn1 | | -0.9983 | | 0.0372 | | negtive | | Lysosome |  |
| Atg14 | | syn3 | | 1.0000 | | 0.0036 | | positive | | Lysosome |  |
| Atg14 | | homer1 | | 0.9985 | | 0.0345 | | positive | | Lysosome |  |
| Atg14 | | bsn | | 0.9969 | | 0.0500 | | positive | | Lysosome |  |
| Cacng2 | | vamp4 | | -0.9998 | | 0.0134 | | negtive | | Endocytosis;Lysosome |  |
| Calr | | syn2 | | 0.9979 | | 0.0415 | | positive | | Endocytosis |  |
| Cbl | | syp | | 0.9998 | | 0.0112 | | positive | | Endocytosis |  |
| Cd2ap | | syp | | -0.9992 | | 0.0259 | | negtive | | Endocytosis |  |
| Clip3 | | syn1 | | -0.9989 | | 0.0301 | | negtive | | Endocytosis |  |
| Clta | | syn1 | | 0.9997 | | 0.0149 | | positive | | Endocytosis |  |
| Clta | | syn2 | | 0.9982 | | 0.0386 | | positive | | Endocytosis |  |
| Clvs2 | | vamp4 | | 0.9991 | | 0.0269 | | positive | | Lysosome |  |
| Clvs2 | | vamp3 | | 0.9970 | | 0.0491 | | positive | | Lysosome |  |
| Ctsb | | syn1 | | -0.9991 | | 0.0271 | | negtive | | Lysosome |  |
| Cyth1 | | syn2 | | 0.9981 | | 0.0390 | | positive | | Endocytosis |  |
| Gm2a | | syp | | -0.9989 | | 0.0294 | | negtive | | Lysosome |  |
| Grin1 | | vamp3 | | 1.0000 | | 0.0050 | | positive | | Endocytosis |  |
| Grin1 | | vamp8 | | 0.9999 | | 0.0098 | | positive | | Endocytosis |  |
| Grin1 | | bsn | | 0.9997 | | 0.0157 | | positive | | Endocytosis |  |
| Grin1 | | homer1 | | 0.9988 | | 0.0313 | | positive | | Endocytosis |  |
| Grk6 | | camkIIa | | 0.9999 | | 0.0101 | | positive | | Endocytosis |  |
| Gsg1l | | syn1 | | -0.9998 | | 0.0133 | | negtive | | Endocytosis |  |
| Itgam | | vamp3 | | -0.9988 | | 0.0314 | | negtive | | Endocytosis |  |
| Itgam | | vamp8 | | -0.9984 | | 0.0361 | | negtive | | Endocytosis |  |
| Itgam | | bsn | | -0.9978 | | 0.0421 | | negtive | | Endocytosis |  |
| Itgam | | vamp4 | | -0.9975 | | 0.0446 | | negtive | | Endocytosis |  |
| Itgav | | syp | | -0.9994 | | 0.0222 | | negtive | | Endocytosis |  |
| Itgb1 | | syp | | 1.0000 | | 0.0017 | | positive | | Endocytosis |  |
| Kif5c | | vamp4 | | 1.0000 | | 0.0037 | | positive | | Endocytosis |  |
| Lipa | | syn1 | | -0.9975 | | 0.0448 | | negtive | | Endocytosis;Lysosome |  |
| Mesd | | syn2 | | 0.9976 | | 0.0445 | | positive | | Endocytosis |  |
| Pick1 | | syn3 | | 0.9974 | | 0.0462 | | positive | | Endocytosis |  |
| Pikfyve | | camkIIa | | 0.9999 | | 0.0082 | | positive | | Endocytosis |  |
| Pip5k1c | | syn1 | | 0.9978 | | 0.0424 | | positive | | Endocytosis |  |
| Plekhf1 | | syp | | -0.9997 | | 0.0159 | | negtive | | Lysosome |  |
| Ppp3cb | | camkIIa | | 1.0000 | | 0.0004 | | positive | | Endocytosis |  |
| Ppt1 | | syn1 | | -0.9999 | | 0.0065 | | negtive | | Endocytosis |  |
| Ppt1 | | syn2 | | -0.9973 | | 0.0470 | | negtive | | Endocytosis |  |
| Ptk2 | | vamp3 | | 0.9987 | | 0.0322 | | positive | | Endocytosis |  |
| Ptk2 | | vamp8 | | 0.9983 | | 0.0369 | | positive | | Endocytosis |  |
| Ptk2 | | bsn | | 0.9977 | | 0.0429 | | positive | | Endocytosis |  |
| Ptk2 | | vamp4 | | 0.9976 | | 0.0438 | | positive | | Endocytosis |  |
| Rab27a | | syn3 | | 0.9999 | | 0.0070 | | positive | | Endocytosis |  |
| Rab27a | | homer1 | | 0.9988 | | 0.0310 | | positive | | Endocytosis |  |
| Rab27a | | bsn | | 0.9973 | | 0.0466 | | positive | | Endocytosis |  |
| Rab7 | | syp | | 0.9992 | | 0.0247 | | positive | | Endocytosis |  |
| Rab7a | | syp | | 0.9992 | | 0.0247 | | positive | | Lysosome |  |
| Rbsn | | syn2 | | 0.9995 | | 0.0210 | | positive | | Endocytosis |  |
| Rbsn | | syn1 | | 0.9987 | | 0.0326 | | positive | | Endocytosis |  |
| Sh3gl1 | | syn2 | | 0.9999 | | 0.0085 | | positive | | Endocytosis |  |
| Sh3gl3 | | syn3 | | 0.9991 | | 0.0273 | | positive | | Endocytosis |  |
| Sh3glb2 | | syn1 | | 0.9995 | | 0.0197 | | positive | | Endocytosis |  |
| Sh3kbp1 | | vamp4 | | -0.9997 | | 0.0149 | | negtive | | Endocytosis |  |
| Snap25 | | vamp4 | | 0.9991 | | 0.0276 | | positive | | Endocytosis |  |
| Snx2 | | homer1 | | 1.0000 | | 0.0033 | | positive | | Endocytosis |  |
| Snx2 | | bsn | | 0.9998 | | 0.0122 | | positive | | Endocytosis |  |
| Snx2 | | vamp8 | | 0.9996 | | 0.0182 | | positive | | Endocytosis |  |
| Snx2 | | vamp3 | | 0.9994 | | 0.0229 | | positive | | Endocytosis |  |
| Snx2 | | syn3 | | 0.9979 | | 0.0414 | | positive | | Endocytosis |  |
| Sort1 | | syp | | -0.9988 | | 0.0308 | | negtive | | Endocytosis;Lysosome |  |
| Stam2 | | syp | | -1.0000 | | 0.0063 | | negtive | | Endocytosis |  |
| Ston2 | | vamp4 | | 0.9984 | | 0.0365 | | positive | | Endocytosis |  |
| Ston2 | | syp | | 0.9973 | | 0.0471 | | positive | | Endocytosis |  |
| Vcp | | syp | | 0.9974 | | 0.0457 | | positive | | Lysosome |  |
| Vps28 | | syn3 | | 0.9997 | | 0.0143 | | positive | | Endocytosis |  |
| Vps28 | | homer1 | | 0.9993 | | 0.0237 | | positive | | Endocytosis |  |
| Vps28 | | bsn | | 0.9981 | | 0.0393 | | positive | | Endocytosis |  |
| Vps28 | | vamp8 | | 0.9975 | | 0.0452 | | positive | | Endocytosis |  |
| Vps28 | | vamp3 | | 0.9969 | | 0.0500 | | positive | | Endocytosis |  |
| Vps39 | | syn2 | | -0.9995 | | 0.0205 | | negtive | | Lysosome |  |
| Vta1 | | vamp3 | | 1.0000 | | 0.0042 | | positive | | Endocytosis |  |
| Vta1 | | vamp8 | | 0.9999 | | 0.0089 | | positive | | Endocytosis |  |
| Vta1 | | bsn | | 0.9997 | | 0.0149 | | positive | | Endocytosis |  |
| Vta1 | | homer1 | | 0.9989 | | 0.0304 | | positive | | Endocytosis |  |
| Washc2 | | syp | | -0.9999 | | 0.0065 | | negtive | | Endocytosis |  |
| Washc5 | | syp | | -0.9999 | | 0.0072 | | negtive | | Endocytosis |  |
| Wdr54 | | syp | | -0.9990 | | 0.0280 | | negtive | | Endocytosis |  |
| **Endocytosis and lysosome marker** | **Glial marker** | | **Speaeman's ρ rho** | | **pvalue** | | **relation** | | **Description** | | |
| Amph | ALDH1L1 | | -0.9999 | | 0.0083 | | negtive | | Endocytosis | | |
| Ap1b1 | ALDH1L1 | | -0.9994 | | 0.0223 | | negtive | | Lysosome | | |
| Arsg | ALDH1L1 | | 0.9995 | | 0.0192 | | positive | | Lysosome | | |
| Clip3 | ALDH1L1 | | 0.9972 | | 0.0481 | | positive | | Endocytosis | | |
| Clta | ALDH1L1 | | -1.0000 | | 0.0030 | | negtive | | Endocytosis;Lysosome | | |
| Ctsb | ALDH1L1 | | 0.9975 | | 0.0451 | | positive | | Lysosome | | |
| Gsg1l | ALDH1L1 | | 0.9988 | | 0.0312 | | positive | | Endocytosis | | |
| Ppt1 | ALDH1L1 | | 0.9998 | | 0.0114 | | positive | | Endocytosis;Lysosome | | |
| Rbsn | ALDH1L1 | | -0.9997 | | 0.0146 | | negtive | | Endocytosis | | |
| Sh3gl1 | ALDH1L1 | | -0.9976 | | 0.0441 | | negtive | | Endocytosis | | |
| Sh3glb2 | ALDH1L1 | | -0.9982 | | 0.0377 | | negtive | | Endocytosis | | |
| Arrb1 | c4b | | 0.9999 | | 0.0102 | | positive | | Endocytosis | | |
| Calr | c4b | | -1.0000 | | 0.0064 | | negtive | | Endocytosis | | |
| Cyth1 | c4b | | -0.9999 | | 0.0089 | | negtive | | Endocytosis | | |
| Fcho2 | c4b | | -0.9982 | | 0.0377 | | negtive | | Endocytosis | | |
| Gla | c4b | | 0.9969 | | 0.0498 | | positive | | Lysosome | | |
| Lamtor1 | c4b | | 0.9996 | | 0.0174 | | positive | | Lysosome | | |
| Lrpap1 | c4b | | 1.0000 | | 0.0054 | | positive | | Endocytosis | | |
| Mesd | c4b | | -1.0000 | | 0.0034 | | negtive | | Endocytosis | | |
| Scarb2 | c4b | | 0.9990 | | 0.0288 | | positive | | Endocytosis;Lysosome | | |
| Sh3gl1 | c4b | | -0.9981 | | 0.0394 | | negtive | | Endocytosis | | |
| Vps39 | c4b | | 0.9991 | | 0.0274 | | positive | | Lysosome | | |
| Ap1b1 | cd44 | | -0.9983 | | 0.0372 | | negtive | | Lysosome | | |
| Arrb1 | cd44 | | 0.9986 | | 0.0342 | | positive | | Endocytosis | | |
| Arsg | cd44 | | 0.9980 | | 0.0403 | | positive | | Lysosome | | |
| Calr | cd44 | | -0.9996 | | 0.0176 | | negtive | | Endocytosis | | |
| Cyth1 | cd44 | | -0.9997 | | 0.0151 | | negtive | | Endocytosis | | |
| Lamtor1 | cd44 | | 0.9979 | | 0.0414 | | positive | | Lysosome | | |
| Lrpap1 | cd44 | | 0.9989 | | 0.0294 | | positive | | Endocytosis | | |
| Mesd | cd44 | | -0.9995 | | 0.0206 | | negtive | | Endocytosis | | |
| Rbsn | cd44 | | -0.9975 | | 0.0449 | | negtive | | Endocytosis | | |
| Sh3gl1 | cd44 | | -0.9997 | | 0.0154 | | negtive | | Endocytosis | | |
| Vps39 | cd44 | | 1.0000 | | 0.0034 | | positive | | Lysosome | | |
| Cacng2 | ddr1 | | 0.9979 | | 0.0412 | | positive | | Endocytosis;Lysosome | | |
| Cbl | ddr1 | | -0.9980 | | 0.0402 | | negtive | | Endocytosis | | |
| Itgav | ddr1 | | 0.9999 | | 0.0068 | | positive | | Endocytosis | | |
| Itgb1 | ddr1 | | -0.9988 | | 0.0307 | | negtive | | Endocytosis | | |
| Plekhf1 | ddr1 | | 0.9998 | | 0.0131 | | positive | | Lysosome | | |
| Rab7 | ddr1 | | -1.0000 | | 0.0043 | | negtive | | Lysosome | | |
| Rab7a | ddr1 | | -1.0000 | | 0.0043 | | negtive | | Endocytosis | | |
| Sh3kbp1 | ddr1 | | 0.9981 | | 0.0397 | | positive | | Endocytosis | | |
| Snap25 | ddr1 | | -0.9991 | | 0.0270 | | negtive | | Endocytosis | | |
| Sort1 | ddr1 | | 1.0000 | | 0.0018 | | positive | | Endocytosis;Lysosome | | |
| Stam2 | ddr1 | | 0.9994 | | 0.0227 | | positive | | Endocytosis | | |
| Ston2 | ddr1 | | -0.9996 | | 0.0181 | | negtive | | Endocytosis | | |
| Washc2 | ddr1 | | 0.9994 | | 0.0225 | | positive | | Endocytosis | | |
| Washc5 | ddr1 | | 0.9984 | | 0.0362 | | positive | | Endocytosis;Lysosome | | |
| Ap1s2 | dnajb14 | | -0.9981 | | 0.0389 | | negtive | | Lysosome | | |
| Ap3m1 | dnajb14 | | -0.9971 | | 0.0487 | | negtive | | Endocytosis;Lysosome | | |
| Cacng2 | dnajb14 | | 0.9995 | | 0.0203 | | positive | | Endocytosis;Lysosome | | |
| Itgav | dnajb14 | | 0.9990 | | 0.0278 | | positive | | Endocytosis | | |
| Kif5c | dnajb14 | | -0.9983 | | 0.0374 | | negtive | | Endocytosis | | |
| Plekhf1 | dnajb14 | | 0.9986 | | 0.0340 | | positive | | Lysosome | | |
| Rab7 | dnajb14 | | -0.9992 | | 0.0252 | | negtive | | Lysosome | | |
| Rab7a | dnajb14 | | -0.9992 | | 0.0252 | | negtive | | Endocytosis | | |
| Sh3kbp1 | dnajb14 | | 0.9996 | | 0.0188 | | positive | | Endocytosis | | |
| Snap25 | dnajb14 | | -1.0000 | | 0.0061 | | negtive | | Endocytosis | | |
| Sort1 | dnajb14 | | 0.9995 | | 0.0192 | | positive | | Endocytosis;Lysosome | | |
| Stam2 | dnajb14 | | 0.9976 | | 0.0437 | | positive | | Endocytosis | | |
| Ston2 | dnajb14 | | -1.0000 | | 0.0028 | | negtive | | Endocytosis | | |
| Washc2 | dnajb14 | | 0.9977 | | 0.0434 | | positive | | Endocytosis | | |
| Actr2 | iba-1 | | -0.9990 | | 0.0283 | | negtive | | Endocytosis | | |
| Ap4b1 | iba-1 | | -0.9998 | | 0.0134 | | negtive | | Lysosome | | |
| Arrb2 | iba-1 | | -0.9991 | | 0.0269 | | negtive | | Endocytosis | | |
| Atg14 | iba-1 | | -1.0000 | | 0.0017 | | negtive | | Lysosome | | |
| Pick1 | iba-1 | | -0.9971 | | 0.0481 | | negtive | | Endocytosis | | |
| Rab27a | iba-1 | | -1.0000 | | 0.0051 | | negtive | | Endocytosis | | |
| Sh3gl3 | iba-1 | | -0.9990 | | 0.0291 | | negtive | | Endocytosis | | |
| Snx2 | iba-1 | | -0.9981 | | 0.0395 | | negtive | | Endocytosis | | |
| Vps28 | iba-1 | | -0.9998 | | 0.0125 | | negtive | | Endocytosis | | |
| Abcb9 | itgam | | 0.9995 | | 0.0208 | | positive | | Lysosome | | |
| Actr3 | itgam | | -0.9998 | | 0.0127 | | negtive | | Endocytosis | | |
| Ahsg | itgam | | -0.9999 | | 0.0101 | | negtive | | Endocytosis | | |
| Ap1s2 | itgam | | -0.9981 | | 0.0394 | | negtive | | Lysosome | | |
| Ap2b1 | itgam | | -0.9996 | | 0.0190 | | negtive | | Lysosome | | |
| Ap3m1 | itgam | | -0.9989 | | 0.0295 | | negtive | | Endocytosis;Lysosome | | |
| Appl2 | itgam | | 1.0000 | | 0.0038 | | positive | | Endocytosis | | |
| Arhgap27 | itgam | | 0.9994 | | 0.0228 | | positive | | Endocytosis | | |
| Clvs2 | itgam | | -0.9996 | | 0.0177 | | negtive | | Lysosome | | |
| Grin1 | itgam | | -0.9991 | | 0.0264 | | negtive | | Endocytosis | | |
| Kif5c | itgam | | -0.9979 | | 0.0409 | | negtive | | Endocytosis | | |
| Ptk2 | itgam | | -1.0000 | | 0.0008 | | negtive | | Endocytosis | | |
| Vta1 | itgam | | -0.9991 | | 0.0272 | | negtive | | Endocytosis | | |
| Amph | lgi2 | | -0.9998 | | 0.0129 | | negtive | | Endocytosis | | |
| Ap1b1 | lgi2 | | -0.9991 | | 0.0269 | | negtive | | Lysosome | | |
| Arsg | lgi2 | | 0.9993 | | 0.0238 | | positive | | Lysosome | | |
| Clip3 | lgi2 | | 0.9977 | | 0.0434 | | positive | | Endocytosis | | |
| Clta | lgi2 | | -1.0000 | | 0.0016 | | negtive | | Endocytosis;Lysosome | | |
| Ctsb | lgi2 | | 0.9980 | | 0.0405 | | positive | | Lysosome | | |
| Gsg1l | lgi2 | | 0.9991 | | 0.0266 | | positive | | Endocytosis | | |
| Ppt1 | lgi2 | | 0.9999 | | 0.0068 | | positive | | Endocytosis;Lysosome | | |
| Rbsn | lgi2 | | -0.9995 | | 0.0192 | | negtive | | Endocytosis | | |
| Sh3gl1 | lgi2 | | -0.9971 | | 0.0487 | | negtive | | Endocytosis | | |
| Sh3glb2 | lgi2 | | -0.9987 | | 0.0331 | | negtive | | Endocytosis | | |
| Amph | mlc1 | | -0.9982 | | 0.0384 | | negtive | | Endocytosis | | |
| Ap1b1 | mlc1 | | -0.9993 | | 0.0244 | | negtive | | Lysosome | | |
| Arrb1 | mlc1 | | 0.9973 | | 0.0471 | | positive | | Endocytosis | | |
| Arsg | mlc1 | | 0.9991 | | 0.0274 | | positive | | Lysosome | | |
| Calr | mlc1 | | -0.9989 | | 0.0305 | | negtive | | Endocytosis | | |
| Clta | mlc1 | | -0.9970 | | 0.0497 | | negtive | | Endocytosis;Lysosome | | |
| Cyth1 | mlc1 | | -0.9990 | | 0.0280 | | negtive | | Endocytosis | | |
| Lrpap1 | mlc1 | | 0.9978 | | 0.0422 | | positive | | Endocytosis | | |
| Mesd | mlc1 | | -0.9986 | | 0.0335 | | negtive | | Endocytosis | | |
| Rbsn | mlc1 | | -0.9987 | | 0.0320 | | negtive | | Endocytosis | | |
| Sh3gl1 | mlc1 | | -1.0000 | | 0.0025 | | negtive | | Endocytosis | | |
| Vps39 | mlc1 | | 0.9999 | | 0.0095 | | positive | | Lysosome | | |
| Ap1b1 | olfml3 | | -0.9973 | | 0.0465 | | negtive | | Lysosome | | |
| Arrb1 | olfml3 | | 0.9992 | | 0.0250 | | positive | | Endocytosis | | |
| Arsg | olfml3 | | 0.9970 | | 0.0495 | | positive | | Lysosome | | |
| Calr | olfml3 | | -0.9999 | | 0.0084 | | negtive | | Endocytosis | | |
| Cyth1 | olfml3 | | -1.0000 | | 0.0059 | | negtive | | Endocytosis | | |
| Lamtor1 | olfml3 | | 0.9987 | | 0.0322 | | positive | | Lysosome | | |
| Lrpap1 | olfml3 | | 0.9995 | | 0.0202 | | positive | | Endocytosis | | |
| Mesd | olfml3 | | -0.9998 | | 0.0114 | | negtive | | Endocytosis | | |
| Scarb2 | olfml3 | | 0.9977 | | 0.0435 | | positive | | Endocytosis;Lysosome | | |
| Sh3gl1 | olfml3 | | -0.9993 | | 0.0246 | | negtive | | Endocytosis | | |
| Vps39 | olfml3 | | 0.9998 | | 0.0126 | | positive | | Lysosome | | |
| Igf2r | slc4a4 | | -1.0000 | | 0.0027 | | negtive | | Endocytosis;Lysosome | | |
| Mib1 | slc4a4 | | -0.9990 | | 0.0289 | | negtive | | Endocytosis | | |
| Myd88 | slc4a4 | | 0.9999 | | 0.0104 | | positive | | Endocytosis | | |
| Pacsin1 | slc4a4 | | 0.9984 | | 0.0364 | | positive | | Endocytosis | | |
| Vps4b | slc4a4 | | -0.9998 | | 0.0134 | | negtive | | Endocytosis | | |
| Washc4 | slc4a4 | | 0.9970 | | 0.0493 | | positive | | Endocytosis | | |

| **Table S4 Differential bacterial species between young, FMT and old groups resulted from metagenome sequencing analysis** | | | | | | | | | | | | |
| --- | --- | --- | --- | --- | --- | --- | --- | --- | --- | --- | --- | --- |
| **Species name** | | | | | | | **Group** | **Mean** | | **LDA_value** | | **Pvalue** |
| *Ileibacterium_valens* | | | | | | | old | 4.9579 | | 4.6466 | | 0.0090 |
| *Lachnospiraceae_bacterium* | | | | | | | young | 5.3056 | | 4.4467 | | 0.0472 |
| *Erysipelotrichaceae_bacterium* | | | | | | | old | 4.6913 | | 4.3636 | | 0.0163 |
| *Allobaculum*_sp__539 | | | | | | | old | 4.4065 | | 4.1177 | | 0.0090 |
| *Bifidobacterium_pseudolongum* | | | | | | | young | 4.5851 | | 4.0472 | | 0.0090 |
| *Faecalibaculum_rodentium* | | | | | | | old | 4.3196 | | 3.9439 | | 0.0090 |
| *Acetatifactor_muris* | | | | | | | young | 4.1776 | | 3.7935 | | 0.0163 |
| *Eggerthellaceae_bacterium* | | | | | | | young | 4.2544 | | 3.6968 | | 0.0090 |
| *unclassified_g__Allobaculum* | | | | | | | old | 3.9660 | | 3.5801 | | 0.0090 |
| *Prevotella*_sp__MGM2 | | | | | | | young | 3.9214 | | 3.5777 | | 0.0090 |
| *Erysipelotrichaceae_bacterium*_NYU_BL_F16 | | | | | | | old | 3.8530 | | 3.5437 | | 0.0090 |
| *Lachnospiraceae_bacterium*_A2 | | | | | | | young | 3.7610 | | 3.4648 | | 0.0090 |
| *Erysipelotrichaceae_bacterium*_NYU_BL_E8 | | | | | | | old | 3.5733 | | 3.2603 | | 0.0090 |
| *Alistipes*_sp_ | | | | | | | young | 3.6676 | | 3.2552 | | 0.0090 |
| *Dubosiella_newyorkensis* | | | | | | | old | 3.5645 | | 3.2182 | | 0.0090 |
| bacterium_D16_34 | | | | | | | young | 3.6515 | | 3.2140 | | 0.0090 |
| *Alistipes*_sp__DSM_112343 | | | | | | | young | 3.4340 | | 3.0900 | | 0.0090 |
| *Adlercreutzia*_sp__DSM_109821 | | | | | | | young | 3.5179 | | 3.0803 | | 0.0090 |
| *Allobaculum*_sp__Allo2 | | | | | | | old | 3.5630 | | 3.0701 | | 0.0472 |
| *Duncaniella_dubosii* | | | | | | | young | 3.4363 | | 2.9783 | | 0.0472 |
| *Bacteroidaceae_bacterium* | | | | | | | young | 3.3429 | | 2.9238 | | 0.0090 |
| *Acutalibacte*r_sp_ | | | | | | | young | 3.2986 | | 2.8967 | | 0.0090 |
| *Muribaculum*_sp__NM65_B17 | | | | | | | old | 3.3823 | | 2.8850 | | 0.0163 |
| *Muribaculaceae_bacterium*_Isolate_080__Janvier_ | | | | | | | young | 3.2790 | | 2.8076 | | 0.0283 |
| *Staphylococcus_xylosus* | | | | | | | old | 3.1101 | | 2.7884 | | 0.0472 |
| *Erysipelotrichaceae_bacterium*_OPF54 | | | | | | | old | 3.1530 | | 2.7660 | | 0.0163 |
| *Veillonella*_sp_ | | | | | | | old | 3.0712 | | 2.7599 | | 0.0090 |
| *Enterorhabdus*_sp__P55 | | | | | | | young | 3.2605 | | 2.7288 | | 0.0283 |
| *Butyrivibrio*_sp_ | | | | | | | young | 3.2246 | | 2.6863 | | 0.0283 |
| *Muribaculaceae_bacterium*_Isolate_102__HZI_ | | | | | | | old | 3.1101 | | 2.6837 | | 0.0090 |
| *Granulimonas_faecalis* | | | | | | | old | 2.9585 | | 2.6624 | | 0.0090 |
| *Firmicutes_bacterium*_M10_2 | | | | | | | old | 3.0010 | | 2.6262 | | 0.0163 |
| unclassified_g__Caniella | | | | | | | old | 2.8603 | | 2.5636 | | 0.0090 |
| *Adlercreutzia*_sp__DSM_108611 | | | | | | | young | 3.0840 | | 2.5335 | | 0.0090 |
| unclassified_g__Staphylococcus | | | | | | | young | 2.8275 | | 2.5091 | | 0.0283 |
| *Lachnospiraceae_bacterium*_3_1 | | | | | | | young | 3.1827 | | 2.5014 | | 0.0283 |
| *Bifidobacterium_cuniculi* | | | | | | | young | 2.8114 | | 2.5006 | | 0.0090 |
| *Amedibacillus_dolichus* | | | | | | | old | 2.7862 | | 2.3840 | | 0.0090 |
| *Staphylococcus_nepalensis* | | | | | | | young | 2.6680 | | 2.3819 | | 0.0163 |
| *Caniella*_sp__DSM_110983 | | | | | | | old | 2.6864 | | 2.3793 | | 0.0090 |
| *Lactobacillus_acidophilus* | | | | | | | old | 2.7631 | | 2.3790 | | 0.0090 |
| *Ruminococcus_flavefaciens* | | | | | | | young | 3.1378 | | 2.3739 | | 0.0283 |
| *unclassified_g__Neglectibacter* | | | | | | | young | 2.9023 | | 2.3722 | | 0.0090 |
| *Caniella*_sp__DSM_105314 | | | | | | | old | 2.6677 | | 2.3691 | | 0.0090 |
| *Faecalicoccus_pleomorphus* | | | | | | | old | 2.7100 | | 2.3689 | | 0.0090 |
| *Eubacterium*_sp__14_2 | | | | | | | young | 2.9147 | | 2.3667 | | 0.0163 |
| *Limosilactobacillus_reuteri* | | | | | | | old | 2.7570 | | 2.3559 | | 0.0283 |
| *Candidatus_Ventrousia_excrementavium* | | | | | | | old | 2.6595 | | 2.3448 | | 0.0090 |
| *Candidatus_Desulfovibrio_gallistercoris* | | | | | | | young | 3.0099 | | 2.3181 | | 0.0163 |
| *Eisenbergiella_tayi* | | | | | | | young | 2.7508 | | 2.3176 | | 0.0090 |
| *Eggerthella*_sp_ | | | | | | | young | 2.7756 | | 2.3162 | | 0.0090 |
| *Blautia*_sp_ | | | | | | | young | 2.8941 | | 2.2940 | | 0.0090 |
| bacterium_D16_50 | | | | | | | young | 2.9352 | | 2.2919 | | 0.0163 |
| bacterium_D16_76 | | | | | | | young | 2.9204 | | 2.2850 | | 0.0090 |
| *Eisenbergiella_massiliensis* | | | | | | | young | 2.7153 | | 2.2720 | | 0.0090 |
| *Acutalibacter_muris* | | | | | | | young | 2.9133 | | 2.2402 | | 0.0090 |
| *Acetatifactor*_sp_ | | | | | | | young | 2.7306 | | 2.2369 | | 0.0090 |
| *Muribaculum_gordoncarteri* | | | | | | | old | 2.7982 | | 2.2222 | | 0.0163 |
| unclassified_g__*Muribaculum* | | | | | | | old | 2.6526 | | 2.2188 | | 0.0090 |
| *Adlercreutzia_equolifaciens* | | | | | | | young | 2.9347 | | 2.2134 | | 0.0283 |
| *Sporofaciens_musculi* | | | | | | | young | 3.1233 | | 2.1951 | | 0.0472 |
| *Absicoccus_porci* | | | | | | | old | 2.6178 | | 2.1817 | | 0.0090 |
| unclassified_g__*Bifidobacterium* | | | | | | | young | 2.6816 | | 2.1817 | | 0.0163 |
| *Allobaculum*_sp_ | | | | | | | old | 2.7780 | | 2.1723 | | 0.0472 |
| *Clostridiales*_Family_XIII_bacterium | | | | | | | old | 2.4884 | | 2.1605 | | 0.0283 |
| *Coriobacteriia_bacterium* | | | | | | | young | 2.7523 | | 2.1386 | | 0.0090 |
| *Adlercreutzia*_sp__JBNU_10 | | | | | | | young | 2.7263 | | 2.1335 | | 0.0090 |
| *Flintibacter*_sp__DSM_110149 | | | | | | | young | 2.9557 | | 2.1097 | | 0.0472 |
| *Candidatus_Gastranaerophilales_bacterium* | | | | | | | young | 2.5686 | | 2.0886 | | 0.0090 |
| *Candidatus_Spyradocola_merdavium* | | | | | | | old | 2.3532 | | 2.0729 | | 0.0090 |
| unclassified_f__*Erysipelotrichaceae* | | | | | | | old | 2.3841 | | 2.0611 | | 0.0090 |
| *Clostridium*_sp__CAG_594 | | | | | | | old | 2.4032 | | 2.0386 | | 0.0283 |
| *Clostridioides_difficile* | | | | | | | young | 2.9521 | | 2.0279 | | 0.0283 |
| *Lacrimispora_amygdalina* | | | | | | | young | 2.4234 | | 2.0217 | | 0.0090 |
| *Coprobacillus*_sp__8_1_38FAA | | | | | | | old | 2.3868 | | 2.0084 | | 0.0090 |
| **Table.S5 Differential metabolites between young and old groups resulted from serum metabolomic analysis** | | | | | | | | | | | | |
| **Metabolite** | | | **Young average** | | | **Old average** | | | **Pvalue** | | **Foldchange** | |
| Indoleacetic acid | | | 1.5993 | | | 0.2898 | | | 0.0000 | | 0.1812 | |
| Malic acid | | | 26.4548 | | | 12.9930 | | | 0.0000 | | 0.4911 | |
| Fumaric acid | | | 16.3522 | | | 8.4222 | | | 0.0000 | | 0.5150 | |
| 2-Hydroxy-3-methylbutyric acid | | | 13.9779 | | | 7.4226 | | | 0.0000 | | 0.5310 | |
| Oxoglutaric acid | | | 64.0363 | | | 36.3227 | | | 0.0000 | | 0.5672 | |
| Homovanillic acid | | | 0.7172 | | | 0.5736 | | | 0.0000 | | 0.7998 | |
| Methylcysteine | | | 0.7883 | | | 1.3873 | | | 0.0001 | | 1.7598 | |
| 3-Hydroxyisovaleric acid | | | 0.1858 | | | 0.0494 | | | 0.0001 | | 0.2657 | |
| Imidazolepropionic acid | | | 0.5009 | | | 0.3576 | | | 0.0001 | | 0.7139 | |
| Ketoleucine_4-Methyl-2-oxopentanoate | | | 18.1907 | | | 9.4502 | | | 0.0001 | | 0.5195 | |
| Hydroxypropionic acid | | | 28.9606 | | | 19.9960 | | | 0.0001 | | 0.6905 | |
| Oxoadipic acid | | | 2.0126 | | | 0.8915 | | | 0.0002 | | 0.4430 | |
| Hydroxyphenyllactic acid | | | 1.2749 | | | 0.9278 | | | 0.0002 | | 0.7277 | |
| Glycolic acid | | | 57.1931 | | | 32.4232 | | | 0.0004 | | 0.5669 | |
| Picolinic acid | | | 0.6931 | | | 0.5844 | | | 0.0008 | | 0.8432 | |
| Octanoic acid | | | 10.4700 | | | 6.6500 | | | 0.0019 | | 0.6351 | |
| Arachidonic acid | | | 13.9768 | | | 20.0468 | | | 0.0031 | | 1.4343 | |
| Glutaric acid | | | 1.5093 | | | 1.1915 | | | 0.0032 | | 0.7894 | |
| Methylsuccinic acid | | | 0.4730 | | | 0.3580 | | | 0.0035 | | 0.7569 | |
| N-Phenylacetylphenylalanine | | | 0.3513 | | | 0.3017 | | | 0.0040 | | 0.8588 | |
| Phenylacetic acid | | | 11.6533 | | | 5.4345 | | | 0.0043 | | 0.4664 | |
| Propionic acid | | | 10.7071 | | | 5.4316 | | | 0.0045 | | 0.5073 | |
| Adrenic acid | | | 3.6930 | | | 7.6289 | | | 0.0055 | | 2.0658 | |
| TDCA | | | 0.2008 | | | 0.3221 | | | 0.0062 | | 1.6045 | |
| Gluconolactone | | | 48.0545 | | | 25.7097 | | | 0.0069 | | 0.5350 | |
| alpha-Linolenic acid | | | 13.0580 | | | 7.8068 | | | 0.0069 | | 0.5979 | |
| THDCA | | | 0.3135 | | | 0.9559 | | | 0.0070 | | 3.0490 | |
| Citrulline | | | 63.7033 | | | 49.8570 | | | 0.0072 | | 0.7826 | |
| Aspartic acid | | | 19.3430 | | | 33.4781 | | | 0.0079 | | 1.7308 | |
| Cinnamic acid | | | 0.1136 | | | 0.0665 | | | 0.0093 | | 0.5849 | |
| 3-Methyl-2-oxopentanoic acid | | | 32.4800 | | | 23.1869 | | | 0.0094 | | 0.7139 | |
| Azelaic acid | | | 0.3170 | | | 0.2738 | | | 0.0096 | | 0.8638 | |
| Proline | | | 103.1258 | | | 83.1090 | | | 0.0105 | | 0.8059 | |
| Indolelactic acid | | | 1.4849 | | | 1.2104 | | | 0.0112 | | 0.8151 | |
| alpha-Hydroxyisobutyric acid | | | 8.7052 | | | 10.4850 | | | 0.0121 | | 1.2044 | |
| Suberic acid | | | 0.2443 | | | 0.1898 | | | 0.0135 | | 0.7769 | |
| NorDCA | | | 0.2715 | | | 0.1582 | | | 0.0139 | | 0.5826 | |
| Aminocaproic acid | | | 0.7005 | | | 0.5286 | | | 0.0190 | | 0.7547 | |
| Citramalic acid | | | 0.3261 | | | 0.2546 | | | 0.0246 | | 0.7808 | |
| Tryptophan | | | 62.5293 | | | 74.6513 | | | 0.0254 | | 1.1939 | |
| 2-Phenylpropionate | | | 0.5819 | | | 0.3616 | | | 0.0258 | | 0.6213 | |
| Ethylmethylacetic acid_2-methylbutyrate | | | 1.2927 | | | 0.9865 | | | 0.0314 | | 0.7631 | |
| Glycine | | | 249.4576 | | | 199.5674 | | | 0.0320 | | 0.8000 | |
| Hydrocinnamic acid | | | 1.1627 | | | 0.6727 | | | 0.0335 | | 0.5785 | |
| gamma-Linolenic acid | | | 1.5940 | | | 1.1444 | | | 0.0370 | | 0.7179 | |
| TUDCA | | | 0.0283 | | | 0.1058 | | | 0.0415 | | 3.7435 | |
| Hippuric acid | | | 3.4879 | | | 1.3720 | | | 0.0421 | | 0.3934 | |
| Glyceric acid | | | 1.3850 | | | 2.6413 | | | 0.0422 | | 1.9071 | |
| Arginine | | | 156.3469 | | | 129.8073 | | | 0.0457 | | 0.8303 | |
| Methionine | | | 67.5451 | | | 52.6224 | | | 0.0477 | | 0.7791 | |
| **Table S6 Clinical characteristics of the subjects** | | | |  |  |  |  |  |  |  |  |  |
| **Parameters** | **CI n=21** | | **HC n=24** |  |  |  |  |  |  |  |  |  |
| Gender(female/male) | 13/8 | | 13/11 |  |  |  |  |  |  |  |  |  |
| Age (mean ± SD) | 70.8 ± 4.1 | | 23.8 ± 3.1 |  |  |  |  |  |  |  |  |  |
| Aβ42 (mean ± SD) | 7.3 ± 3.0 | | NA |  |  |  |  |  |  |  |  |  |
| Aβ40 (mean ± SD) | 203.8 ± 63.5 | | NA |  |  |  |  |  |  |  |  |  |
| Aβ42/Aβ40 (mean ± SD) | 0.04 ± 0.01 | | NA |  |  |  |  |  |  |  |  |  |
| Tau (mean ± SD) | 1.9 ± 0.5 | | NA |  |  |  |  |  |  |  |  |  |
| p-tau181 (mean ± SD) | 2.8 ± 1.5 | | NA |  |  |  |  |  |  |  |  |  |
| MMSE (mean ± SD) | 21.7 ± 4.5 | | NA |  |  |  |  |  |  |  |  |  |
| MoCA_B (mean ± SD) | 16.2 ± 4.3 | | NA |  |  |  |  |  |  |  |  |  |
| ACEIII (mean ± SD) | 61.4 ± 14.1 | | NA |  |  |  |  |  |  |  |  |  |
| NPI-Q (mean ± SD) | 2.8 ± 2.4 | | NA |  |  |  |  |  |  |  |  |  |
| Aβ SUVR (mean ± SD) | 1.4 ± 0.1 | | NA |  |  |  |  |  |  |  |  |  |
| FDG SUVR (mean ± SD) | 1.1 ± 0.07 | | NA |  |  |  |  |  |  |  |  |  |
| MTA (mean ± SD) | 0.9 ± 0.6 | | NA |  |  |  |  |  |  |  |  |  |
|  |  | |  |  |  |  |  |  |  |  |  |  |
| NA: Not available |  | |  |  |  |  |  |  |  |  |  |  |

**Table S7 The primers used in the current study.**

| **GeneSymbol** | **Forward/Reverse** | **Primer (5' to 3')** |
| --- | --- | --- |
| *Iba1* | Forward | TCTGCCGTCCAAACTTGAAGCC |
|  | Reverse | CTCTTCAGCTCTAGGTGGGTCT |
| *Cd68* | Forward | GGCGGTGGAATACAATGTGTCC |
|  | Reverse | AGCAGGTCAAGGTGAACAGCTG |
| *Nlrp3* | Forward | ATCAACAGGCGAGACCTCTG |
|  | Reverse | GTCCTCCTGGCATACCATAGA |
| *Caspase1* | Forward | GGCACATTTCCAGGACTGACTG |
|  | Reverse | GCAAGACGTGTACGAGTGGTTG |
| *Nfκb* | Forward | ATGGCAGACGATGATCCCTAC |
|  | Reverse | CGGAATCGAAATCCCCTCTGTT |
| *ASC* | Forward | CTTGTCAGGGGATGAACTCAAAA |
|  | Reverse | GCCATACGACTCCAGATAGTAGC |
| *Tnfα* | Forward | TAGCCAGGAGGGAGAACAGA |
|  | Reverse | TTTTCTGGAGGGAGATGTGG |
| *Il6* | Forward | CTCTCCGCAAGAGACTTCCA |
|  | Reverse | CCTCCGACTTGTGAAGTGGT |
| *Il1β* | Forward | CACAGCAGCATCTCGACAAG |
|  | Reverse | CCTGCAGTGCAGCTGTCTAA |
| *Il18* | Forward | GACAGCCTGTGTTCGAGGATATG |
|  | Reverse | TGTTCTTACAGGAGAGGGTAGAC |
| *Megf10* | Forward | CGACAGATCCTGCCAGTGTTAC |
|  | Reverse | CAAAAGGCTCCGTTGTGGCAGT |
| *Mertk* | Forward | ATCATCCTCGGCTGCTTCTGTG |
|  | Reverse | ACGACCAGTTGGGAATCCTCCT |
| *Syp* | Forward | GACTATGGGCAGCAAGGCTA |
|  | Reverse | GATATGGGGATGGGAAAAGG |
| *Psd95* | Forward | TCAGACGGTCACGATCATCGCT |
|  | Reverse | GTTGCTTCGCAGAGATGCAGTC |
| *Homer1* | Forward | CCCTCTCTCATGCTAGTTCAGC |
|  | Reverse | GCACAGCGTTTGCTTGACT |
| *18s* | Forward | CCATCCAATCGGTAGTAGCG |
|  | Reverse | GTAACCCGTTGAACCCCATT |

**Table.S8 The antibodies used in the current study.**

| **Antibodies** | **Source** | **Identifier** |
| --- | --- | --- |
| IBA-1 | Abcam | ab178846 |
| Synaptophysin | Abcam | ab32127 |
| PSD95 | Thermo Fisher | 20665-1-AP |
| CD68 | Cell Signaling Technology | CST97778 |
| CYP1a1 | Thermo Fisher | 13241-1-AP |
| NLRP3 | Immunoway | YM8024 |
| Caspase1 | Cell Signaling Technology | 24232S |
| Cleaved-Caspase1 | Cell Signaling Technology | 89332S |
| IL18 | Cell Signaling Technology | 57058S |
| NFκB | Cell Signaling Technology | 8242T |
| β-actin | Cell Signaling Technology | 4970S |
| Anti-rabbit IgG | ABclonal | AS014 |
